# A novel subgenotype C6 Enterovirus A71 originating from the recombination between subgenotypes C4 and C2 strains in mainland China

**DOI:** 10.1038/s41598-021-04604-x

**Published:** 2022-01-12

**Authors:** Yongjuan Liu, Jingyi Zhou, Guangquan Ji, Yupeng Gao, Chunyan Zhang, Ting Zhang, Juan Huo, Wenxue Liang, Jin Yang, Yingying Shi, Shaolin Zhao

**Affiliations:** 1 Department of Central Laboratory, The First People’s Hospital of Lianyungang, Lianyungang, 222000 Jiangsu People’s Republic of China; 2 Department of Clinical Laboratory, The First Affiliated Hospital of Kangda College of Nanjing Medical University, Lianyungang, 222000 Jiangsu People’s Republic of China; 3 Department of Central Laboratory, The Affiliated Lianyungang Hospital of Xuzhou Medical University, Lianyungang, 222000 Jiangsu People’s Republic of China; 4 Department of Anorectal Surgical, The First People’s Hospital of Lianyungang, Lianyungang, 222000 Jiangsu People’s Republic of China; 5 Department of Science and Technology, The First People’s Hospital of Lianyungang, Lianyungang, 222000 Jiangsu People’s Republic of China; 6 Department of Clinical Laboratory, Lianyungang Center for Disease Control and Prevention, Lianyungang, 222000 Jiangsu People’s Republic of China; 7 Department of Clinical Laboratory, The First People’s Hospital of Lianyungang, No. 182, Tongguan Road, Haizhou District, Lianyungang, 222000 Jiangsu People’s Republic of China; 8 Department of Immunology, Jianghan University, No. 10, Erudite Road, Caidian District, Wuhan, 430056 Hubei People’s Republic of China

**Keywords:** Viral epidemiology, Viral evolution, Phylogenetics, Viral genetics

## Abstract

Recombination plays important roles in the genetic diversity and evolution of Enterovirus A71 (EV-A71). The phylogenetics of EV-A71 in mainland China found that one strain DL71 formed a new subgenotype C6 with unknown origin. This study investigated the detailed genetic characteristics of the new variant. DL71 formed a distinct cluster within genotype C based on the genome and individual genes (5′UTR, VP4, VP1, 2A, 2B, 2C, 3D, and 3′UTR). The average genetic distances of the genome and individual genes (VP3, 2A, 2B, 2C, 3A, 3C, and 3D) between DL71 and reference strains were greater than 0.1. Nine recombination events involving smaller fragments along DL71 genome were detected. The strains Fuyang-0805a (C4) and Tainan/5746/98 (C2) were identified as the parental strains of DL71. In the non-recombination regions, DL71 had higher identities with Fuyang-0805a than Tainan/5746/98, and located in the cluster with C4 strains. However, in the recombination regions, DL71 had higher identities with Tainan/5746/98 than Fuyang-0805a, and located in the cluster with C2 strains. Thus, DL71 was a novel multiple inter-subgenotype recombinant derived from the dominant subgenotype C4 and the sporadic subgenotype C2 strains. Monitoring the emergence of new variants by the whole-genome sequencing remains essential for preventing disease outbreaks and developing new vaccines.

## Introduction

As a neurotropic virus, Enterovirus A71 (EV-A71) remains an important pathogen of severe and fatal hand, foot and mouth disease (HFMD)^1,2^. Several outbreaks with a large number of central nervous system-complicated cases and deaths have occurred since the 1990s, particularly in the Asia–Pacific region^3^. In the past decade, China had the highest number of EV-A71-associated HFMD outbreaks and the best epidemiological monitoring records^3^. Approximately 13.7 million HFMD cases (including 3322 deaths) were reported in mainland China during 2008–2015^4^. And since EV-A71 vaccines have been put on the market, the epidemics had resulted in nearly 9.5 million cases (including 358 deaths) in mainland China during 2016–2020 (http://www.nhc.gov.cn/jkj/s2907/new_list_2.shtml).

EV-A71 is a member of Enterovirus genus in the Picornaviridae family. The viral genome is a single-stranded, positive sense RNA nearly 7.4 kb, which consists of an open reading frame (ORF) and the 5′- and 3′- untranslated regions (UTRs)^5^. The ORF (including P1, P2 and P3 regions) encodes a single polyprotein that will be cleaved into 4 structural proteins, VP1-VP4, and 7 non-structural proteins, 2A-2C and 3A-3D^6^. Based on the VP1 nucleotide sequences, EV-A71 is currently classified into 8 genotypes, A-H. Genotype A includes the sole prototype strain (BrCr) isolated in 1969^7^ and several strains reemerged in mainland China during 2008–2010^8^. Genotypes B and C are both further subdivided into six subgenotypes, designated B0–B5 and C0–C5, respectively^9^. Subgenotypes B4, B5 and C4 circulate mainly in eastern and southeast Asia, whereas C1 and C2 are prevalent in Europe^10^. Genotypes D and G have been identified in India^11^. Genotypes E, F and H emerged in Africa^12^, Madagascar^13^ and Pakistan^14^, respectively.

Genotypic replacements frequently occur in many countries and areas^3,15,16^, such as Taiwan^17^, Japan^18^, Malaysia^19^, Vietnam^20^, Thailand^21^, France^22^, Netherlands^23^, the USA^24^ and so on. However, subgenotype C4 has been the unique predominant genetic lineage in mainland China since 1998^25^. Unlike the evolution of several alternant predominant subgenotypes in some countries and areas, the sole predominant subgenotype C4 circulated in mainland China shown the genetic diversity of intra-subgenotype during continuous evolution^15^. The subgenotype C4 in mainland China has evolved into 3 clades, C4a1, C4a2, and C4b. And 3 shifts in the predominant subgenotype between C4b and C4a2 occurred respectively in 2003, 2004, and 2005^25^. Except subgenotype C4, other genotypes/subgenotypes A, B5, C0, C2 and C3 had sporadically emerged in mainland China^25^.

Co-circulation of EV-A71 belonging to various genotypes/subgenotypes may increase the possibility of recombination. Recent outbreaks were associated with newly emerging strains, including the recombinant C2-like strains in the Philippines^26^ and C1-like strains in Denmark^27^, Germany^28,29^, Spain^30^ and France^31^. The kinds of subgenotypes of EV-A71 in some countries where EV-A71 shared high genetic diversity might be underestimated. Our previous longitudinal study on the molecular epidemiology of EV-A71 in mainland China from 1987 to 2017 found that a new subgenotype containing an orphan strain (DL71, Dalian city, 2012) distinct from the previously known subgenotypes, designated C6^25^. Interestingly, an incredible recombination of DL71 occurred between subgenotypes C4 and C2 EV-A71 with multiple breakpoints^25^. However, the detailed information on recombination in DL71 is unclear. Thus, to provide a better understanding of this phenomenon, the present study aimed to investigate the genomic and evolutionary characteristics of DL71 at a genome-wide level. Clarifying the genetic variation and evolutionary relationship of new variants is important for disease prevention and vaccines development.

## Results

### Capsid VP1 gene characterization of the new subgenotype C6

**Figure 1:**
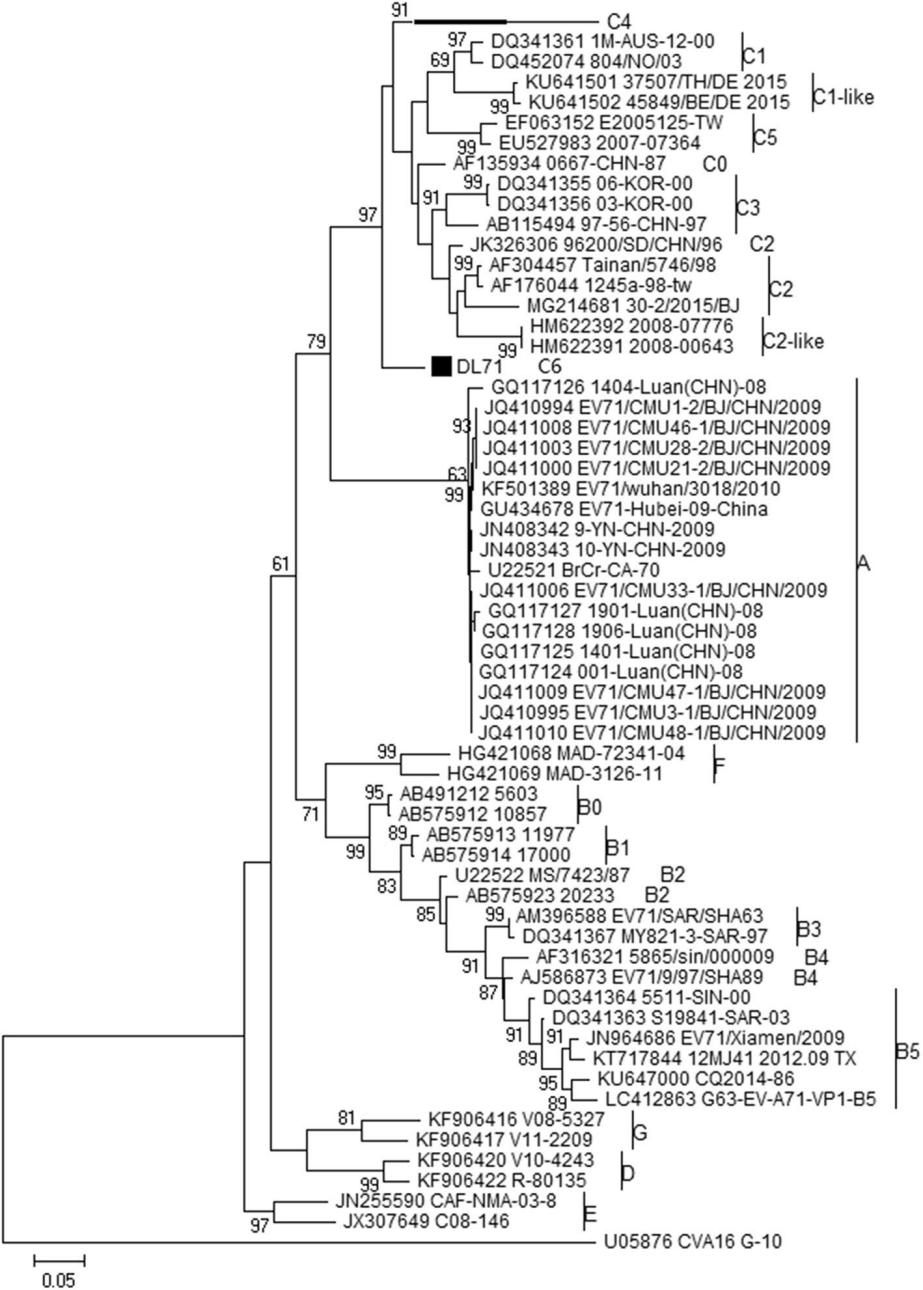
Phylogenetic tree based on the VP1 sequences of EV-A71. The evolutionary tree was constructed using the maximum likelihood method with the General Time Reversible (GTR) model by MEGAX. Bootstrap values were calculated by 1000 replicates. Bootstrap values lower than 60% are hidden. The branch of subgenotype C4 is compressed to save space. The solid square (black filled square) indicates the new subgenotype C6 EV-A71 strain DL71. CVA16 G-10 was used as the out-group strain. The scale bar indicates the number of nucleotide substitutions per site.

Phylogenetic analysis was performed using the VP1 sequences of 3036 Chinese EV-A71 strains and 34 reference strains. The cladogram showed that except the predominant subgenotype C4 and sporadic subgenotypes (A, C0, C2, C3, and B5), an independent branch contained the orphan strain DL71 was located within genotype C with 97% bootstrap value but distinct from the known subgenotypes C0–C5, C1-like, and C2-like (Fig. 1). The novel branch was defined as subgenotype C6.

### Genome sequence comparison of DL71

The complete genome and individual gene sequences of DL71 were compared to 221 EV-A71 reference strains belonging to different genotypes and subgenotypes. The numbers of base and amino acid substitutions per site from averaging over all sequence pairs between groups are shown in Table 1. The average genetic distance of nucleotide and amino acid sequences in different gene fragment between DL71 and these reference strains was calculated. DL71 has the smallest genetic distance with subgenotype C4 EV-A71 strains in nucleotide sequences of the genome (0.1280), 5′UTR (0.0677), VP2 (0.0728), VP3 (0.1277), 2C (0.1144), 3A (0.1131), 3B (0.0776) and 3C (0.1586). DL71 has the smallest genetic distance with subgenotype C2 EV-A71 strains in VP4 (0.0762), VP1 (0.0714), 2A (0.1219), 3D (0.1441) and 3′UTR (0.0412). And DL71 shares the smallest genetic distance with subgenotype C2-like EV-A71 strains in 2B (0.1790). A previous study has reported that the inter-genotype, inter-subgenotype and intra-subgenotype mean divergences of EV-A71 whole-genome nucleotide sequences were 0.17–0.22, 0.1–0.14 and 0.01–0.1, respectively^32^. However, the smallest genetic distances of the genome, VP3, 2C, 3A and 3C between DL71 and C4 strains, the smallest genetic distances of 2A and 3D between DL71 and C2 strains, and the smallest genetic distances of 2B between DL71 and C2-like strains were all more than 0.1. Moreover, the average genetic distances of the genomes and individual genes between DL71 and these reference strains (Table 1) were all higher than those within genotypes/subgenotypes (Supplementary Table S3). Thus, the results further demonstrated that DL71 belonged to a new subgenotype C6.

**Table 1:** Average genetic distances of nucleotide and amino acid sequences in different gene fragment between DL71 and other genotypes or subgenotypes of EV-A71.

Genotypes		Genome	5'UTR	VP4	VP2	VP3	VP1	2A	2B	2C	3A	3B	3C	3D	3'UTR
A	nt	0.2340	0.1845	0.2123	0.2061	0.2043	0.2001	0.2500	0.2756	0.2575	0.3183	0.3855	0.2931	0.2633	0.1646
	aa	0.0531		0.0148	0.0254	0.0538	0.0366	0.0619	0.0448	0.0289	0.1105	0.1732	0.0996	0.0680	
B0	nt	0.2106	0.1557	0.1869	0.1688	0.2571	0.1768	0.2129	0.2889	0.1971	0.2771	0.2869	0.2627	0.2226	0.5353
	aa	0.0465		0.0146	0.0281	0.0574	0.0273	0.0270	0.0465	0.0216	0.0976	0.1542	0.0796	0.0660	
B1	nt	0.2103	0.1573	0.2216	0.1692	0.2515	0.1900	0.2077	0.2754	0.1940	0.2413	0.1570	0.2534	0.2556	0.1190
	aa	0.0485		0.0	0.0342	0.0576	0.0325	0.0272	0.0574	0.0325	0.1237	0.1001	0.0678	0.0625	
B2	nt	0.2179	0.1719	0.2397	0.1860	0.2378	0.1848	0.1978	0.2678	0.2013	0.2294	0.2189	0.2702	0.2638	0.1194
	aa	0.0513		0.0147	0.0301	0.0618	0.0308	0.0443	0.0518	0.0452	0.1105	0.1557	0.0678	0.0614	
B3	nt	0.2132	0.1464	0.2509	0.2133	0.2463	0.1885	0.2162	0.2835	0.2166	0.2733	0.2839	0.2453	0.2143	0.2097
	aa	0.0413		0.0073	0.0260	0.0640	0.0308	0.0408	0.0308	0.0263	0.0977	0.1542	0.0504	0.0443	
B4	nt	0.2155	0.1541	0.2365	0.2048	0.2557	0.1881	0.2349	0.2706	0.2052	0.2213	0.2189	0.2379	0.2485	0.1564
	aa	0.0494		0.0	0.0281	0.0684	0.0273	0.0408	0.0518	0.0373	0.1302	0.1271	0.0796	0.0545	
B5	nt	0.2166	0.1578	0.2318	0.1997	0.2493	0.1862	0.2200	0.2935	0.2098	0.2447	0.2072	0.2485	0.2491	0.1632
	aa	0.0489		0.0	0.0281	0.0640	0.0273	0.0408	0.0518	0.0341	0.1105	0.1001	0.0915	0.0568	
C1	nt	0.1727	0.1500	0.1744	0.1278	0.1615	0.1153	0.1362	0.2177	0.2183	0.3624	0.4094	0.2325	0.1764	0.0975
	aa	0.0385		0.0036	0.0139	0.0400	0.0085	0.0288	0.0518	0.0349	0.1812	0.1542	0.0533	0.0437	
C1-like	nt	0.2001	0.1855	0.1556	0.1609	0.1845	0.1336	0.1551	0.2829	0.2307	0.3025	0.4949	0.3226	0.1985	0.2644
	aa	0.0362		0.0	0.0171	0.0426	0.0142	0.0229	0.0444	0.0253	0.1028	0.1999	0.0678	0.0422	
C2	nt	0.1403	0.1087	**0.0762**	0.1065	0.1379	**0.0714**	**0.1219**	0.1803	0.1968	0.3819	0.3800	0.2069	**0.1441**	**0.0412**
	aa	0.0367		0.0029	0.0095	0.0379	0.0064	0.0257	0.0476	0.0420	0.1615	0.1542	0.0563	0.0391	
C2-like	nt	0.1763	0.1774	0.1068	0.1032	0.1426	0.0997	0.1599	**0.1790**	0.2204	0.3214	0.2660	0.2695	0.2177	0.4279
	aa	0.0348		0.0	0.0079	0.0393	0.0124	0.0431	0.0412	0.0237	0.0849	0.1001	0.0678	0.0465	
C3	nt	0.1620	0.1024	0.0996	0.1202	0.1423	0.1072	0.1605	0.2101	0.2093	0.3602	0.3818	0.2232	0.1881	0.0502
	aa	0.0396		0.0	0.0119	0.0379	0.0136	0.0443	0.0412	0.0310	0.1639	0.1542	0.0649	0.0465	
C4	nt	**0.1280**	**0.0677**	0.1098	**0.0728**	**0.1277**	0.1066	0.1501	0.2118	**0.1144**	**0.1131**	**0.0776**	**0.1586**	0.2000	0.2434
	aa	0.0344		0.0029	0.0141	0.0391	0.0170	0.0443	0.0441	0.0142	0.0938	0.0595	0.0564	0.0481	
C5	nt	0.1833	0.1133	0.1118	0.1331	0.1850	0.1211	0.1574	0.2596	0.2196	0.4339	0.2916	0.2428	0.2297	0.0913
	aa	0.0427		0.0	0.0119	0.0379	0.0187	0.0478	0.0519	0.0373	0.1571	0.1542	0.0533	0.0556	
E	nt	0.2304	0.1598	0.2069	0.2061	0.2564	0.1938	0.2372	0.2701	0.2310	0.3628	0.3427	0.3067	0.2496	0.1476
	aa	0.0501		0.0294	0.0281	0.0509	0.0377	0.0619	0.0412	0.0310	0.0976	0.1542	0.0915	0.0556	
F	nt	0.2359	0.1658	0.2376	0.2212	0.2253	0.2067	0.2283	0.3038	0.2489	0.3611	0.3499	0.2850	0.2653	0.1166
	aa	0.0483		0.0147	0.0281	0.0541	0.0299	0.0548	0.0360	0.0247	0.1270	0.1542	0.0722	0.0631	

The average genetic distance of nucleotide and amino acid sequences in different gene fragment between DL71 and the reference strains was estimated by the DISTANCE program (Compute between Group Mean Distance) in MEGAX. The numbers of base and amino acid substitutions per site from averaging over all sequence pairs between groups are shown. The nucleotide sequences comparison was conducted using the Kimura 2-parameter model with both transitions and transversions substitutions. The amino acid sequences comparison was conducted using the Poisson correction model with all substitution. The bootstrap method with 1000 replications was selected as the variance estimation method. The smallest genetic distances of nucleotide sequences in different regions are marked in bold. The information of 222 EV-A71 strains used in the analysis was listed in the supplementary table S2. nt, nucleotide. aa, amino acid.

### Phylogenetic analysis of the complete genome and individual genes

**Figure 2:**
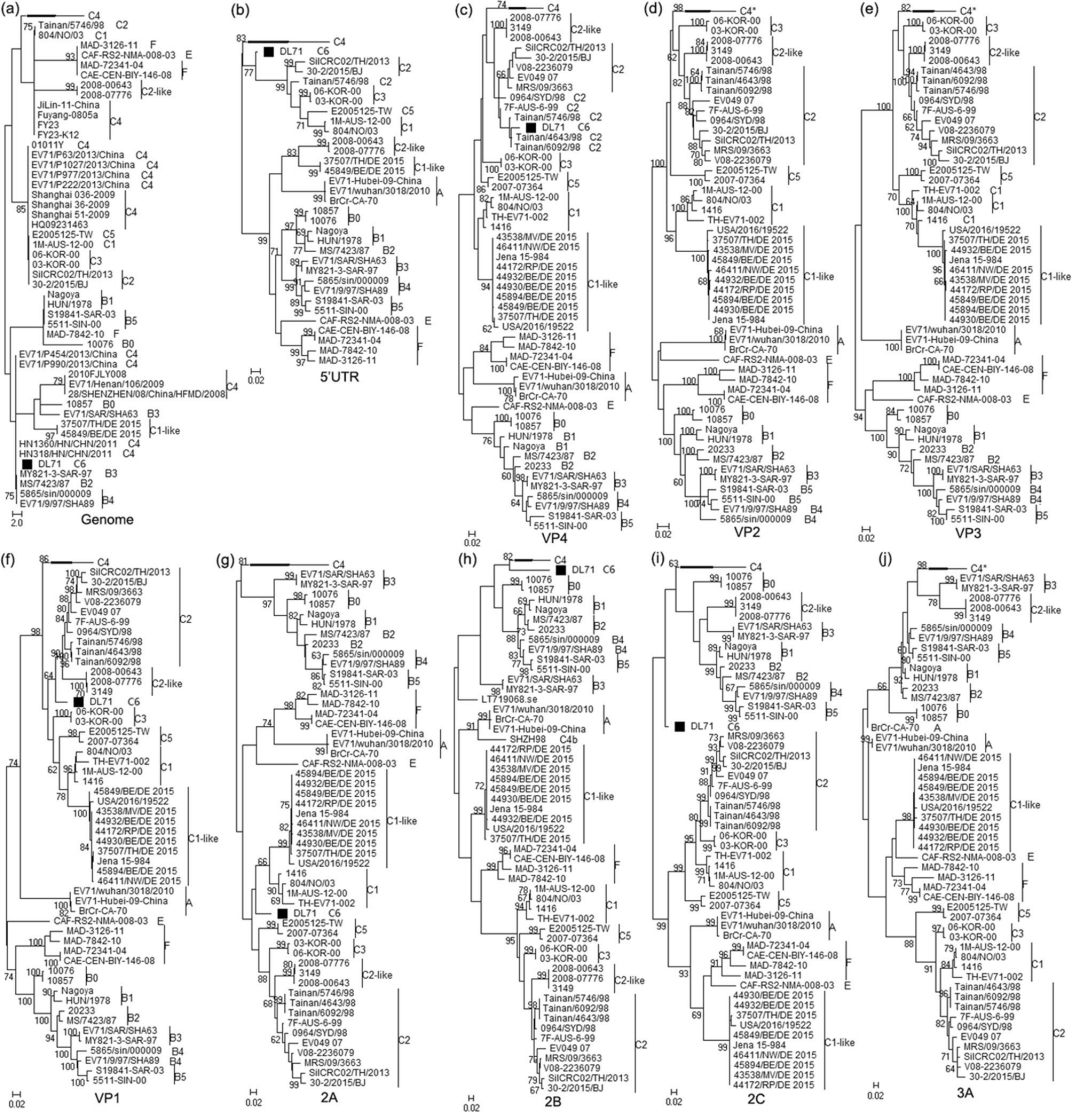

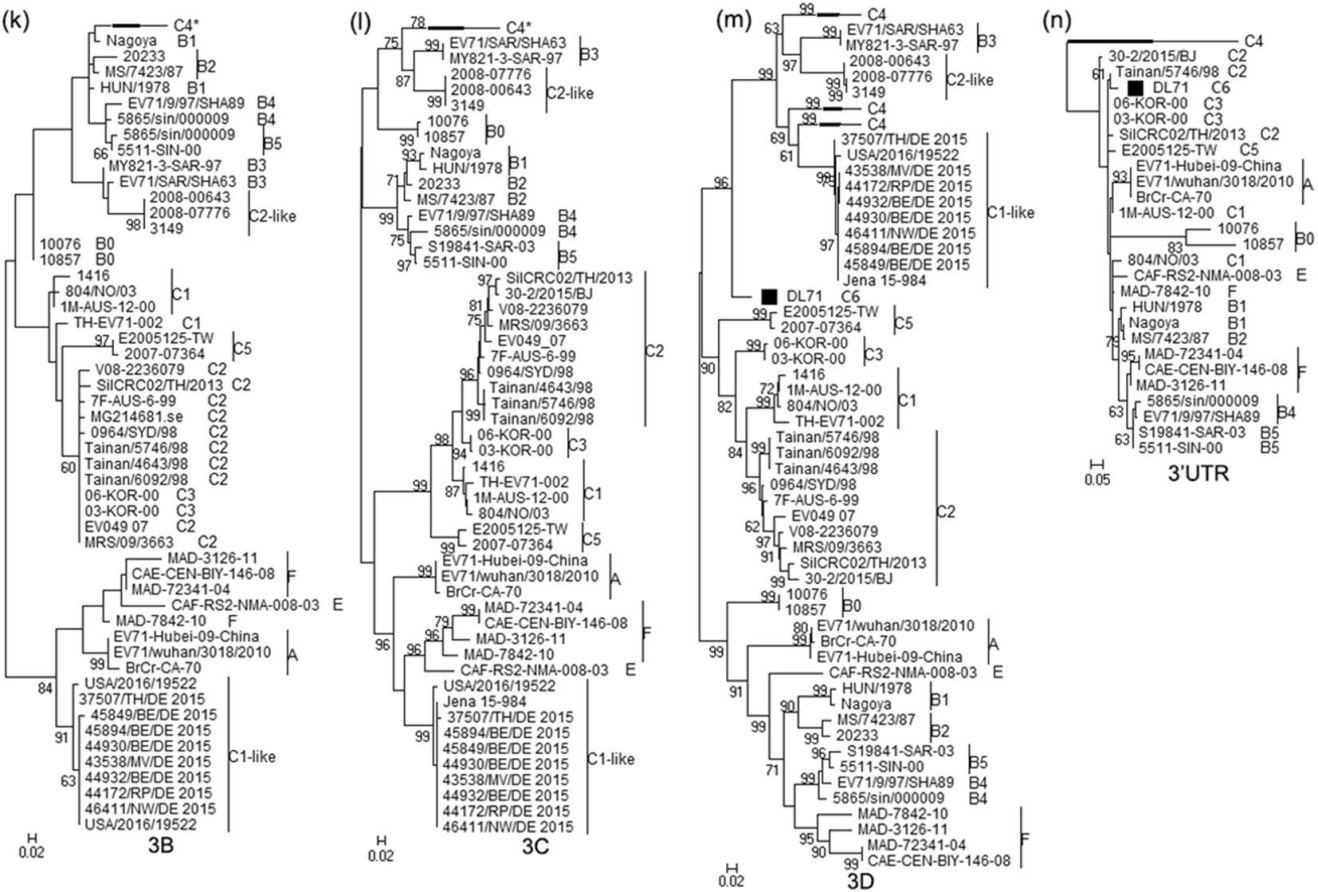
Phylogenetic trees based on the complete genomes and individual genes of DL71 and EV-A71 reference strains. The evolutionary trees based on (**a**) the complete genomes and individual genes (**b**) 5′UTR, (**c**) VP4, (**d**) VP2, (**e**) VP3, (**f**) VP1, (**g**) 2A, (**h**) 2B, (**i**) 2C, (**j**) 3A, (**k**) 3B, (**l**) 3C, (**m**) 3D and (**n**) 3′UTR were constructed using the maximum likelihood method with the GTR model by MEGAX. Bootstrap values lower than 60% are hidden. DL71 is labeled with a solid square (black filled square). The asterisk (*) indicates that DL71 is located within the branch of subgenotype C4 in the phylogenetic trees based on (**d**) VP2, (**e**) VP3, (**j**) 3A, (**k**) 3B, and (**l**) 3C.

To assess the genetic relationships between DL71 and EV-A71 reference strains, phylogenetic trees based on the genome and individual gene sequences were constructed by the maximum likelihood method with the GTR model. As shown in Fig. 2, except that the VP2, VP3, 3A, 3B, and 3C of DL71 are located in the same evolutionary branch with subgenotype C4 strains, the genome and individual genes 5′UTR, VP4, VP1, 2A, 2B, 2C, 3D and 3′UTR of DL71 formed a new independent branch in phylogenies. Combining the results of the sequence comparison and phylogenetic analysis, it is reasonable to assume that the subgenotype C6 is a novel genetic lineage of EV-A71 in mainland China different from the previously known genetic lineages.

### Genomic recombination analysis of DL71

**Figure 3:**
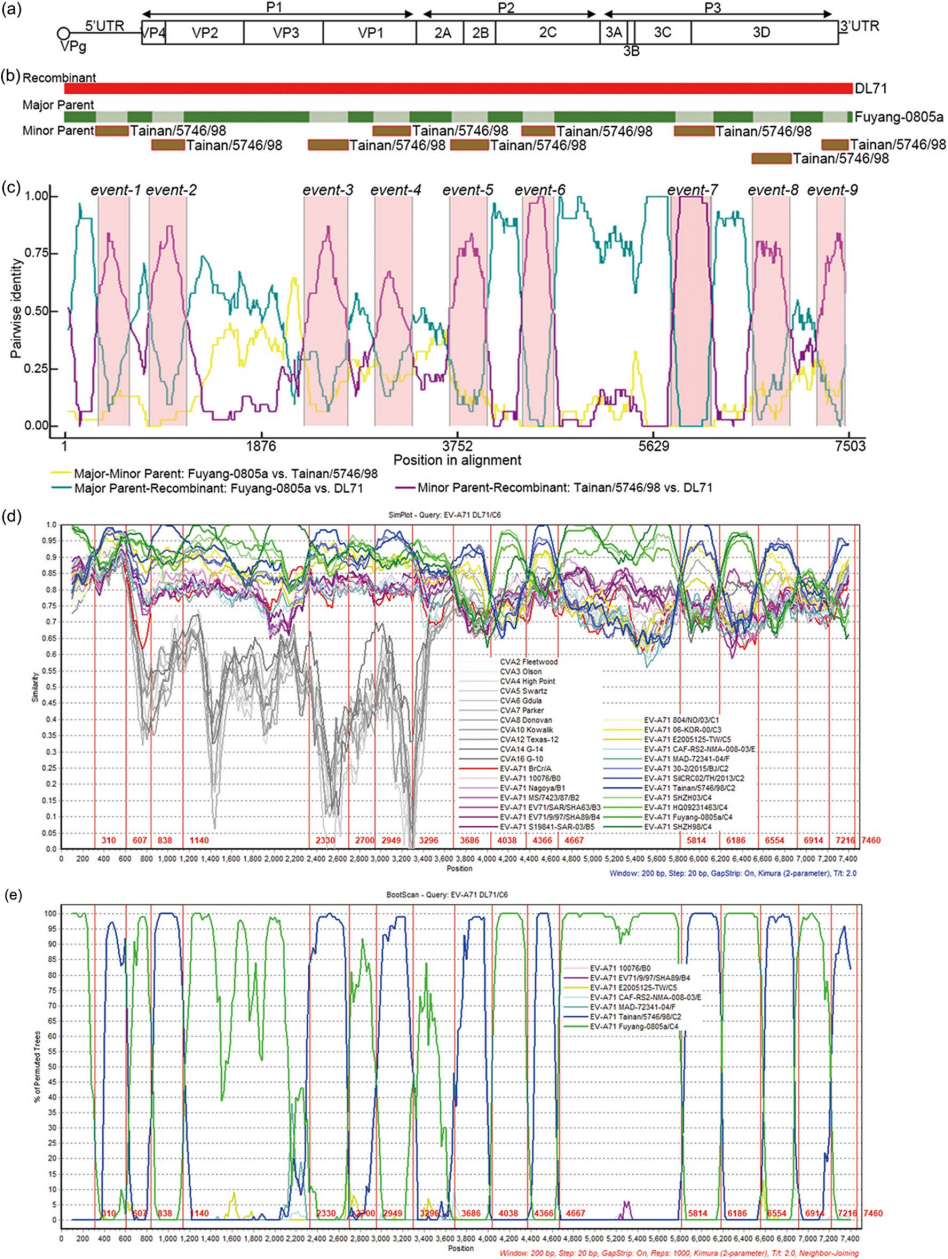
Recombination analyses of DL71. (**a**) Graphical representation of EV-A71 genome. (**b**) A schematic map of recombination within DL71 genome. DL71 genome is shown as a red rectangle. The backbone of DL71 is derived from the major parent (green rectangles), while other genetic components of DL71 is derived from the minor parent (brown rectangles). (**c**) The plot display of 9 recombination events in DL71 genome identified by RDP4. (**d**) The genome of DL71 was used as the query sequence in the Similarity plot analysis. (**e**) The genome of DL71 was used as the query sequence in the BootScan analysis. The vertical red line indicates the breakpoint regions.

To detect the recombination events in DL71 genome, the genome sequence alignment dataset of DL71 and 42 reference strains was analyzed by RDP4. According to the structure of EV-A71 full genome (Fig. 3a), the schematic sequence display (Fig. 3b) and the pairwise identity plot (Fig. 3c) of RDP4 showed that DL71 was a recombinant strain formed by 9 putative recombination events between a major parental strain Fuyang-0805a and a minor parental strain Tainan/5746/98, which belonged to subgenotypes C4 and C2, respectively. The average p-values of 7 algorithms were utilized to confirm the potential recombination events (Table 2). The recombination scores of 9 recombination events were all more than 0.5 (Table 2). Moreover, the recombination regions 310–607, 838–1140, 2330–2700, 2949–3296, 3686–4038, 4366–4667, 5814–6186, 6554–6914, and 7216–7460 nt of DL71 in the alignment with gaps shared more identities with the minor parent Tainan/5746/98 than the major parent Fuyang-0805a, whereas other regions of DL71 shared more identities with Fuyang-0805a than Tainan/5746/98 (Table 3).

**Table 2:** Recombination events detected with RDP4 from the alignment of the complete genome of DL71.

Events	Breakpoints in the alignment with gaps (without gaps)	Parent major/minor	Recombinant score	Recombination detection methods (average *p* value)						
				RDP	GENECONV	BootScan	MaxChi	Chimaera	SiScan	3Seq
Event-1	310–607 (299–592)	Fuyang-0805a/Tainan/5746/98	0.691	1.687 × 10^−09^	1.752 × 10^−07^	1.775 × 10^−08^	5.459 × 10^−05^	1.202 × 10^−04^	1.141 × 10^−05^	2.740 × 10^−12^
Event-2	838–1140 (812–1114)	Fuyang-0805a/Tainan/5746/98	0.671	2.987 × 10^−14^	1.234 × 10^−21^	1.536 × 10^−14^	8.831 × 10^−05^	7.272 × 10^−05^	2.741 × 10^−08^	2.740 × 10^−12^
Event-3	2330–2700 (2294–2661)	Fuyang-0805a/Tainan/5746/98	0.662	8.256 × 10^−13^	2.653 × 10^−10^	7.735 × 10^−12^	–	–	3.831 × 10^−05^	2.740 × 10^−12^
Event-4	2949–3296 (2906–3253)	Fuyang-0805a/Tainan/5746/98	0.690	1.231 × 10^−08^	7.244 × 10^−03^	2.524 × 10^−07^	–	–	–	1.618 × 10^−07^
Event-5	3686–4038 (3601–3953)	Fuyang-0805a/Tainan/5746/98	0.679	2.464 × 10^−33^	5.639 × 10^−42^	3.991 × 10^−15^	1.502 × 10^−13^	4.927 × 10^−13^	1.537 × 10^−11^	2.740 × 10^−12^
Event-6	4366–4667 (4281–4582)	Fuyang-0805a/Tainan/5746/98	0.679	2.657 × 10^−19^	1.616 × 10^−41^	7.154 × 10^−20^	8.852 × 10^−12^	4.633 × 10^−12^	4.336 × 10^−16^	1.370 × 10^−12^
Event-7	5814–6186 (5728–6100)	Fuyang-0805a/Tainan/5746/98	0.613	2.731 × 10^−31^	2.715 × 10^−33^	3.467 × 10^−32^	3.054 × 10^−18^	4.074 × 10^−18^	6.749 × 10^−23^	1.370 × 10^−12^
Event-8	6554–6914 (6468–6828)	Fuyang-0805a/Tainan/5746/98	0.617	4.717 × 10^−16^	8.786 × 10^−50^	8.420 × 10^−16^	7.382 × 10^−13^	7.403 × 10^−14^	5.832 × 10^−14^	2.740 × 10^−12^
Event-9	7216–7460 (7130–7374)	Fuyang-0805a/Tainan/5746/98	0.652	2.683 × 10^−13^	1.061 × 10^−07^	8.214 × 10^−13^	2.101 × 10^−05^	1.578 × 10^−05^	1.262 × 10^−09^	2.740 × 10^−12^

**Table 3:** Nucleotide sequence identities of the non-recombination and recombination regions between DL71 and its putative parental strains.

Non-recombination regions in alignment with gaps (nt)	Parent major/minor	Identity	Recombination regions in alignment with gaps (nt)	Parent major/minor	Identity
1–309	Fuyang-0805a/ Tainan/5746/98	**96.6%** 84.5%	310–607	Fuyang-0805a/ Tainan/5746/98	90.1% **98.0%**
608–837	Fuyang-0805a/ Tainan/5746/98	**95.0%** 89.0%	838–1140	Fuyang-0805a/ Tainan/5746/98	86.5% **99.7%**
1141–2329	Fuyang-0805a/ Tainan/5746/98	**93.9%** 88.9%	2330–2700	Fuyang-0805a/ Tainan/5746/98	91.0% **97.6%**
2701–2948	Fuyang-0805a/ Tainan/5746/98	**94.3%** 90.2%	2949–3296	Fuyang-0805a/ Tainan/5746/98	91.4% **96.0%**
3297–3685	Fuyang-0805a/ Tainan/5746/98	**89.9%** 86.2%	3686–4038	Fuyang-0805a/ Tainan/5746/98	75.9% **91.8%**
4039–4365	Fuyang-0805a/ Tainan/5746/98	**96.9%** 72.8%	4366–4667	Fuyang-0805a/ Tainan/5746/98	82.1% **99.7%**
4668–5813	Fuyang-0805a/ Tainan/5746/98	**96.2%** 76.0%	5814–6186	Fuyang-0805a/ Tainan/5746/98	76.1% **99.7%**
6187–6553	Fuyang-0805a/ Tainan/5746/98	**97.0%** 74.7%	6554–6914	Fuyang-0805a/ Tainan/5746/98	76.7% **93.9%**
6915–7215	Fuyang-0805a/ Tainan/5746/98	**85.7%** 81.1%	7216–7460	Fuyang-0805a/ Tainan/5746/98	77.0% **93.1%**

The highest nucleotide identities of different regions are marked in bold.

To display the recombination results of RDP4, the genome sequence of DL71was used as the query sequence in Simplot analysis. The recombination signals in DL71 genome exhibited by SimPlot were similar to those detected by RDP4. The similarity plot analysis showed that the sequence of DL71 between every two recombination regions shared higher similarity with Fuyang-0805a than Tainan/5746/98. However, the sequence of DL71 within every recombination region shared higher similarity with Tainan/5746/98 than Fuyang-0805a (Fig. 3d). The recombination diagram shown in the bootscan analysis further confirmed these recombination events (Fig. 3e).

**Figure 4:**
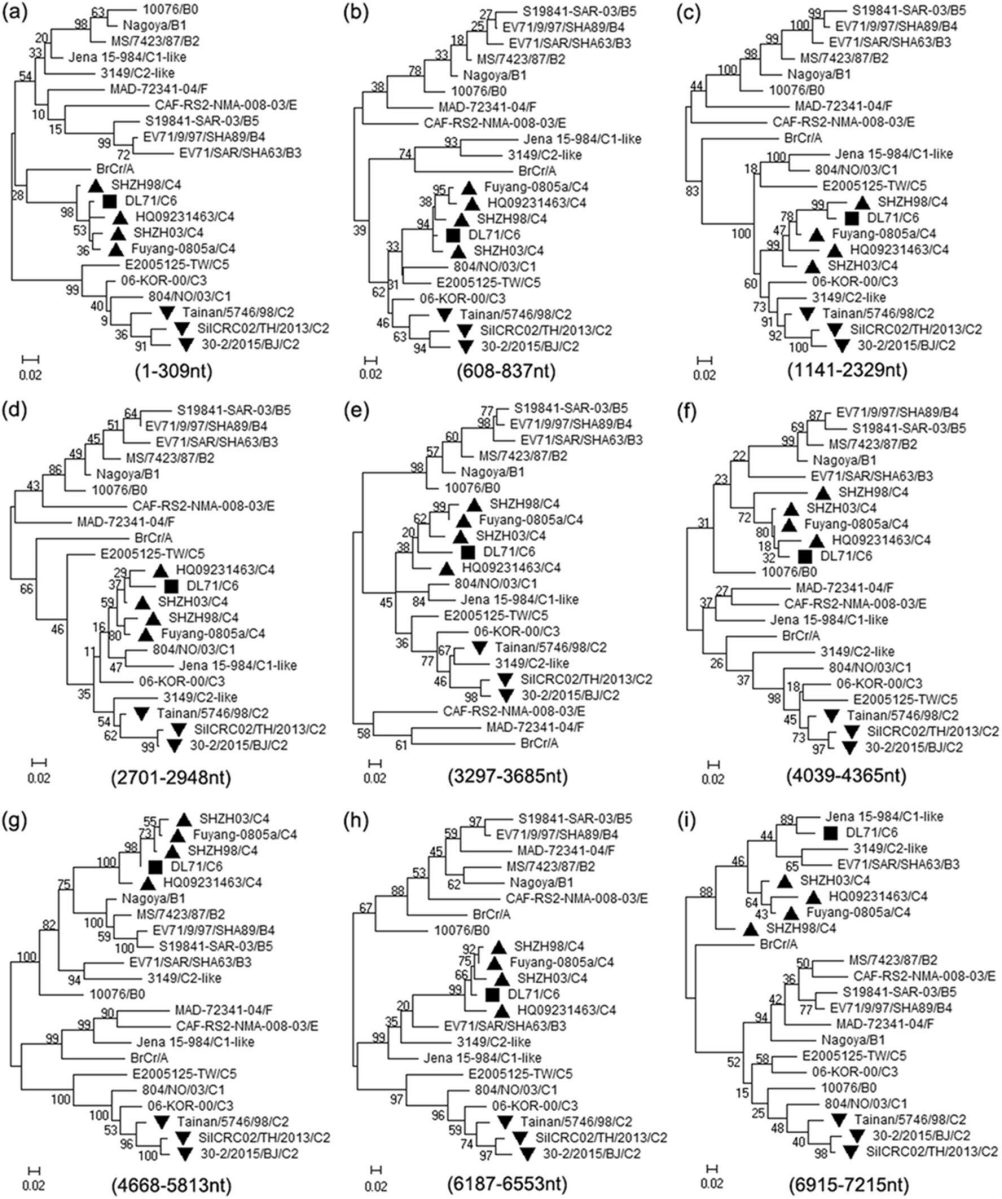
Phylogenetic trees based on the non-recombination regions of DL71. The regions (**a**) 1–309 bp, (**b**) 608–837 bp, (**c**) 1141–2329 bp, (**d**) 2701–2948 bp, (**e**) 3297–3685 bp, (**f**) 4039–4365 bp, (**g**) 4668–5813 bp, (**h**) 6187–6553 bp, and (**i**) 6915–7215 bp of DL71 and other reference strains were used to construct the phylogenetic trees by the maximum likelihood method with 1000 bootstrap replicates. The recombinant strain DL71 is labeled with a solid square (black filled square), the major parental strain with a solid regular triangle (black filled upward triangle), and the minor parental strain with a solid inverted triangle (black filled downward triangle).

**Figure 5:**
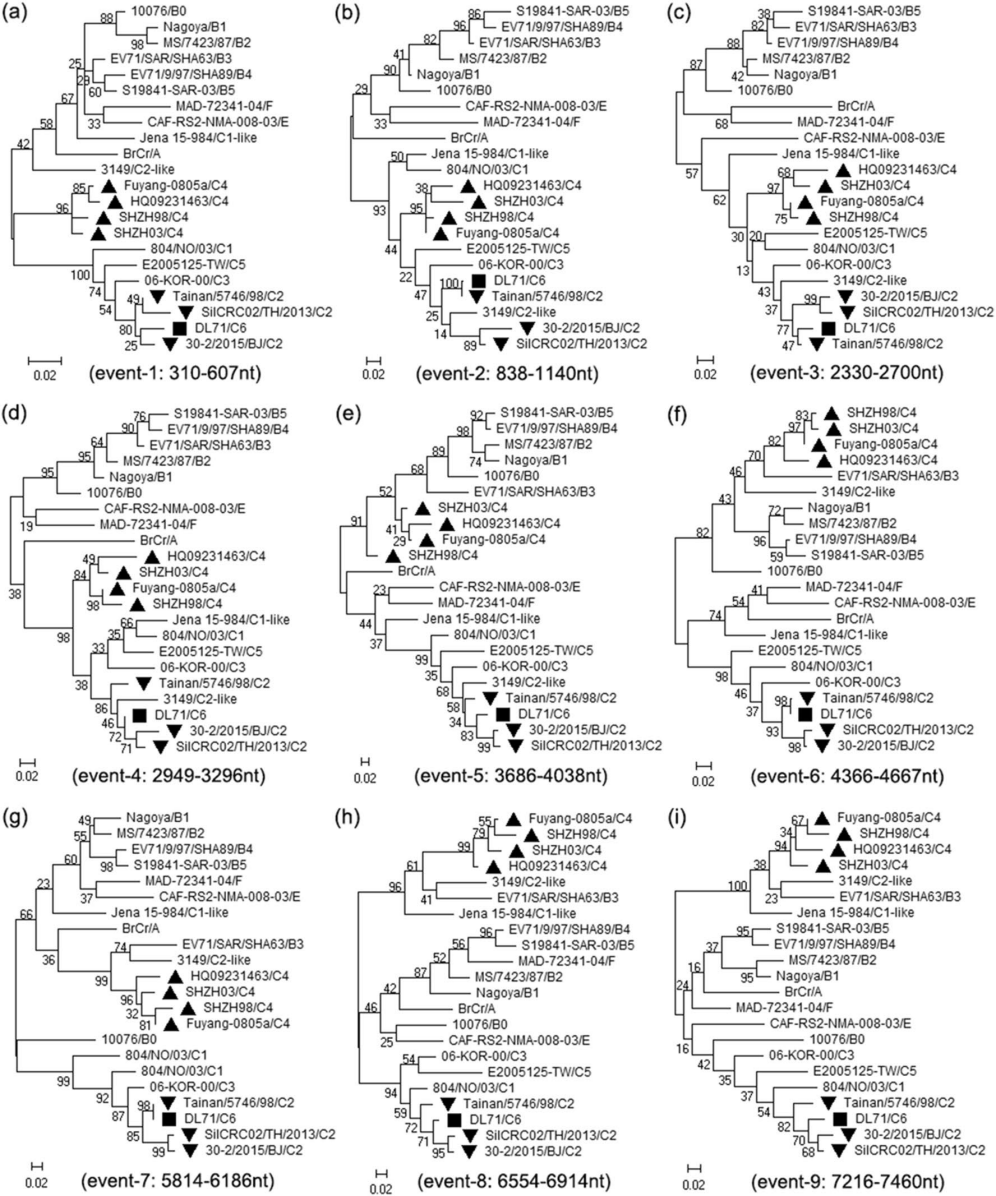
Phylogenetic trees based on the recombination regions of DL71. The regions (**a**) 310–607 bp, (**b**) 838–1140 bp, (**c**) 2330–2700 bp, (**d**) 2949–3296 bp, (**e**) 3686–4038 bp, (**f**) 4366–4667 bp, (**g**) 5814–6186 bp, (**h**) 6554–6914 bp, and (**i**) 7216–7460 bp of DL71 and other reference strains were used to construct the phylogenetic trees by the maximum likelihood method with 1000 bootstrap replicates. The recombinant strain DL71, the major parental strain and the minor parental strain are indicated with “black filled square”, “black filled upward triangle” and “black filled downward triangle”, respectively.

In addition, the authenticity of the recombination events within DL71 genome was proven by a set of statistically incongruent phylogenetic trees, an approach considered the gold-standard bioinformatics method for demonstrating the presence of recombination^33^. According to the locations of the recombination breakpoints in the alignment dataset in RDP4 sequence display, the locations of recombination regions in genome sequences without gaps were listed (Supplementary Table S5), which was convenient for building the corresponding sequence datasets of the recombination and non-recombination regions. The non-recombination regions of DL71 were clustered into subgenotype C4 strains (Fig. 4). However, the recombination regions of DL71 were clustered into subgenotype C2 strains (Fig. 5). The topological incongruence of phylogenetic trees reconfirmed that DL71 appeared with potential recombination events, and subgenotypes C4 and C2 EV-A71 strains participated in this process.

## Discussion

Our previous study found that a new subgenotype C6 of EV-A71 emerged in mainland China, which might derive from subgenotypes C4 and C2 EV-A71 strains^25^. However, the detailed genetic characteristics of the subgenotype C6 EV-A71 strain DL71 were unclear. Here we provided strong evidence to demonstrate that DL71 exactly formed a new branch distinct from the known subgenotypes by calculating the genetic distance and constructing the phylogenetic trees based on the genome and individual gene sequences. 9 recombination events, 2 parental strains and the breakpoints locations of DL71 were detected by RDP4 and SimPlot. Moreover, phylogenetic analysis and nucleotide sequence identity calculation based on the recombination and non-recombination regions further indicated that DL71 was a new variant originated from 9 inter-subgenotype recombination events between subgenotypes C4 and C2 EV-A71 strains.

In this study, DL71 exhibited a multiple inter-subgenotype recombination manner. The recombination events detected in DL71 genome was more than that previously reported in EV-A71 strains belonging to genotype A and subgenotypes C4, C2 and B5^25,34,35^. In total, 9 recombination events were detected in DL71 genome with multiple recombination breakpoints throughout the full genome. Numerous studies suggested that the recombination breakpoints in EV-A71 genome are frequently located in the non-structural regions 5′UTR, P2, and P3 but rarely in the structural region P1^25^. Consistent with previous findings, the breakpoints of recombination events event-1, event-5, event-6, event-7, event-8, and event-9 of DL71 were located in the 5′UTR, P2 and P3 regions. However, 6 breakpoints of recombination events event-2, event-3, and event-4 of DL71 were located in the P1 region. This study showed that three recombination events occurred in the structural regions of DL71. In addition, unlike the recombination of subgenotype C4 EV-A71 involved several viruses, such as CVA4, CVA5, CVA14, CVA16 and genotype B EV-A71^25^, only subgenotypes C4 and C2 EV-A71 respectively as the major and minor parents participate in the recombination of DL71. DL71 originated from 9 recombination events between subgenotypes C4 and C2 EV-A71 and presented a notable helix-like recombination manner.

The emergence of new variants has been reported continuously. For example, the subgenotype C2-like strains were detected in different geographic regions in the Philippines in 2000, 2002, 2005, 2010 and in Taiwan in 2008^26,36^. The C2-like strains originated from the recombination between subgenotypes C2 and B3 EV-A71 strains^36^. Moreover, the C1-like EV-A71 strains associated with neurologic symptoms have emerged in several outbreaks in Europe since 2007^27–31,37^. The C1-like strains shared the highest nucleotide similarity with subgenotype C1 EV-A71 strains in P1 region, while it shared higher nucleotide similarity with CVA6 and CVA8 in 5′UTR and higher nucleotide similarity with CVA2, CVA4, CVA5, and CVA6 in 3D region than subgenotype C1 EV-A71^31^. The C1-like EV-A71 was a multiple recombinant derived from the complex recombination among several viruses^31^. Except for the C2-like and C1-like strains, DL71 was identified as another new inter-subgenotype recombinant originated from the predominant subgenotype C4 and the sporadic circulating C2 strains. The comparison of the amino acid sequences in the complete genome between DL71 and EV-A71 strains belonging to subgenotypes C1–C5, C1-like, and C2-like showed that 129 amino acid residues were different. And if the comparison not include 2 strains (SHZH98/AF302996 and HQ09231463/JQ316638), 2 residues (143 N and 146H) in VP2, 9 residues (39G, 42H, 67G, 68G, 81G, 93H, 148A, 159S, and 161C) in VP3, 6 residues (32 K, 33C, 34A, 35S, 36 T, and 37A) in 2A, 2 residues (288E and 297F) in 2C, and 3 residues (2S, 3D, and111P) in 3C were unique to DL71 (Supplementary Table S6). This was the first report describing the detailed genetic characteristics of the novel subgenotype C6 of EV-A71 in mainland China. However, given that the C1-like and C2-like were also named subgenotype C6 respectively in one report^27^ and in two reports^38,39^, the subgenotype C6 described in the present study could be named C4-like to avoid confusion. The co-circulation of various genotypes/subgenotypes of EV-A71 in one country during the same period of time provides an environment for the emergence of new recombinant variants.

Recombination may endow newly emerging variants with different antigenicity or pathogenicity^40^. An antigenic map constructed using serological data suggested that the antigenicity varied among different genotypes and subgenotypes of EV-A71. The antigenic map showed that subgenotypes B1 and B4 strains were clustered closely together, subgenotypes C2 and C4 formed a separate cluster different from genotype B, and subgenotype B5 formed its own cluster^41^. Although the cross-neutralization by anti-EV-A71 antibodies was commonly observed among various genotypes^42^, there was a notable difference in neutralization titers. For example, children infected with genotype B strains showed higher neutralization titers against genotype B strains than genotype C strains^43^. The subgenotype B4 vaccine could not elicit an effective neutralization titer against subgenotype C2 strains^44^. The C2-like variant in Taiwan in 2008 had a 128-fold lower neutralization titer than the previous C2 strains^36^. The antigenicity difference between subgenotype C6 EV-A71 and its parental strains need to be further studied. The altered antigenicity of new variants may influence the circulation of EV-A71^29^. The subgenotype C4 EV-A71 has a large susceptible population in mainland China, whereas the C2 EV-A71 does not. As a recombinant derived from subgenotypes C4 and C2 strains, C6 EV-A71 emerged occasionally may be due to its antigenicity being different from C4 EV-A71 and less susceptible population. Moreover, the altered antigenicity of new variants may be associated with the severity of clinical symptoms^30^. The C1-like variant associated with neurologic complications has caused several outbreaks in European countries, such as Germany^29^, France^31^, Spain^30^ and Denmark^27^. According to the potential virulence determinants previously reported in EV-A71 genome, we have speculated that the C6 EV-A71 strain DL71 was probably a strain with low virulence^25^. In fact, there is no deterministic relationships between a specific genotype/subgenotype and viral virulence or certain clinical manifestations^45^. Both virus and host factors contribute to the progression of EV-A71-associated disease.

In addition, this study on DL71 was limited to the field of bioinformatics due to the lack of live isolate and clinical symptom information. There are 3 limitations in this study. First, only the orphan strain DL71 belonged to subgenotype C6 so far, making it hard to judge whether subgenotype C6 contained only one strain or other strains were omitted due to its low prevalence in actual circulation environment. Based on the current epidemiological data, the actual route of virus transmission cannot be traced. Second, as one important mechanism of EV-A71 evolution, recombination can lead to the altered antigenicity of new variants^36^. Whether the new subgenotype C6 strain DL71 shares similar phenotypic and antigenic characteristics with its parental strains is unknown. Both the genome sequence analysis and neutralization antibody titers determination are needed. Third, the only information about DL71 was its isolated place and time, whereas the clinical symptoms and disease severity of DL71 was unknown. Although the authors have analyzed the previously reported potential virulence determinants in DL71 genome^25^, it is still difficult to define the pathogenicity of DL71, which is associated with many factors, including virus virulence and host immunity. Although the value is limited for investigating the actual viral population by using the published genomes, this study revealed the recombination manner of the new subgenotype C6. The effect of mutation during EV-A71 evolution is undeniable. In fact, both recombination and mutation play important roles in the emergence of new variants.

Genetic recombination may occur more frequently than we expected. Since virus typing with a single short fragment will miss some new subgenotypes, the complete genome sequences should be used to classify EV-A71 when monitoring the molecular epidemiology. Moreover, considering that the available EV-A71 vaccines in mainland China target subgenotype C4 strains, it is necessary to developing a polyvalent vaccine against various strains. Thus, comprehensive surveillance of the genotype shifts, recombination, mutation and the altered antigenicity of EV-A71 is very important for preventing HFMD outbreaks and developing new treatment strategies.

## Materials and methods

### Sequence data and alignment

This study involved no human participants or human experimentation. The complete VP1 sequences of 3036 EV-A71 strains in mainland China during 1987–2017 were downloaded from GenBank database. The VP1 sequences of 34 international EV-A71 strains belonging to genotypes/subgenotypes A, B0-B5, C1-C3, C5, D, E, F, G, C1-like, and C2-like were used as the reference sequences. Coxsackievirus A group 16 (CVA 16) prototype strain G-10 was used as an out-group in the phylogenetic analysis. Thus, 3070 VP1 sequences (Supplementary Table S1) were used in the phylogenetic analysis to determine whether new genetic lineages of EV-A71 emerged in mainland China.

In these 3036 Chinese EV-A71 strains, the complete genome sequences of 174 EV-A71 strains were downloaded from GenBank database. The genome sequences of 38 international EV-A71 strains belonging to A, B0–B5, C1–C3, C5, E, F, C1-like, and C2-like were downloaded. The genome and individual gene sequences of 222 EV-A71 strains (Supplementary Table S2) were used to construct phylogenetic trees and calculate genetic distance to identify the novel subgenotype.

The genome and individual gene sequences were edited by the EditSeq program of DNAStar5.0 software. All sequence files were converted into merged files in Clustal format by SeqVerter software. Multiple-sequence alignments of the genomes and individual genes were performed by the ClustalW program embedded in MEGAX software. Information including the virus name, gene accession number, genotype, isolation time and place is summarized in Supplementary Tables S1 and S2.

### Phylogenetic analysis

Phylogenetic trees based on the genome and individual gene sequences were constructed by the maximum likelihood method with the GTR model using MEGAX. The bootstrap support values were determined with 1000 replicates to assess the robustness of individual nodes of the phylogenies.

### Genetic distance

The genome and individual gene sequences of EV-A71 were first grouped by the genotypes/subgenotypes using the Data program (Edit Taxa/Groups) in MEGAX. The mean genetic distances of the genome and individual gene sequences between DL71 and the reference strains were calculated (Table 1) by the DISTANCE program (Compute Within or Between Group Mean Distance) in MEGAX. The mean genetic distances of the genomes and individual genes of EV-A71 strains within genotypes/subgenotypes were also calculated (Supplementary Table S3). The Kimura 2-parameter model with both transition and transversion substitutions was selected for parameter setting. The bootstrap method with 1000 replications was selected as the variance estimation method.

### Recombination analysis

The genome sequence of DL71 was examined for potential recombination events. The recombination events, breakpoints locations and parental strains were detected by the recombination detection program software version 4.101 (RDP4). 7 detection methods in their default mode, RDP, GENECONV, BOOTSCAN, MAXCHI, CHIMAERA, SISCAN and 3SEQ, were utilized to evaluate the potential recombination events between the inputted aligned sequences. Only the recombination events identified by at least 4 methods were considered significant^46^. The recombination events were further validated by the topological discordance of phylogenetic trees generated for each recombinant regions and non-recombinant regions of virus genomes. The potential recombination events were visualized by Similarity Plot (SimPlot) version 3.5.1. Similarity and Bootscan analyses were carried out using the Neighbor-Joining tree model with the Kimura 2-parameter method. The window size was 200 nucleotides moving along the alignment in 20 bp increments. Information on the alignment, including the genome sequences of DL71 and 42 reference strains used in recombination analyses by RDP4 and SimPlot3.5.1 software, is listed in Supplementary Table S4.

### Nucleotide sequence identity

According to the positions of the recombination breakpoints in the genomes of DL71, Fuyang-0805a and Tainnan/5746/98 (Supplementary Table S5), the sequences of the recombination and non-recombination regions were edited by the EditSeq program of DNAStar5.0. After aligning the corresponding sequences by the ClustalW method, the nucleotide identities in different regions of DL71 and its parental strains were calculated using the MegAlign program in DNAStar5.0.

## Supplementary Information

Supplementary Information.

## Data Availability

All data used in our study are public data. The genome and individual gene sequences of EV-A71 and other viruses are downloaded from the GenBank database (http://www.ncbi.nlm.nih.gov/nucleotide/). The datasets generated during the current study are available from the corresponding authors on reasonable request.
