# Supplementary material for: A novel subgenotype C6 Enterovirus A71 originating from the recombination between subgenotypes C4 and C2 strains in mainland China
Source: Sci Rep. 2022 Jan 12;12:593. doi: 10.1038/s41598-021-04604-x (PMC8755819; doi:10.1038/s41598-021-04604-x)
Supplement: Supplementary file 1 — Supplementary Information. [file 41598_2021_4604_MOESM1_ESM.docx]

**《Scientific Reports》**

**A novel subgenotype C6 Enterovirus A71 originating from the recombination between subgenotypes C4 and C2 strains in mainland China**

Yongjuan Liu, Jingyi Zhou, Guangquan Ji, Yupeng Gao, Chunyan Zhang, Ting Zhang, Juan Huo, Wenxue Liang, Jin Yang, Yingying Shi and Shaolin Zhao

Corresponding authors:

Yingying Shi, Department of Immunology, Jianghan University, Hubei, Wuhan 430056, People’s Republic of China, E-mail: shiyingyinga@126.com

Shaolin Zhao, Department of Central Laboratory, the First People’s Hospital of Lianyungang, Jiangsu, Lianyungang 222000, People’s Republic of China, E-mail: ZhaoSL_2021@163.com

**Electronic Supplementary Materials**

**Supplementary Table S1** The information of EV-A71 strains and CVA16 prototype strain used in phylogenetic analysis.

| Year | Country/ Province | Accession Numbers | Strains | Genotype |
| --- | --- | --- | --- | --- |
| 1951 | Finland | U05876 | CVA16 G-10 | NA |
| 1970 | USA | U22521 | BrCr-CA-70 | A |
| 1965 | Netherlands | AB491212 | 5603 | B0 |
| 1966 | Netherlands | AB575912 | 10857 | B0 |
| 1971 | Netherlands | AB575913 | 11977 | B1 |
| 1971 | Netherlands | AB575914 | 17000 | B1 |
| 1987 | USA | U22522 | MS/7423/87 | B2 |
| 1983 | Netherlands | AB575923 | 20233 | B2 |
| 1997 | Malaysia | AM396588 | EV71/SAR/SHA63 | B3 |
| 1997 | Malaysia | DQ341367 | MY821-3-SAR-97 | B3 |
| 1997 | Malaysia | AJ586873 | EV71/9/97/SHA89 | B4 |
| 2000 | Singapore | AF316321 | 5865/sin/000009 | B4 |
| 2003 | Malaysia | DQ341363 | S19841-SAR-03 | B5 |
| 2000 | Singapore | DQ341364 | 5511-SIN-00 | B5 |
| 2003 | Norway | DQ452074 | 804/NO/03 | C1 |
| 2000 | Australia | DQ341361 | 1M-AUS-12-00 | C1 |
| 2015 | Germany | KU641501 | 37507/TH/DE 2015 | C1-like |
| 2015 | Germany | KU641502 | 45849/TH/DE 2015 | C1-like |
| 1998 | Taiwan | AF176044 | 1245a-98-tw | C2 |
| 1998 | Taiwan | AF304457 | Tainan/5746/98 | C2 |
| 2008 | Taiwan | HM622391 | 2008-00643 | C2-like |
| 2008 | Taiwan | HM622391 | 2008-00643 | C2-like |
| 2003 | Korea | DQ341356 | 03-KOR-00 | C3 |
| 2000 | South Korea | DQ341355 | 06-KOR-00 | C3 |
| 2005 | Taiwan | EF063152 | E2005125-TW | C5 |
| 2007 | Taiwan | EU527983 | 2007-07364 | C5 |
| 2012 | India | KF906422 | R-80135 | D |
| 2010 | India | KF906420 | V10-4243 | D |
| 2003 | Africa | JN255590 | CAF-NMA-03-8 | E |
| 2003 | Cameroon | JX307649 | C08-146 | E |
| 2004 | Madagascar | HG421068 | MAD-72341-04 | F |
| 2011 | Madagascar | HG421069 | MAD-3126-11 | F |
| 2008 | India | KF906416 | V08-5327 | G |
| 2011 | India | KF906417 | V11-2209 | G |
| 1987 | Hubei | AF135934 | 0667-CHN-87 | C0 |
| 1996 | Shandong | JK326306 | 96200/SD/CHN/96 | C2 |
| 1997 | Heilongjiang | AB115494 | 97-56-CHN-97 | C3 |
| 1998 | Shenzhen | AF302996 | SHZH98 | C4b |
| 2000 | Shanghai | AB115490 | F1-CHN-00 | C4b |
| 2000 | Shanghai | AB115491 | F2-CHN-00 | C4b |
| 2000 | Shanghai | AB115492 | H25-CHN-00 | C4b |
| 2000 | Shanghai | AB115493 | H26-CHN-00 | C4b |
| 2001 | Shenzhen | AY895132 | shzh01-3 | C4b |
| 2001 | Shenzhen | AY895134 | shzh01-4 | C4b |
| 2002 | Shanghai | AY547499 | EV71-SHH02-6 | C4a1 |
| 2002 | Shanghai | AY547500 | EV71-SHH02-17 | C4b |
| 2002 | Shenzhen | AY895130 | shzh02-40 | C4b |
| 2002 | Shenzhen | AY895136 | shzh02-62 | C4b |
| 2003 | Shandong | HM212441 | TS011/SD/CHN/2003 | C4a2 |
| 2003 | Shandong | HM212442 | TS013/SD/CHN/2003 | C4a1 |
| 2003 | Shandong | HM212443 | TS014/SD/CHN/2003 | C4a1 |
| 2003 | Shenzhen | AY465356 | SHZH03 | C4a1 |
| 2003 | Shenzhen | AY895133 | shzh03-105 | C4b |
| 2003 | Shenzhen | AY895135 | shzh03-58 | C4a1 |
| 2003 | Shenzhen | AY895138 | shzh03-106 | C4b |
| 2003 | Sichuan | AY547501 | EV71-CQ03-1 | C4a1 |
| 2003 | Zhejiang | AY905614 | ZJ-CHN-1-03 | C4a2 |
| 2003 | Zhejiang | AY905615 | ZJ-CHN-2-03 | C4a2 |
| 2003 | Zhejiang | AY905616 | ZJ-CHN-3-03 | C4a2 |
| 2003 | Zhejiang | AY905617 | ZJ-CHN-4-03 | C4a2 |
| 2003 | Zhejiang | AY905618 | ZJ-CHN-5-03 | C4a2 |
| 2003 | Zhejiang | AY905619 | ZJ-CHN-6-03 | C4a2 |
| 2004 | Shenzhen | AY895129 | shzh04-J38 | C4b |
| 2004 | Shenzhen | AY895131 | shzh04-J42 | C4b |
| 2004 | Shenzhen | AY895137 | shzh04-J40 | C4b |
| 2004 | Shenzhen | AY895139 | shzh04-2 | C4b |
| 2004 | Shenzhen | AY895140 | shzh04-J39 | C4b |
| 2004 | Shenzhen | AY895141 | shzh04-23 | C4b |
| 2004 | Shenzhen | AY895142 | shzh04-3 | C4b |
| 2004 | Shenzhen | AY895143 | shzh04-20 | C4b |
| 2004 | Shenzhen | AY895144 | shzh04-12 | C4a2 |
| 2004 | Shenzhen | AY895145 | shzh04-38 | C4a2 |
| 2004 | Shenzhen | AY895092 | shzh04-J41 | C4b |
| 2005 | Shandong | GQ253420 | 05273/SD/CHN/2005/EV71 | C4a2 |
| 2005 | Shandong | GQ253421 | 05488/SD/CHN/2005/EV71 | C4a2 |
| 2006 | Anhui | EU697903 | AnHui-HeFei-1 | C4a2 |
| 2006 | Shandong | GQ253422 | 06272/SD/CHN/2006/EV71 | C4a2 |
| 2006 | Shandong | GQ253423 | 06282/SD/CHN/2006/EV71 | C4a2 |
| 2006 | Beijing | HQ129932 | BJ06-SJS06 | C4a2 |
| 2007 | Beijing | EU019910 | BJ4243 | C4a2 |
| 2007 | Beijing | EU024958 | BJ4211 | C4a2 |
| 2007 | Beijing | JF317975 | BJ25 | C4a2 |
| 2007 | Beijing | JF317976 | BJ47 | C4a2 |
| 2007 | Inner Mongolia | EU910861 | 0708T/NM/CHN/07 | C4a2 |
| 2007 | Inner Mongolia | EU910862 | 0709F/NM/CHN/07 | C4a2 |
| 2007 | Inner Mongolia | EU910863 | 0711F/NM/CHN/07 | C4a2 |
| 2007 | Inner Mongolia | EU910864 | 0712F/NM/CHN/07 | C4a2 |
| 2007 | Inner Mongolia | EU910865 | 0715F/NM/CHN/07 | C4a2 |
| 2007 | Inner Mongolia | EU910866 | 0716F/NM/CHN/07 | C4a2 |
| 2007 | Inner Mongolia | EU910867 | 0717F/NM/CHN/07 | C4a2 |
| 2007 | Inner Mongolia | EU910868 | 0718F/NM/CHN/07 | C4a2 |
| 2007 | Inner Mongolia | EU910869 | 0723F/NM/CHN/07 | C4a2 |
| 2007 | Shandong | EU753363 | 518-01F/SD/CHN/07 | C4a2 |
| 2007 | Shandong | EU753364 | 518-02F/SD/CHN/07 | C4a2 |
| 2007 | Shandong | EU753365 | 518-03F/SD/CHN/07 | C4a2 |
| 2007 | Shandong | EU753366 | 519-02F/SD/CHN/07 | C4a2 |
| 2007 | Shandong | EU753367 | 521-03T/SD/CHN/07 | C4a2 |
| 2007 | Shandong | EU753368 | 521-04F/SD/CHN/07 | C4a2 |
| 2007 | Shandong | EU753369 | 521-04T/SD/CHN/07 | C4a2 |
| 2007 | Shandong | EU753370 | 521-05T/SD/CHN/07 | C4a2 |
| 2007 | Shandong | EU753371 | 521-08F/SD/CHN/07 | C4a2 |
| 2007 | Shandong | EU753372 | 521-09F/SD/CHN/07 | C4a2 |
| 2007 | Shandong | EU753373 | 521-14F/SD/CHN/07 | C4a2 |
| 2007 | Shandong | EU753374 | 521-17F/SD/CHN/07 | C4a2 |
| 2007 | Shandong | EU753375 | 521-18S/SD/CHN/07 | C4a2 |
| 2007 | Shandong | EU753376 | 521-19T/SD/CHN/07 | C4a2 |
| 2007 | Shandong | EU753377 | 521-20F/SD/CHN/07 | C4a2 |
| 2007 | Shandong | EU753378 | 521-23F/SD/CHN/07 | C4a2 |
| 2007 | Shandong | EU753379 | 521-25F/SD/CHN/07 | C4a2 |
| 2007 | Shandong | EU753380 | 521-27F/SD/CHN/07 | C4a2 |
| 2007 | Shandong | EU753381 | 522-01T/SD/CHN/07 | C4a2 |
| 2007 | Shandong | EU753382 | 522-02F/SD/CHN/07 | C4a2 |
| 2007 | Shandong | EU753383 | 522-03F/SD/CHN/07 | C4a2 |
| 2007 | Shandong | EU753384 | 522-04T/SD/CHN/07 | C4a2 |
| 2007 | Shandong | EU753385 | 522-07T/SD/CHN/07 | C4a2 |
| 2007 | Shandong | EU753386 | 522-08T/SD/CHN/07 | C4a2 |
| 2007 | Shandong | EU753387 | 522-16F/SD/CHN/07 | C4a2 |
| 2007 | Shandong | EU753388 | 522-17F/SD/CHN/07 | C4a2 |
| 2007 | Shandong | EU753389 | 522-17T/SD/CHN/07 | C4a2 |
| 2007 | Shandong | EU753390 | 522-18T/SD/CHN/07 | C4a2 |
| 2007 | Shandong | EU753391 | 522-19F/SD/CHN/07 | C4a2 |
| 2007 | Shandong | EU753392 | 522-21F/SD/CHN/07 | C4a2 |
| 2007 | Shandong | EU753393 | 522-23F/SD/CHN/07 | C4a2 |
| 2007 | Shandong | EU753394 | 522-24F/SD/CHN/07 | C4a2 |
| 2007 | Shandong | EU753395 | 523-03F/SD/CHN/07 | C4a2 |
| 2007 | Shandong | EU753396 | 523-04F/SD/CHN/07 | C4a2 |
| 2007 | Shandong | EU753397 | 523-05T/SD/CHN/07 | C4a2 |
| 2007 | Shandong | EU753398 | 523-07T/SD/CHN/07 | C4a2 |
| 2007 | Shandong | EU753399 | 523-10F/SD/CHN/07 | C4a2 |
| 2007 | Shandong | EU753400 | 523-10T/SD/CHN/07 | C4a2 |
| 2007 | Shandong | EU753401 | 523-11F/SD/CHN/07 | C4a2 |
| 2007 | Shandong | EU753402 | 523-11T/SD/CHN/07 | C4a2 |
| 2007 | Shandong | EU753403 | 523-13T/SD/CHN/07 | C4a2 |
| 2007 | Shandong | EU753404 | 523-14T/SD/CHN/07 | C4a2 |
| 2007 | Shandong | EU753405 | 523-15F/SD/CHN/07 | C4a2 |
| 2007 | Shandong | EU753406 | 523-15T/SD/CHN/07 | C4a2 |
| 2007 | Shandong | EU753407 | TC03F/SD/CHN/07 | C4a2 |
| 2007 | Shandong | EU753408 | TC06T/SD/CHN/07 | C4a2 |
| 2007 | Shandong | EU753409 | TC08F/SD/CHN/07 | C4a2 |
| 2007 | Shandong | EU753410 | TC09F/SD/CHN/07 | C4a2 |
| 2007 | Shandong | EU753411 | TC14F/SD/CHN/07 | C4a2 |
| 2007 | Shandong | EU753412 | TC14T/SD/CHN/07 | C4a2 |
| 2007 | Shandong | EU753413 | TC16T/SD/CHN/07 | C4a2 |
| 2007 | Shandong | EU753414 | TC20F/SD/CHN/07 | C4a2 |
| 2007 | Shandong | EU753415 | TC20T/SD/CHN/07 | C4a2 |
| 2007 | Shandong | EU753416 | TC22F/SD/CHN/07 | C4a2 |
| 2007 | Shandong | EU753417 | TC23F/SD/CHN/07 | C4a2 |
| 2007 | Shandong | EU753418 | TC24F/SD/CHN/07 | C4a2 |
| 2008 | Anhui | EU703812 | EV71/Fuyang.Anhui.P.R.C/17.08/1 | C4a2 |
| 2008 | Anhui | EU703813 | EV71/Fuyang.Anhui.P.R.C/17.08/2 | C4a2 |
| 2008 | Anhui | EU703814 | EV71/Fuyang.Anhui.P.R.C/17.08/3 | C4a2 |
| 2008 | Anhui | EU812515 | FY23 | C4a2 |
| 2008 | Anhui | EU913466 | Fuyang22 | C4a2 |
| 2008 | Anhui | EU913467 | Fuyang5 | C4a2 |
| 2008 | Anhui | EU913468 | Fuyang26 | C4a2 |
| 2008 | Anhui | EU913469 | Fuyang44 | C4a2 |
| 2008 | Anhui | EU913470 | Fuyang31 | C4a2 |
| 2008 | Anhui | EU913471 | Fuyang49 | C4a2 |
| 2008 | Anhui | FJ439769 | Fuyang-0805 | C4a2 |
| 2008 | Anhui | FJ765416 | 542-Anhui-08 | C4a2 |
| 2008 | Anhui | FJ765417 | 549-Anhui-08 | C4a2 |
| 2008 | Anhui | FJ765418 | 552-Anhui-08 | C4a2 |
| 2008 | Anhui | FJ765419 | 559-Anhui-08 | C4a2 |
| 2008 | Anhui | FJ765420 | 562-Anhui-08 | C4a2 |
| 2008 | Anhui | FJ765421 | 566-Anhui-08 | C4a2 |
| 2008 | Anhui | GQ117124 | 001-Luan(CHN)-08 | A |
| 2008 | Anhui | GQ117125 | 1401-Luan(CHN)-08 | A |
| 2008 | Luan | GQ117126 | 1404-Luan(CHN)-08 | A |
| 2008 | Luan | GQ117127 | 1901-Luan(CHN)-08 | A |
| 2008 | Luan | GQ117128 | 1906-Luan(CHN)-08 | A |
| 2008 | Anhui | GQ121417 | EV71/Fuyang.Anhui.CHN/17.08/10 | C4a2 |
| 2008 | Anhui | GQ121418 | EV71/Fuyang.Anhui.CHN/17.08/5 | C4a2 |
| 2008 | Anhui | GQ121419 | EV71/Fuyang.Anhui.CHN/17.08/6 | C4a2 |
| 2008 | Anhui | GQ121420 | EV71/Fuyang.Anhui.CHN/17.08/7 | C4a2 |
| 2008 | Anhui | GQ121421 | EV71/Fuyang.Anhui.CHN/17.08/8 | C4a2 |
| 2008 | Anhui | GQ121422 | EV71/Fuyang.Anhui.CHN/17.08/9 | C4a2 |
| 2008 | Anhui | GQ121423 | EV71/Fuyang.Anhui.CHN/17.08/4 | C4a2 |
| 2008 | Anhui | GQ121424 | EV71/Fuyang.Anhui.CHN/19.08/7 | C4a2 |
| 2008 | Anhui | GQ121425 | EV71/Fuyang.Anhui.CHN/17.08/11 | C4a2 |
| 2008 | Anhui | GQ121426 | EV71/Fuyang.Anhui.CHN/17.08/12 | C4a2 |
| 2008 | Anhui | GQ121427 | EV71/Fuyang.Anhui.CHN/18.08/1 | C4a2 |
| 2008 | Anhui | GQ121428 | EV71/Fuyang.Anhui.CHN/17.08/13 | C4a2 |
| 2008 | Anhui | GQ121430 | EV71/Fuyang.Anhui.CHN/17.08/25 | C4a2 |
| 2008 | Anhui | GQ121431 | EV71/Fuyang.Anhui.CHN/18.08/3 | C4a2 |
| 2008 | Anhui | GQ121432 | EV71/Fuyang.Anhui.CHN/17.08/26 | C4a2 |
| 2008 | Anhui | GQ121433 | EV71/Fuyang.Anhui.CHN/19.08/1 | C4a2 |
| 2008 | Anhui | GQ121434 | EV71/Fuyang.Anhui.CHN/17.08/16 | C4a2 |
| 2008 | Anhui | GQ121435 | EV71/Fuyang.Anhui.CHN/19.08/2 | C4a2 |
| 2008 | Anhui | GQ121436 | EV71/Fuyang.Anhui.CHN/18.08/8 | C4a2 |
| 2008 | Anhui | GQ121437 | EV71/Fuyang.Anhui.CHN/19.08/3 | C4a2 |
| 2008 | Anhui | GQ121438 | EV71/Fuyang.Anhui.CHN/17.08/17 | C4a2 |
| 2008 | Anhui | GQ121439 | EV71/Fuyang.Anhui.CHN/19.08/4 | C4a2 |
| 2008 | Anhui | GQ121440 | EV71/Fuyang.Anhui.CHN/19.08/5 | C4a2 |
| 2008 | Anhui | GQ121441 | EV71/Fuyang.Anhui.CHN/19.08/6 | C4a2 |
| 2008 | Anhui | GU350629 | Fuyang-0805a | C4b |
| 2008 | Anhui | GU459070 | FY23-K12 | C4a2 |
| 2008 | Anhui | GU459071 | FY23-K14 | C4a2 |
| 2008 | Anhui | GU198367 | FY08-C30-P2 | C4a2 |
| 2008 | Anhui | GU198368 | C1/FY08-C30-P9 | C4a2 |
| 2008 | Anhui | GU198369 | C1/FY08-C30-P11 | C4a2 |
| 2008 | Anhui | GU198370 | C2/FY08-C30 | C4a2 |
| 2008 | Anhui | GU198371 | FY08-C30-P14 | C4a2 |
| 2008 | Anhui | GU198372 | FY08-C30 | C4a2 |
| 2008 | Anhui | GU198373 | FY08-C30-P3 | C4a2 |
| 2008 | Anhui | GU198374 | FY08-C30-P4 | C4a2 |
| 2008 | Anhui | GU198375 | C1/FY08-C30-P7 | C4a2 |
| 2008 | Anhui | GU198376 | C1/FY08-C30-P19 | C4a2 |
| 2008 | Anhui | HQ882182 | FY0805 | C4a2 |
| 2008 | Anhui | HM064456 | Fuyang573 | C4b |
| 2008 | Anhui | HM212455 | FY08-7/AH/CHN/2008 | C4a2 |
| 2008 | Anhui | HM212456 | FY08-4/AH/CHN/2008 | C4a2 |
| 2008 | Anhui | HM212457 | FY08-9/AH/CHN/2008 | C4a2 |
| 2008 | Anhui | HM212458 | FY08-16/AH/CHN/2008 | C4a2 |
| 2008 | Anhui | HQ188292 | Fuyang-0805a | C4b |
| 2008 | Anhui | HQ611148 | AH08/06 | C4a2 |
| 2008 | Anhui | HQ694983 | BZ200805 | C4b |
| 2008 | Anhui | HQ694984 | Fuyang200805 | C4a2 |
| 2008 | Anhui | HQ694985 | MZ2008 | C4a2 |
| 2008 | Anhui | HQ328791 | EV71/Fuyang. anhui. CHN/20-1 | C4a2 |
| 2008 | Anhui | HQ328792 | EV71/Fuyang. anhui. CHN/20-1 | C4a2 |
| 2008 | Anhui | HQ328793 | EV71/Fuyang. anhui. CHN/c30 | C4a2 |
| 2008 | Beijing | FJ469152 | CY6/BJ/CHN/2008 | C4a2 |
| 2008 | Beijing | FJ469153 | CY11/BJ/CHN/2008 | C4a2 |
| 2008 | Beijing | FJ469154 | CY15/BJ/CHN/2008 | C4a2 |
| 2008 | Beijing | FJ469155 | CY17/BJ/CHN/2008 | C4a2 |
| 2008 | Beijing | FJ469156 | CY20/BJ/CHN/2008 | C4a2 |
| 2008 | Beijing | FJ469157 | CY21/BJ/CHN/2008 | C4a2 |
| 2008 | Beijing | FJ469158 | CY28/BJ/CHN/2008 | C4a2 |
| 2008 | Beijing | FJ469159 | Cy29/BJ/CHN/2008 | C4a2 |
| 2008 | Beijing | FJ469160 | CY43/BJ/CHN/2008 | C4a2 |
| 2008 | Beijing | FJ469161 | CY44/BJ/CHN/2008 | C4a2 |
| 2008 | Beijing | FJ606447 | BJ08-Z004-3 | C4a2 |
| 2008 | Beijing | FJ606448 | BJ08-Z011-4 | C4a2 |
| 2008 | Beijing | FJ606449 | BJ08-Z020-1 | C4a2 |
| 2008 | Beijing | FJ606450 | BJ08-Z025-5 | C4a2 |
| 2008 | Beijing | FJ765422 | BJCDC01-08 | C4a2 |
| 2008 | Beijing | FJ765423 | BJCDC03-08 | C4a2 |
| 2008 | Beijing | FJ765424 | CC01-08 | C4a2 |
| 2008 | Beijing | FJ828519 | BJ08 | C4a2 |
| 2008 | Beijing | HM053669 | BJ293 | C4a2 |
| 2008 | Beijing | JF317977 | BJ65 | C4a2 |
| 2008 | Beijing | JF317978 | BJ67 | C4a2 |
| 2008 | Beijing | JF317979 | BJ97 | C4a2 |
| 2008 | Beijing | JF317980 | BJ108 | C4a2 |
| 2008 | Beijing | JF317981 | BJ110B | C4a2 |
| 2008 | Beijing | JF317982 | BJ110Y | C4a2 |
| 2008 | Fujian | JF420547 | 03382 | C4a2 |
| 2008 | Fujian | JF420585 | NT5 | C4a2 |
| 2008 | Fujian | JN646108 | R22T | C4a2 |
| 2008 | Fujian | JN646109 | XM1985 | C4a2 |
| 2008 | Guangdong | FJ194964 | EV71/GDFS/3/2008 | C4a2 |
| 2008 | Guangdong | FJ194965 | EV71/GDSG/17/2008 | C4a2 |
| 2008 | Shenzhen | FJ607334 | 1/SHENZHEN/08/China/HFMD/2008 | C4a2 |
| 2008 | Shenzhen | FJ607335 | 4/SHENZHEN/08/China/HFMD/2008 | C4a2 |
| 2008 | Shenzhen | FJ607336 | 28/SHENZHEN/08/China/HFMD/2008 | C4a2 |
| 2008 | Shenzhen | FJ607337 | 121/SHENZHEN/08/China/HFMD Fatal/2008 | C4a2 |
| 2008 | Shenzhen | FJ607338 | 605/SHENZHEN/08/China/HFMD Severe/2008 | C4a2 |
| 2008 | Shenzhen | FJ765428 | SZ-A-08 | C4a2 |
| 2008 | Shenzhen | FJ765429 | SZ-C-08 | C4a2 |
| 2008 | Shenzhen | FJ765430 | SZ-H-08 | C4a2 |
| 2008 | Guangdong | FJ360544 | GZ-08-01 | C4a2 |
| 2008 | Guangdong | FJ360545 | GZ-08-02 | C4a2 |
| 2008 | Guangdong | FJ360546 | GZ-08-03 | C4a2 |
| 2008 | Guangdong | GU190169 | 1/GZ/CHN/2008 | C4a2 |
| 2008 | Guangdong | GU190170 | 122/GZ/CHN/2008 | C4a2 |
| 2008 | Guangdong | GU190171 | 129/GZ/CHN/2008 | C4a2 |
| 2008 | Guangdong | GU190172 | 130/GZ/CHN/2008 | C4a2 |
| 2008 | Guangdong | GU190173 | 133/GZ/CHN/2008 | C4a2 |
| 2008 | Guangdong | GU190174 | 136/GZ/CHN/2008 | C4a2 |
| 2008 | Guangdong | GU190175 | 142/GZ/CHN/2008 | C4a2 |
| 2008 | Guangdong | GU190176 | 145/GZ/CHN/2008 | C4a2 |
| 2008 | Guangdong | GU190177 | 152/GZ/CHN/2008 | C4a2 |
| 2008 | Guangdong | GU190178 | 168/GZ/CHN/2008 | C4a2 |
| 2008 | Guangdong | HM037792 | GD08-EV71-003 | C4a2 |
| 2008 | Guangdong | HM037793 | GD08-EV71-039 | C4a2 |
| 2008 | Guangdong | HM037794 | GD08-EV71-135 | C4a2 |
| 2008 | Guangdong | HM037795 | GD08-EV71-149 | C4a2 |
| 2008 | Guangdong | HM037796 | GD08-EV71-166 | C4a2 |
| 2008 | Guangdong | HM037797 | GD08-EV71-194 | C4a2 |
| 2008 | Guangdong | HM037798 | GD08-EV71-205 | C4a2 |
| 2008 | Guangdong | HM037799 | GD08-EV71-274 | C4a2 |
| 2008 | Guangdong | HM037800 | GD08-EV71-335 | C4a2 |
| 2008 | Guangdong | HM037801 | GD08-EV71-381 | C4a2 |
| 2008 | Guangdong | HM037802 | GD08-EV71-495 | C4a2 |
| 2008 | Guangdong | HM037803 | GD08-EV71-523 | C4a2 |
| 2008 | Guangdong | HM037804 | GD08-EV71-573 | C4a2 |
| 2008 | Guangdong | HM037805 | GD08-EV71-583 | C4a2 |
| 2008 | Guangdong | HM037806 | GD08-EV71-614 | C4a2 |
| 2008 | Guangdong | HM037807 | GD08-EV71-641 | C4a2 |
| 2008 | Guangdong | HM037808 | GD08-EV71-650 | C4a2 |
| 2008 | Guangdong | HQ456309 | GZ08-831 | C4a2 |
| 2008 | Guangdong | HQ456310 | EV71/Guangzhou/530/2008 | C4a2 |
| 2008 | Guangdong | HQ456311 | GZ08-522 | C4a2 |
| 2008 | Guangdong | HQ456312 | EV71/Guangzhou/520/2008 | C4a2 |
| 2008 | Guangxi | GQ892830 | GX/LZ 08-04/08/CHN | C4a2 |
| 2008 | Hangzhou | JN205801 | Hangzhou-3-2008 | C4a2 |
| 2008 | Hangzhou | JN205802 | Hangzhou-7-2008 | C4a2 |
| 2008 | Hangzhou | JN205803 | Hangzhou-9-2008 | C4a2 |
| 2008 | Hangzhou | JN205804 | Hangzhou-8-2008 | C4a2 |
| 2008 | Hangzhou | JN205806 | Hangzhou-23-2011 | C4a2 |
| 2008 | Hangzhou | JN205807 | Hangzhou-40-2011 | C4a2 |
| 2008 | Hangzhou | JN205808 | Hangzhou-41-2011 | C4a2 |
| 2008 | Hebei | HM212444 | 9/HeB/CHN/2008 | C4a2 |
| 2008 | Hebei | HM212445 | 11/HeB/CHN/2008 | C4a2 |
| 2008 | Hebei | HM212446 | 20/HeB/CHN/2008 | C4a2 |
| 2008 | Hebei | HM212447 | 24/HeB/CHN/2008 | C4a2 |
| 2008 | Hebei | HM212448 | 40/HeB/CHN/2008 | C4a2 |
| 2008 | Hebei | HM212449 | 48/HeB/CHN/2008 | C4a2 |
| 2008 | Hebei | HM212450 | 50/HeB/CHN/2008 | C4a2 |
| 2008 | Henan | GQ121133 | HN08-HLF2 | C4a2 |
| 2008 | Henan | GQ121134 | HN08-HLF3 | C4a2 |
| 2008 | Henan | GU366191 | Henan10-08-China | C4a2 |
| 2008 | Henan | HM038010 | HN08-HLF4 | C4a2 |
| 2008 | Henan | HM038011 | HN08-HLF5 | C4a2 |
| 2008 | Henan | HM038012 | HN08-HLF8 | C4a2 |
| 2008 | Henan | HM038013 | HN08-HLF14 | C4a2 |
| 2008 | Henan | HM038014 | HN08-HLF21 | C4a2 |
| 2008 | Henan | HM038015 | HN08-HLF23 | C4a2 |
| 2008 | Henan | HM212451 | 08-8/HeN/CHN/2008 | C4a2 |
| 2008 | Wuhan | FJ765431 | WH-2-08 | C4a2 |
| 2008 | Wuhan | FJ765432 | WH-3-08 | C4a2 |
| 2008 | Wuhan | FJ765433 | WH-4-08 | C4a2 |
| 2008 | Wuhan | FJ765434 | WH-8-08 | C4a2 |
| 2008 | Wuhan | FJ765435 | WH-9-08 | C4a2 |
| 2008 | Jilin | HM212452 | 08-2/JL/CHN/2008 | C4a2 |
| 2008 | Jilin | HM212453 | 08-7/JL/CHN/2008 | C4a2 |
| 2008 | Jilin | HM212454 | 08-8/JL/CHN/2008 | C4a2 |
| 2008 | Jiangsu | FJ600325 | EV71/Jiangsu.P.R.C/07.08/10 | C4a2 |
| 2008 | kunming | FJ765425 | kunming24-08 | C4a2 |
| 2008 | kunming | FJ765426 | kunming29-08 | C4a2 |
| 2008 | kunming | FJ765427 | kunming41-08 | C4a2 |
| 2008 | Yunnan | HM212459 | 08-5/YN/CHN/2008 | C4a2 |
| 2008 | Lanzhou | GQ855285 | EV71/Lanzhou01 | C4a2 |
| 2008 | Lanzhou | GQ855286 | EV71/Lanzhou02 | C4a2 |
| 2008 | Lanzhou | GQ855287 | EV71/Lanzhou03 | C4a2 |
| 2008 | Lanzhou | GQ855288 | EV71/Lanzhou04 | C4a2 |
| 2008 | Lanzhou | GQ855289 | EV71/Lanzhou05 | C4a2 |
| 2008 | Lanzhou | GQ855290 | EV71/Lanzhou06 | C4a2 |
| 2008 | Lanzhou | GQ855291 | EV71/Lanzhou07 | C4a2 |
| 2008 | Lanzhou | GQ855292 | EV71/Lanzhou08 | C4a2 |
| 2008 | Lanzhou | GQ855293 | EV71/Lanzhou09 | C4a2 |
| 2008 | Lanzhou | GQ855294 | EV71/Lanzhou10 | C4a2 |
| 2008 | Shandong | GQ253391 | H027F/SD/CHN/2008/EV71 | C4a2 |
| 2008 | Shandong | GQ253392 | H150F/SD/CHN/2008/EV71 | C4a2 |
| 2008 | Shandong | GQ253393 | H362F/SD/CHN/2008/EV71 | C4a2 |
| 2008 | Shandong | GQ253394 | H419F/SD/CHN/2008/EV71 | C4a2 |
| 2008 | Shandong | GQ253395 | H533F/SD/CHN/2008/EV71 | C4a2 |
| 2008 | Shandong | GQ253396 | H553F/SD/CHN/2008/EV71 | C4a2 |
| 2008 | Shandong | GQ253397 | H903F/SD/CHN/2008/EV71 | C4a2 |
| 2008 | Shandong | GQ253398 | H1218F/SD/CHN/2008/EV71 | C4a2 |
| 2008 | Shandong | GQ253399 | H1261F/SD/CHN/2008/EV71 | C4a2 |
| 2008 | Shandong | HQ825317 | EV71/JN200804 | C4a2 |
| 2008 | Shandong | JF913464 | EV71/JN200803 | C4a2 |
| 2008 | Shanghai | HM579938 | SHAPHC3H/SH/CHN/08 | C4a2 |
| 2008 | Shanghai | HM579939 | SHAPHC11H/SH/CHN/08 | C4a2 |
| 2008 | Shanghai | HM579940 | SHAPHC34H/SH/CHN/08 | C4a2 |
| 2008 | Shanghai | HM579941 | SHAPHC42T/SH/CHN/08 | C4a2 |
| 2008 | Shanghai | HM579942 | SHAPHC44T/SH/CHN/08 | C4a2 |
| 2008 | Shanghai | HM579943 | SHAPHC59T/SH/CHN/08 | C4a2 |
| 2008 | Shanghai | HM579944 | SHAPHC61T/SH/CHN/08 | C4a2 |
| 2008 | Shanghai | HM579945 | SHAPHC154F/SH/CHN/08 | C4a2 |
| 2008 | Shanghai | JF918554 | SHAPHC122F/SH/CHN/08 | C4a2 |
| 2008 | Shanghai | JF918555 | SHAPHC49T/SH/CHN/08 | C4a2 |
| 2008 | Shanghai | JF918556 | SHAPHC79T/SH/CHN/08 | C4a2 |
| 2008 | Xi’an | HM003207 | 87-2008 Xi'an Shaanxi | C4a2 |
| 2008 | Zhejiang | EU864507 | EV71/Zhejiang08 | C4a2 |
| 2008 | Zhejiang | FJ158600 | DTID/ZJU-62 | C4a2 |
| 2008 | Zhejiang | FJ158601 | DTID/ZJU-74 | C4a2 |
| 2008 | Zhejiang | FJ594956 | ZJ001 | C4a2 |
| 2008 | Zhejiang | HQ400942 | HZ08 | C4a2 |
| 2008 | Zhuhai | EU999170 | Zhuhai-JC467 | C4a2 |
| 2008 | Zhuhai | EU999171 | Zhuhai-JC455 | C4a2 |
| 2008 | Zhuhai | EU999172 | Zhuhai-JC498 | C4a2 |
| 2008 | Zhuhai | EU999173 | Zhuhai-213 | C4a2 |
| 2008 | Zhuhai | EU999174 | Zhuhai-171 | C4a2 |
| 2008 | Zhuhai | EU999175 | Zhuhai-164 | C4a2 |
| 2008 | Zhuhai | EU999176 | Zhuhai-152 | C4a2 |
| 2008 | Zhuhai | EU999177 | Xinhui-7 | C4a2 |
| 2008 | Zhuhai | EU999178 | Xinhui-8 | C4a2 |
| 2008 | Zhuhai | EU999179 | Xinhui-9 | C4a2 |
| 2008 | Ningxia | HM212460 | 082/NX/CHN/2008 | C4a2 |
| 2008 | Ningxia | HM212461 | 117/NX/CHN/2008 | C4a2 |
| 2008 | Ningxia | HM212462 | 174/NX/CHN/2008 | C4a2 |
| 2008 | Ningxia | HM212463 | 201/NX/CHN/2008 | C4a2 |
| 2008 | Ningxia | HM212464 | 207/NX/CHN/2008 | C4a2 |
| 2009 | Anhui | GQ994988 | Anhui1-09-China | C4a2 |
| 2009 | Anhnui | HQ694982 | JK2009 | C4a2 |
| 2009 | Beijing | HM002484 | BJ67 | C4a2 |
| 2009 | Beijing | HM002485 | BJ97 | C4a2 |
| 2009 | Beijing | HM002486 | BJ110 | C4a2 |
| 2009 | Beijing | HM002487 | BJ303 | C4a2 |
| 2009 | Beijing | HM002488 | BJ366 | C4a2 |
| 2009 | Beijing | HM002489 | BJ398 | C4a2 |
| 2009 | Beijing | HM053670 | BJ393 | C4a2 |
| 2009 | Beijing | HM053671 | BJ462 | C4a2 |
| 2009 | Beijing | JF317983 | BJ366 | C4a2 |
| 2009 | Beijing | JF317984 | BJ374 | C4a2 |
| 2009 | Beijing | JF317985 | BJ391 | C4a2 |
| 2009 | Beijing | JF317986 | BJ398 | C4a2 |
| 2009 | Beijing | JQ410994 | EV71/CMU1-2/BJ/CHN/2009 | A |
| 2009 | Beijing | JQ410995 | EV71/CMU3-1/BJ/CHN/2009 | A |
| 2009 | Beijing | JQ411000 | EV71/CMU21-2/BJ/CHN/2009 | A |
| 2009 | Beijing | JQ411003 | EV71/CMU28-2/BJ/CHN/2009 | A |
| 2009 | Beijing | JQ411006 | EV71/CMU33-1/BJ/CHN/2009 | A |
| 2009 | Beijing | JQ411008 | EV71/CMU46-1/BJ/CHN/2009 | A |
| 2009 | Beijing | JQ411009 | EV71/CMU47-1/BJ/CHN/2009 | A |
| 2009 | Beijing | JQ411010 | EV71/CMU48-1/BJ/CHN/2009 | A |
| 2009 | Guangdong | GQ487667 | ZH-ETC335/GD/CHN/09 | C4a2 |
| 2009 | Guangdong | GQ487669 | ZH-ETC278/GD/CHN/09 | C4a2 |
| 2009 | Guangdong | GQ487672 | ZH-ETC153/GD/CHN/09 | C4a2 |
| 2009 | Guangdong | GQ487673 | ZH-ETC368/GD/CHN/09 | C4a2 |
| 2009 | Guangdong | GQ487687 | ZH-ETC346/GD/CHN/09 | C4a2 |
| 2009 | Guangdong | GQ487688 | ZH-JC334/GD/CHN/09 | C4a2 |
| 2009 | Guangdong | JF519696 | 480/GD/China/2009 | C4a2 |
| 2009 | Guangdong | JF519698 | 419/GD/China/2009 | C4a2 |
| 2009 | Guangdong | JF519700 | 394/GD/China/2009 | C4a2 |
| 2009 | Guangdong | JF519702 | 265/GD/China/2009 | C4a2 |
| 2009 | Guangdong | GQ487666 | ZH-ETC377/GD/CHN/09 | C4a2 |
| 2009 | Guangdong | GQ487668 | ZH-JC220/GD/CHN/09 | C4a2 |
| 2009 | Guangdong | GQ487670 | ZH-JC215/GD/CHN/09 | C4a2 |
| 2009 | Guangdong | GQ487671 | ZH-ETC222/GD/CHN/09 | C4a2 |
| 2009 | Guangdong | GQ487674 | ZH-ETC352/GD/CHN/09 | C4a2 |
| 2009 | Guangdong | GQ487675 | ZH-JC344/GD/CHN/09 | C4a2 |
| 2009 | Guangdong | GQ487676 | ZH-JC431/GD/CHN/09 | C4a2 |
| 2009 | Guangdong | GQ487677 | ZH-JC414/GD/CHN/09 | C4a2 |
| 2009 | Guangdong | GQ487679 | ZH-ETC321/GD/CHN/09 | C4a2 |
| 2009 | Guangdong | GQ487681 | ZH-ETC275/GD/CHN/09 | C4a2 |
| 2009 | Guangdong | GQ487682 | ZH-JC209/GD/CHN/09 | C4a2 |
| 2009 | Guangdong | GQ487683 | ZH-ETC180/GD/CHN/09 | C4a2 |
| 2009 | Guangdong | GQ487684 | ZH-ETC110/GD/CHN/09 | C4a2 |
| 2009 | Guangdong | GQ487685 | ZH-ETC367/GD/CHN/09 | C4a2 |
| 2009 | Guangdong | GQ487686 | ZH-ETC385/GD/CHN/09 | C4a2 |
| 2009 | Guangdong | GQ487689 | ZH-JC426/GD/CHN/09 | C4a2 |
| 2009 | Guangdong | JF519697 | 467/GD/China/2009 | C4a2 |
| 2009 | Guangdong | JF519699 | 395/GD/China/2009 | C4a2 |
| 2009 | Guangdong | JF519701 | 374/GD/China/2009 | C4a2 |
| 2009 | Guangdong | JF519703 | 070/GD/China/2009 | C4a2 |
| 2009 | Guangdong | JF519704 | 067/GD/China/2009 | C4a2 |
| 2009 | Guangdong | JF519705 | 063/GD/China/2009 | C4a2 |
| 2009 | Guangdong | JF519706 | 061/GD/China/2009 | C4a2 |
| 2009 | Guangdong | JF519707 | 060/GD/China/2009 | C4a2 |
| 2009 | Guangdong | GQ487678 | ZH-ETC351/GD/CHN/09 | C4a2 |
| 2009 | Guangdong | JF799986 | Guangdong 2009 | C4a2 |
| 2009 | Guangdong | GU190179 | 373/GZ/CHN/2009 | C4a2 |
| 2009 | Guangxi | GU176408 | Nanjing08-1 | C4a2 |
| 2009 | Henan | HM212465 | 09-1/HeN/CHN/2009 | C4a2 |
| 2009 | Henan | HM212466 | 09-17/HeN/CHN/2009 | C4a2 |
| 2009 | Henan | JN835271 | G224-882F/HeN/CHN/2009 | C4a2 |
| 2009 | Henan | JN835272 | G227-885F/HeN/CHN/2009 | C4a2 |
| 2009 | Henan | JN835273 | G252-910F/HeN/CHN/2009 | C4a2 |
| 2009 | Henan | JN835274 | G256-914F/HeN/CHN/2009 | C4a2 |
| 2009 | Henan | JN835275 | G283-922F/HeN/CHN/2009 | C4a2 |
| 2009 | Henan | JN835276 | G306-945F/HeN/CHN/2009 | C4a2 |
| 2009 | Henan | JN835277 | G348-987F/HeN/CHN/2009 | C4a2 |
| 2009 | Henan | JN835278 | G392-1031F/HeN/CHN/2009 | C4a2 |
| 2009 | Henan | JN835279 | G400-1039F/HeN/CHN/2009 | C4a2 |
| 2009 | Henan | JN835280 | G405-1045F/HeN/CHN/2009 | C4a2 |
| 2009 | Henan | JN835281 | G443-1083F/HeN/CHN/2009 | C4a2 |
| 2009 | Henan | JN835282 | G523-1157T/HeN/CHN/2009 | C4a2 |
| 2009 | Henan | JN835283 | G541-1175F/HeN/CHN/2009 | C4a2 |
| 2009 | Henan | JN835284 | M184-1177F/HeN/CHN/2009 | C4a2 |
| 2009 | Henan | GU196833 | Henan1-09-China | C4a2 |
| 2009 | Henan | HQ998852 | EV71/Henan/106/2009 | C4a2 |
| 2009 | Henan | GQ994992 | Henan2-09-China | C4a2 |
| 2009 | Henan | JN256059 | G288-927F/HeN/CHN/2009 | C4a2 |
| 2009 | Henan | JN256060 | G333-972F/HeN/CHN/2009 | C4a2 |
| 2009 | Henan | JN256061 | G398-1037F/HeN/CHN/2009 | C4a2 |
| 2009 | Henan | JN256062 | M183-1176F/HeN/CHN/2009 | C4a2 |
| 2009 | Henan | JN256063 | M186-1179F/HeN/CHN/2009 | C4a2 |
| 2009 | Henan | JN256064 | M188-1181F/HeN/CHN/2009 | C4a2 |
| 2009 | Hubei | HM212436 | 09-20/HuB/CHN/2009 | C4a1 |
| 2009 | Hubei | HM212437 | 09-29H/HuB/CHN/2009 | C4a1 |
| 2009 | Hubei | HM212438 | 09-32H/HuB/CHN/2009 | C4a1 |
| 2009 | Hubei | HM212439 | 09-33T/HuB/CHN/2009 | C4a2 |
| 2009 | Hubei | HM212440 | 09-40H/HuB/CHN/2009 | C4a1 |
| 2009 | Hubei | JN230523 | Xiangyang-Hubei-09 | C4b |
| 2009 | Hubei | GU434678 | EV71-Hubei-09-China | A |
| 2009 | Jiangsu | GU353081 | Changzhou/JS10/CHN09 | C4a2 |
| 2009 | Jiangsu | GU353083 | Lianyungang/JS13/CHN09 | C4a2 |
| 2009 | Jiangsu | GU353085 | Nantong/JS15/CHN09 | C4a2 |
| 2009 | Jiangsu | GU353095 | Yangzhou/JS36/CHN09 | C4a2 |
| 2009 | Jiangsu | GU353080 | Nanjing/JS06/CHN09 | C4a2 |
| 2009 | Jiangsu | GU353082 | Lianyungang/JS12/CHN09 | C4a2 |
| 2009 | Jiangsu | GU353084 | Lianyungang/JS14/CHN09 | C4a2 |
| 2009 | Jiangsu | GU353086 | Nantong/JS16/CHN09 | C4a2 |
| 2009 | Jiangsu | GU353087 | Taizhou/JS18/CHN09 | C4a2 |
| 2009 | Jiangsu | GU353088 | Suqian/JS22/CHN09 | C4a2 |
| 2009 | Jiangsu | GU353089 | Nantong/JS23/CHN09 | C4a2 |
| 2009 | Jiangsu | GU353090 | Lianyungang/JS24/CHN09 | C4a2 |
| 2009 | Jiangsu | GU353091 | Nantong/JS29/CHN09 | C4a2 |
| 2009 | Jiangsu | GU353092 | Suzhou/JS30/CHN09 | C4a2 |
| 2009 | Jiangsu | GU353093 | Suqian/JS34/CHN09 | C4a2 |
| 2009 | Jiangsu | GU353094 | Yangzhou/JS35/CHN09 | C4a2 |
| 2009 | Jiangsu | GU353096 | Zhenjiang/JS41/CHN09 | C4a2 |
| 2009 | Jiangsu | GU353097 | Zhenjiang/JS42/CHN09 | C4a2 |
| 2009 | Jiangsu | GU353098 | Zhenjiang/JS43/CHN09 | C4a2 |
| 2009 | Jiangsu | GU353079 | Nanjing/JS05/CHN09 | C4a2 |
| 2009 | Jiangsu | GU353101 | Zhenjiang/JS46/CHN09 | C4a2 |
| 2009 | Jiangsu | GU353102 | Zhenjiang/JS51/CHN09 | C4a2 |
| 2009 | Jiangsu | GU353106 | Zhenjiang/JS60/CHN09 | C4a2 |
| 2009 | Jiangsu | GU353099 | Zhenjiang/JS44/CHN09 | C4a2 |
| 2009 | Jiangsu | GU353100 | Zhenjiang/JS45/CHN09 | C4a2 |
| 2009 | Jiangsu | GU353103 | Zhenjiang/JS56/CHN09 | C4a2 |
| 2009 | Jiangsu | GU353104 | Zhenjiang/JS57/CHN09 | C4a2 |
| 2009 | Jiangsu | GU353105 | Zhenjiang/JS59/CHN09 | C4a2 |
| 2009 | Shandong | GQ253405 | HZ121F/SD/CHN/2009/EV71 | C4a2 |
| 2009 | Shandong | GQ253407 | JN93F/SD/CHN/2009/EV71 | C4a2 |
| 2009 | Shandong | GQ253410 | LC0028F/SD/CHN/2009/EV71 | C4a2 |
| 2009 | Shandong | GQ253412 | LW0009F/SD/CHN/2009/EV71 | C4a2 |
| 2009 | Shandong | GQ253415 | LY0009F/SD/CHN/2009/EV71 | C4a2 |
| 2009 | Shandong | GQ253417 | RZ0010F/SD/CHN/2009/EV71 | C4a2 |
| 2009 | Shandong | GQ253400 | HZ0137F/SD/CHN/2009/EV71 | C4a2 |
| 2009 | Shandong | GQ253401 | HZ150F/SD/CHN/2009/EV71 | C4a2 |
| 2009 | Shandong | GQ253402 | HZ152F/SD/CHN/2009/EV71 | C4a2 |
| 2009 | Shandong | GQ253403 | HZ0011F/SD/CHN/2009/EV71 | C4a2 |
| 2009 | Shandong | GQ253404 | HZ0048F/SD/CHN/2009/EV71 | C4a2 |
| 2009 | Shandong | GQ253406 | JN92F/SD/CHN/2009/EV71 | C4a2 |
| 2009 | Shandong | GQ253408 | JN94F/SD/CHN/2009/EV71 | C4a2 |
| 2009 | Shandong | GQ253409 | LC0013H/SD/CHN/2009/EV71 | C4a2 |
| 2009 | Shandong | GQ253411 | LW0004F/SD/CHN/2009/EV71 | C4a2 |
| 2009 | Shandong | GQ253413 | LW0011F/SD/CHN/2009/EV71 | C4a2 |
| 2009 | Shandong | GQ253414 | LY0004F/SD/CHN/2009/EV71 | C4a2 |
| 2009 | Shandong | GQ253416 | RZ0008F/SD/CHN/2009/EV71 | C4a2 |
| 2009 | Shandong | GQ253418 | ZB0001F/SD/CHN/2009/EV71 | C4a2 |
| 2009 | Shandong | GQ253419 | ZB0002F/SD/CHN/2009/EV71 | C4a2 |
| 2009 | Shanghai | HQ667783 | SH/CHN/2009 | C4a2 |
| 2009 | Shanghai | HQ667784 | SH/CHN/2009 | C4a2 |
| 2009 | Shanghai | HQ667785 | SH/CHN/2009 | C4a2 |
| 2009 | Shanghai | HQ667787 | SH/CHN/2009 | C4a2 |
| 2009 | Shanghai | JF918557 | SHAPHC371T/SH/CHN/09 | C4a2 |
| 2009 | Shanghai | JF918558 | SHAPHC398F/SH/CHN/09 | C4a2 |
| 2009 | Shanghai | JF918559 | SHAPHC382T/SH/CHN/09 | C4a2 |
| 2009 | Shanghai | JF918560 | SHAPHC315T/SH/CHN/09 | C4a2 |
| 2009 | Shanghai | JF918561 | SHAPHC332F/SH/CHN/09 | C4a2 |
| 2009 | Shanghai | JF918562 | SHAPHC363T/SH/CHN/09 | C4a2 |
| 2009 | Shanghai | HQ891923 | Shanghai 27-2009 | C4a2 |
| 2009 | Shanghai | HQ891925 | Shanghai 36-2009 | C4a2 |
| 2009 | Shanghai | HQ891926 | Shanghai 51-2009 | C4a2 |
| 2009 | Shanghai | HQ891927 | Shanghai 64-2009 | C4a2 |
| 2009 | Shanghai | HQ891928 | Shanghai 117-2009 | C4a2 |
| 2009 | Shanghai | HQ891929 | Shanghai 118-2009 | C4a2 |
| 2009 | Shanghai | HQ891924 | Shanghai 28-2009 | C4a2 |
| 2009 | Shanghai | FJ713137 | Shanghai 036-2009 | C4a2 |
| 2009 | Shanghai | HQ667786 | SH/CHN/2009 | C4a2 |
| 2009 | Ximen | JN964686 | EV71/Xiamen/2009 | B5 |
| 2009 | Yunnan | JF505392 | KM23/09 | C4a2 |
| 2009 | Yunnan | JF505389 | KM9/09 | C4a2 |
| 2009 | Yunnan | JF505390 | KM11/09 | C4a2 |
| 2009 | Yunnan | JF505391 | KM16/09 | C4a2 |
| 2009 | Yunnan | JN408335 | 1-YN-CHN-2009 | C4a2 |
| 2009 | Yunnan | JN408336 | 2-YN-CHN-2009 | C4a2 |
| 2009 | Yunnan | JN408337 | 3-YN-CHN-2009 | C4a2 |
| 2009 | Yunnan | JN408338 | 4-YN-CHN-2009 | C4a2 |
| 2009 | Yunnan | JN408339 | 6-YN-CHN-2009 | C4a2 |
| 2009 | Yunnan | JN408340 | 7-YN-CHN-2009 | C4a2 |
| 2009 | Yunnan | JN408341 | 8-YN-CHN-2009 | C4a2 |
| 2009 | Yunnan | JN408342 | 9-YN-CHN-2009 | A |
| 2009 | Yunnan | JN408343 | 10-YN-CHN-2009 | A |
| 2009 | Yunnan | JN408344 | 11-YN-CHN-2009 | C4a2 |
| 2009 | Yunnan | JN408345 | 12-YN-CHN-2009 | C4a2 |
| 2009 | Yunnan | JN408346 | 13-YN-CHN-2009 | C4a2 |
| 2009 | Yunnan | JN408347 | 15-YN-CHN-2009 | C4a2 |
| 2009 | Yunnan | JN408348 | 16-YN-CHN-2009 | C4a2 |
| 2009 | Yunnan | JN408349 | 17-YN-CHN-2009 | C4a2 |
| 2009 | Yunnan | JN408350 | 18-YN-CHN-2009 | C4a2 |
| 2009 | Yunnan | JN408351 | 19-YN-CHN-2009 | C4a2 |
| 2009 | Yunnan | JN408352 | 21-YN-CHN-2009 | C4a2 |
| 2009 | Yunnan | JN408353 | 22-YN-CHN-2009 | C4a2 |
| 2009 | Yunnan | JN408354 | 25-YN-CHN-2009 | C4a2 |
| 2009 | Yunnan | JN408355 | 26-YN-CHN-2009 | C4a2 |
| 2009 | Yunnan | JN408356 | 27-YN-CHN-2009 | C4a2 |
| 2009 | Yunnan | JN408357 | 29-YN-CHN-2009 | C4a2 |
| 2009 | Yunnan | JN408358 | 34-YN-CHN-2009 | C4a2 |
| 2009 | Yunnan | HQ423142 | KMM/09 | C4a2 |
| 2009 | Yunnan | HQ423143 | KM186/09 | C4a2 |
| 2009 | Lanzhou | GU396280 | EV71/Lanzhou01 | C4a2 |
| 2009 | Zhejiang | GU129025 | EV71.H3-TY | C4a2 |
| 2009 | Zhejiang | GU129022 | EV71.H3-TY | C4a2 |
| 2009 | Zhejiang | GU129023 | EV71.H3-TY | C4a2 |
| 2009 | Zhejiang | GU129024 | EV71.H3-TY | C4a2 |
| 2009 | Zhejiang | JN168790 | EV71/Ningbo.CHN/001/2009 | C4a2 |
| 2009 | Chongqing | GQ994989 | Chongqing1-09-China | C4a2 |
| 2009 | Chongqing | GQ994990 | Chongqing2-09-China | C4a2 |
| 2009 | Chongqing | GQ994991 | Chongqing3-09-China | C4a2 |
| 2010 | Anhui | JF937671 | 390-Luan(CHN)-10 | C4a2 |
| 2010 | Anhui | JF937670 | 389-Luan(CHN)-10 | C4a2 |
| 2010 | Anhui | JF937666 | 74-Luan(CHN)-10 | C4a2 |
| 2010 | Anhui | HQ694986 | MP10-2010 | C4a2 |
| 2010 | Anhui | JF937667 | 79-Luan(CHN)-10 | C4a2 |
| 2010 | Anhui | JF820315 | AH01 | C4a2 |
| 2010 | Anhui | JF937672 | 427-Luan(CHN)-10 | C4a2 |
| 2010 | Anhui | JF937669 | 126-Luan(CHN)-10 | C4a2 |
| 2010 | Anhui | JF937668 | 122-Luan(CHN)-10 | C4a2 |
| 2010 | Anhui | JF937665 | 66-Luan(CHN)-10 | C4a2 |
| 2010 | Guangxi | HQ428125 | GX10/33 | C4a2 |
| 2010 | Fujian | HQ426649 | 2010FJLY008 | C4a2 |
| 2010 | Guangdong | HQ456305 | EV71/Guangzhou/156/2010 | C4a2 |
| 2010 | Guangdong | HQ456306 | EV71/Guangzhou/134/2010 | C4a2 |
| 2010 | Guangdong | HQ456307 | EV71/Guangzhou/120/2010 | C4a2 |
| 2010 | Guangdong | HQ456308 | EV71/Guangzhou/118/2010 | C4a2 |
| 2010 | Guangdong | HQ456313 | EV71/Guangzhou/95/2010 | C4a2 |
| 2010 | Guangdong | JF519708 | 224/GD/China/2010 | C4a2 |
| 2010 | Guangdong | JF519709 | 16/GD/China/2010 | C4a2 |
| 2010 | Guangdong | JF519710 | 19/GD/China/2010 | C4a2 |
| 2010 | Guangdong | JF519711 | 30/GD/China/2010 | C4a2 |
| 2010 | Guangdong | JF519712 | 31/GD/China/2010 | C4a2 |
| 2010 | Guangdong | JF519713 | 210/GD/China/2010 | C4a2 |
| 2010 | Guangdong | JF519714 | 15/GD/China/2010 | C4a2 |
| 2010 | Guangdong | JF519715 | 1134/GD/China/2010 | C4a2 |
| 2010 | Guangdong | JF519716 | 1131/GD/China/2010 | C4a2 |
| 2010 | Guangdong | JF519717 | 1127/GD/China/2010 | C4a2 |
| 2010 | Guangdong | JF519718 | 1111/GD/China/2010 | C4a2 |
| 2010 | Guangdong | JF519719 | 997/GD/China/2010 | C4a2 |
| 2010 | Beijing | JF820312 | LCH01 | C4a2 |
| 2010 | Beijing | JF820313 | LCH02 | C4a2 |
| 2010 | Beijing | JF820314 | BJ02 | C4a2 |
| 2010 | Beijing | JF820316 | BJ01 | C4a2 |
| 2010 | Hebei | JN256066 | HeB-310/HeB/CHN/2010 | C4a2 |
| 2010 | Hebei | JN256065 | HeB-132/HeB/CHN/2010 | C4a2 |
| 2010 | Henan | HM245927 | EV71/Henan/294/2010 | C4a2 |
| 2010 | Henan | HQ668272 | 1232/HN/CHN/2010 | C4a2 |
| 2010 | Henan | HQ668275 | 1211/HN/CHN/2010 | C4a2 |
| 2010 | Henan | HQ668183 | 1204/HN/CHN/2010 | C4a2 |
| 2010 | Henan | HQ668184 | 1278/HN/CHN/2010 | C4a2 |
| 2010 | Henan | HQ668224 | 1217/HN/CHN/2010 | C4a2 |
| 2010 | Henan | HQ668255 | 1203/HN/CHN/2010 | C4a2 |
| 2010 | Henan | HQ668276 | 1228/HN/CHN/2010 | C4a2 |
| 2010 | Henan | HQ668273 | 1208/HN/CHN/2010 | C4a2 |
| 2010 | Henan | HQ668302 | 1256/HN/CHN/2010 | C4a2 |
| 2010 | Henan | HQ668303 | 1230/HN/CHN/2010 | C4a2 |
| 2010 | Henan | HQ325852 | EV71/HENAN/DC/2010 | C4a2 |
| 2010 | Henan | HQ668223 | 1245/HN/CHN/2010 | C4a2 |
| 2010 | Henan | HQ668191 | 1221/HN/CHN/2010 | C4a2 |
| 2010 | Henan | HQ668189 | 1248/HN/CHN/2010 | C4a2 |
| 2010 | Henan | HQ668254 | 1207/HN/CHN/2010 | C4a2 |
| 2010 | Henan | HQ668249 | 1239/HN/CHN/2010 | C4a2 |
| 2010 | Henan | HQ668248 | 1265/HN/CHN/2010 | C4a2 |
| 2010 | Henan | HQ668247 | 1262/HN/CHN/2010 | C4a2 |
| 2010 | Henan | HQ668305 | 1226/HN/CHN/2010 | C4a2 |
| 2010 | Henan | HQ668309 | 1240/HN/CHN/2010 | C4a2 |
| 2010 | Henan | HQ668310 | 1206/HN/CHN/2010 | C4a2 |
| 2010 | Henan | HQ668312 | 1255/HN/CHN/2010 | C4a2 |
| 2010 | Henan | HQ668317 | 1267/HN/CHN/2010 | C4a2 |
| 2010 | Henan | HQ668328 | 1247/HN/CHN/2010 | C4a2 |
| 2010 | Henan | HQ668359 | 1220/HN/CHN/2010 | C4a2 |
| 2010 | Henan | HQ668362 | 1257/HN/CHN/2010 | C4a2 |
| 2010 | Henan | HQ668372 | 1283/HN/CHN/2010 | C4a2 |
| 2010 | Henan | HQ668373 | 1281/HN/CHN/2010 | C4a2 |
| 2010 | Henan | HQ668374 | 1253/HN/CHN/2010 | C4a2 |
| 2010 | Henan | HQ668377 | 1225/HN/CHN/2010 | C4a2 |
| 2010 | Henan | HQ668394 | 1202/HN/CHN/2010 | C4a2 |
| 2010 | Henan | HQ668405 | 1242/HN/CHN/2010 | C4a2 |
| 2010 | Henan | HQ668409 | 1215/HN/CHN/2010 | C4a2 |
| 2010 | Shandong | HQ668410 | 1224/HN/CHN/2010 | C4a2 |
| 2010 | Henan | HQ668413 | 1212/HN/CHN/2010 | C4a2 |
| 2010 | Henan | HQ668425 | 1260/HN/CHN/2010 | C4a2 |
| 2010 | Henan | HQ668426 | 1213/HN/CHN/2010 | C4a2 |
| 2010 | Henan | HQ668427 | 1238/HN/CHN/2010 | C4a2 |
| 2010 | Henan | HQ668428 | 1241/HN/CHN/2010 | C4a2 |
| 2010 | Henan | HQ668429 | 1234/HN/CHN/2010 | C4a2 |
| 2010 | Henan | HQ668430 | 1235/HN/CHN/2010 | C4a2 |
| 2010 | Henan | HQ668431 | 1229/HN/CHN/2010 | C4a2 |
| 2010 | Henan | HQ668432 | 1222/HN/CHN/2010 | C4a2 |
| 2010 | Henan | HQ668435 | 1219/HN/CHN/2010 | C4a2 |
| 2010 | Henan | HQ668436 | 1209/HN/CHN/2010 | C4a2 |
| 2010 | Henan | HQ668448 | 1218/HN/CHN/2010 | C4a2 |
| 2010 | Henan | HQ668449 | 1259/HN/CHN/2010 | C4a2 |
| 2010 | Henan | HQ668451 | 1264/HN/CHN/2010 | C4a2 |
| 2010 | Henan | HQ668452 | 1273/HN/CHN/2010 | C4a2 |
| 2010 | Henan | HQ668453 | 1231/HN/CHN/2010 | C4a2 |
| 2010 | Henan | HQ668454 | 1275/HN/CHN/2010 | C4a2 |
| 2010 | Henan | HQ668455 | 1279/HN/CHN/2010 | C4a2 |
| 2010 | Henan | HQ668456 | 1268/HN/CHN/2010 | C4a2 |
| 2010 | Henan | HQ668457 | 1274/HN/CHN/2010 | C4a2 |
| 2010 | Hubei | JQ419491 | ensh6-CHN-10 | C4a2 |
| 2010 | Hubei | JQ419492 | ensh7-CHN-10 | C4a2 |
| 2010 | Hubei | JQ419493 | ensh8-CHN-10 | C4a2 |
| 2010 | Hubei | JQ419494 | ensh11-CHN-10 | C4a2 |
| 2010 | Hubei | JQ419495 | wh28-CHN-10 | C4a2 |
| 2010 | Hubei | JQ419496 | wh29-CHN-10 | C4a2 |
| 2010 | Hubei | JQ419497 | wh61-CHN-10 | C4a2 |
| 2010 | Hubei | JQ419498 | wh62-CHN-10 | C4a2 |
| 2010 | Hubei | JQ419499 | wh64-CHN-10 | C4a2 |
| 2010 | Hunan | HM776019 | 237/Changsha/China/HFMD/2010 | C4a2 |
| 2010 | Hunan | HM776020 | 253/Changsha/China/HFMD/2010 | C4a2 |
| 2010 | Hunan | HM776021 | 338/Changsha/China/HFMD/2010 | C4a2 |
| 2010 | Hunan | HM776022 | 340/Changsha/China/HFMD/2010 | C4a2 |
| 2010 | Hunan | HM776023 | 342/Changsha/China/HFMD/2010 | C4a2 |
| 2010 | Hunan | HM776024 | 357/Changsha/China/HFMD/2010 | C4a2 |
| 2010 | Hunan | HM776025 | 358/Changsha/China/HFMD/2010 | C4a2 |
| 2010 | Hunan | HM776026 | 400/Changsha/China/HFMD/2010 | C4a2 |
| 2010 | Hunan | HM776027 | 410/Changsha/China/HFMD/2010 | C4a2 |
| 2010 | Hunan | HM776028 | 426/Changsha/China/HFMD/2010 | C4a2 |
| 2010 | Hunan | HM776029 | 430/Changsha/China/HFMD/2010 | C4a2 |
| 2010 | Hunan | HM776030 | 447/Changsha/China/HFMD/2010 | C4a2 |
| 2010 | Liaoning | HQ407557 | LN009 | C4a2 |
| 2010 | Nanyang | JF508447 | Nanyang01-2010 | C4a2 |
| 2010 | Xiamen | HQ850973 | 003/XM/2010 | C4a2 |
| 2010 | Shandong | HQ668182 | 489/SD/CHN/2010 | C4a2 |
| 2010 | Shandong | HQ668186 | 501/SD/CHN/2010 | C4a2 |
| 2010 | Shandong | HQ668192 | 484/SD/CHN/2010 | C4a2 |
| 2010 | Shandong | HQ668194 | 434/SD/CHN/2010 | C4a2 |
| 2010 | Shandong | HQ668197 | 381/SD/CHN/2010 | C4a2 |
| 2010 | Shandong | HQ668198 | 256/SD/CHN/2010 | C4a2 |
| 2010 | Shandong | HQ668199 | 589/SD/CHN/2010 | C4a2 |
| 2010 | Shandong | HQ668200 | 629/SD/CHN/2010 | C4a2 |
| 2010 | Shandong | HQ668202 | 578/SD/CHN/2010 | C4a2 |
| 2010 | Shandong | HQ668203 | 597/SD/CHN/2010 | C4a2 |
| 2010 | Shandong | HQ668204 | 211/SD/CHN/2010 | C4a2 |
| 2010 | Shandong | HQ668206 | 379/SD/CHN/2010 | C4a2 |
| 2010 | Shandong | HQ668207 | 385/SD/CHN/2010 | C4a2 |
| 2010 | Shandong | HQ668212 | 652/SD/CHN/2010 | C4a2 |
| 2010 | Shandong | HQ668214 | 476/SD/CHN/2010 | C4a2 |
| 2010 | Shandong | HQ668215 | 865/SD/CHN/2010 | C4a2 |
| 2010 | Shandong | HQ668220 | 611/SD/CHN/2010 | C4a2 |
| 2010 | Shandong | HQ668225 | 374/SD/CHN/2010 | C4a2 |
| 2010 | Shandong | HQ668226 | 624/SD/CHN/2010 | C4a2 |
| 2010 | Shandong | HQ668230 | 625/SD/CHN/2010 | C4a2 |
| 2010 | Shandong | HQ668231 | 220/SD/CHN/2010 | C4a2 |
| 2010 | Shandong | HQ668233 | 410/SD/CHN/2010 | C4a2 |
| 2010 | Shandong | HQ668234 | 411/SD/CHN/2010 | C4a2 |
| 2010 | Shandong | HQ668235 | 621/SD/CHN/2010 | C4a2 |
| 2010 | Shandong | HQ668236 | 654/SD/CHN/2010 | C4a2 |
| 2010 | Shandong | HQ668237 | 388/SD/CHN/2010 | C4a2 |
| 2010 | Shandong | HQ668238 | 464/SD/CHN/2010 | C4a2 |
| 2010 | Shandong | HQ668241 | 359/SD/CHN/2010 | C4a2 |
| 2010 | Shandong | HQ668242 | 371/SD/CHN/2010 | C4a2 |
| 2010 | Shandong | HQ668246 | 478/SD/CHN/2010 | C4a2 |
| 2010 | Shandong | HQ668250 | 497/SD/CHN/2010 | C4a2 |
| 2010 | Shandong | HQ668251 | 442/SD/CHN/2010 | C4a2 |
| 2010 | Shandong | HQ668267 | 453/SD/CHN/2010 | C4a2 |
| 2010 | Shandong | HQ668269 | 365/SD/CHN/2010 | C4a2 |
| 2010 | Shandong | HQ668270 | 655/SD/CHN/2010 | C4a2 |
| 2010 | Shandong | HQ668271 | 462/SD/CHN/2010 | C4a2 |
| 2010 | Shandong | HQ668274 | 562/SD/CHN/2010 | C4a2 |
| 2010 | Shandong | HQ668278 | 360/SD/CHN/2010 | C4a2 |
| 2010 | Shandong | HQ668280 | 469/SD/CHN/2010 | C4a2 |
| 2010 | Shandong | HQ668281 | 393/SD/CHN/2010 | C4a2 |
| 2010 | Shandong | HQ668282 | 604/SD/CHN/2010 | C4a2 |
| 2010 | Shandong | HQ668283 | 636/SD/CHN/2010 | C4a2 |
| 2010 | Shandong | HQ668290 | 378/SD/CHN/2010 | C4a2 |
| 2010 | Shandong | HQ668306 | 443/SD/CHN/2010 | C4a2 |
| 2010 | Shandong | HQ668308 | 653/SD/CHN/2010 | C4a2 |
| 2010 | Shandong | HQ668315 | 345/SD/CHN/2010 | C4a2 |
| 2010 | Shandong | HQ668318 | 403/SD/CHN/2010 | C4a2 |
| 2010 | Shandong | HQ668321 | 617/SD/CHN/2010 | C4a2 |
| 2010 | Shandong | HQ668322 | 619/SD/CHN/2010 | C4a2 |
| 2010 | Shandong | HQ668323 | 627/SD/CHN/2010 | C4a2 |
| 2010 | Shandong | HQ668324 | 635/SD/CHN/2010 | C4a2 |
| 2010 | Shandong | HQ668326 | 579/SD/CHN/2010 | C4a2 |
| 2010 | Shandong | HQ668327 | 373/SD/CHN/2010 | C4a2 |
| 2010 | Shandong | HQ668330 | 500/SD/CHN/2010 | C4a2 |
| 2010 | Shandong | HQ668331 | 383/SD/CHN/2010 | C4a2 |
| 2010 | Shandong | HQ668332 | 209/SD/CHN/2010 | C4a2 |
| 2010 | Shandong | HQ668334 | 214/SD/CHN/2010 | C4a2 |
| 2010 | Shandong | HQ668342 | 444/SD/CHN/2010 | C4a2 |
| 2010 | Shandong | HQ668343 | 618/SD/CHN/2010 | C4a2 |
| 2010 | Shandong | HQ668352 | 628/SD/CHN/2010 | C4a2 |
| 2010 | Shandong | HQ668353 | 620/SD/CHN/2010 | C4a2 |
| 2010 | Shandong | HQ668354 | 580/SD/CHN/2010 | C4a2 |
| 2010 | Shandong | HQ668355 | 361/SD/CHN/2010 | C4a2 |
| 2010 | Shandong | HQ668357 | 496/SD/CHN/2010 | C4a2 |
| 2010 | Shandong | HQ668360 | 215/SD/CHN/2010 | C4a2 |
| 2010 | Shandong | HQ668361 | 415/SD/CHN/2010 | C4a2 |
| 2010 | Shandong | HQ668363 | 351/SD/CHN/2010 | C4a2 |
| 2010 | Shandong | HQ668364 | 245/SD/CHN/2010 | C4a2 |
| 2010 | Shandong | HQ668365 | 395/SD/CHN/2010 | C4a2 |
| 2010 | Shandong | HQ668366 | 336/SD/CHN/2010 | C4a2 |
| 2010 | Shandong | HQ668367 | 341/SD/CHN/2010 | C4a2 |
| 2010 | Shandong | HQ668368 | 333/SD/CHN/2010 | C4a2 |
| 2010 | Shandong | HQ668369 | 419/SD/CHN/2010 | C4a2 |
| 2010 | Shandong | HQ668370 | 343/SD/CHN/2010 | C4a2 |
| 2010 | Shandong | HQ668376 | 646/SD/CHN/2010 | C4a2 |
| 2010 | Shandong | HQ668380 | 390/SD/CHN/2010 | C4a2 |
| 2010 | Shandong | HQ668381 | 264/SD/CHN/2010 | C4a2 |
| 2010 | Shandong | HQ668382 | 375/SD/CHN/2010 | C4a2 |
| 2010 | Shandong | HQ668383 | 428/SD/CHN/2010 | C4a2 |
| 2010 | Shandong | HQ668384 | 399/SD/CHN/2010 | C4a2 |
| 2010 | Shandong | HQ668385 | 392/SD/CHN/2010 | C4a2 |
| 2010 | Shandong | HQ668386 | 658/SD/CHN/2010 | C4a2 |
| 2010 | Shandong | HQ668387 | 435/SD/CHN/2010 | C4a2 |
| 2010 | Shandong | HQ668388 | 432/SD/CHN/2010 | C4a2 |
| 2010 | Shandong | HQ668389 | 397/SD/CHN/2010 | C4a2 |
| 2010 | Shandong | HQ668390 | 594/SD/CHN/2010 | C4a2 |
| 2010 | Shandong | HQ668391 | 605/SD/CHN/2010 | C4a2 |
| 2010 | Shandong | HQ668392 | 630/SD/CHN/2010 | C4a2 |
| 2010 | Shandong | HQ668393 | 622/SD/CHN/2010 | C4a2 |
| 2010 | Shandong | HQ668396 | 400/SD/CHN/2010 | C4a2 |
| 2010 | Shandong | HQ668397 | 367/SD/CHN/2010 | C4a2 |
| 2010 | Shandong | HQ668398 | 420/SD/CHN/2010 | C4a2 |
| 2010 | Shandong | HQ668399 | 368/SD/CHN/2010 | C4a2 |
| 2010 | Shandong | HQ668400 | 459/SD/CHN/2010 | C4a2 |
| 2010 | Shandong | HQ668401 | 486/SD/CHN/2010 | C4a2 |
| 2010 | Shandong | HQ668403 | 355/SD/CHN/2010 | C4a2 |
| 2010 | Shandong | HQ668404 | 623/SD/CHN/2010 | C4a2 |
| 2010 | Shandong | HQ668407 | 631/SD/CHN/2010 | C4a2 |
| 2010 | Shandong | HQ668408 | 640/SD/CHN/2010 | C4a2 |
| 2010 | Shandong | HQ668411 | 572/SD/CHN/2010 | C4a2 |
| 2010 | Shandong | HQ668412 | 348/SD/CHN/2010 | C4a2 |
| 2010 | Shandong | HQ668414 | 319/SD/CHN/2010 | C4a2 |
| 2010 | Shandong | HQ668415 | 304/SD/CHN/2010 | C4a2 |
| 2010 | Shandong | HQ668416 | 402/SD/CHN/2010 | C4a2 |
| 2010 | Shandong | HQ668417 | 409/SD/CHN/2010 | C4a2 |
| 2010 | Shandong | HQ668418 | 354/SD/CHN/2010 | C4a2 |
| 2010 | Shandong | HQ668419 | 429/SD/CHN/2010 | C4a2 |
| 2010 | Shandong | HQ668420 | 389/SD/CHN/2010 | C4a2 |
| 2010 | Shandong | HQ668421 | 483/SD/CHN/2010 | C4a2 |
| 2010 | Shandong | HQ668422 | 384/SD/CHN/2010 | C4a2 |
| 2010 | Shandong | HQ668423 | 363/SD/CHN/2010 | C4a2 |
| 2010 | Shandong | HQ668424 | 626/SD/CHN/2010 | C4a2 |
| 2010 | Shandong | HQ668433 | 613/SD/CHN/2010 | C4a2 |
| 2010 | Shandong | HQ668434 | 238/SD/CHN/2010 | C4a2 |
| 2010 | Shandong | HQ668438 | 251/SD/CHN/2010 | C4a2 |
| 2010 | Shandong | HQ668440 | 372/SD/CHN/2010 | C4a2 |
| 2010 | Shandong | HQ668441 | 386/SD/CHN/2010 | C4a2 |
| 2010 | Shandong | HQ668442 | 647/SD/CHN/2010 | C4a2 |
| 2010 | Shandong | HQ668443 | 634/SD/CHN/2010 | C4a2 |
| 2010 | Shandong | HQ668444 | 405/SD/CHN/2010 | C4a2 |
| 2010 | Shandong | HQ668445 | 642/SD/CHN/2010 | C4a2 |
| 2010 | Shandong | HQ668446 | 458/SD/CHN/2010 | C4a2 |
| 2010 | Shandong | HQ668447 | 641/SD/CHN/2010 | C4a2 |
| 2010 | Shandong | HQ668450 | 338/SD/CHN/2010 | C4a2 |
| 2010 | Shandong | HQ668458 | 431/SD/CHN/2010 | C4a2 |
| 2010 | Shanghai | JF812997 | Shanghai10-qp1 | C4a2 |
| 2010 | Shanghai | JF918563 | SHAPHC435T/SH/CHN/10 | C4a2 |
| 2010 | Shanghai | JF918564 | SHAPHC443F/SH/CHN/10 | C4a2 |
| 2010 | Shanghai | JF918565 | SHAPHC460T/SH/CHN/10 | C4a2 |
| 2010 | Shanghai | JF918566 | SHAPHC478F/SH/CHN/10 | C4a2 |
| 2010 | Shanghai | JF918567 | SHAPHC502F/SH/CHN/10 | C4a2 |
| 2010 | Shanghai | JF918568 | SHAPHC521T/SH/CHN/10 | C4a2 |
| 2010 | Shanghai | JF918569 | SHAPHC528F/SH/CHN/10 | C4a2 |
| 2010 | Shanghai | JF918570 | SHAPHC532F/SH/CHN/10 | C4a2 |
| 2010 | Shanghai | JF918571 | SHAPHC534F/SH/CHN/10 | C4a2 |
| 2010 | Shanghai | JF918572 | SHAPHC538F/SH/CHN/10 | C4a2 |
| 2010 | Shanghai | JF918573 | SHAPHC539T/SH/CHN/10 | C4a2 |
| 2010 | Shanghai | JF918574 | SHAPHC540F/SH/CHN/10 | C4a2 |
| 2010 | Shanghai | JF918575 | SHAPHC565T/SH/CHN/10 | C4a2 |
| 2010 | Shanghai | JF918576 | SHAPHC571T/SH/CHN/10 | C4a2 |
| 2010 | Shanghai | JF918577 | SHAPHC578F/SH/CHN/10 | C4a2 |
| 2010 | Shanghai | JF918578 | SHAPHC581F/SH/CHN/10 | C4a2 |
| 2010 | Sichuan | AB679718 | SC10-25 | C4a2 |
| 2010 | Sichuan | AB679719 | SC10-67 | C4a2 |
| 2010 | Sichuan | AB679720 | SC10-68 | C4a2 |
| 2010 | Sichuan | AB679721 | SC10-70 | C4a2 |
| 2010 | Sichuan | AB679722 | SC10-102 | C4a2 |
| 2010 | Sichuan | AB679723 | SC10-71 | C4a2 |
| 2010 | Sichuan | AB679724 | SC10-90 | C4a2 |
| 2010 | Sichuan | AB679725 | SC10-96 | C4a2 |
| 2010 | Sichuan | AB679726 | SC10-27 | C4a2 |
| 2010 | Sichuan | AB679727 | SC10-53 | C4a2 |
| 2010 | Sichuan | AB679728 | SC10-54 | C4a2 |
| 2010 | Sichuan | AB679729 | SC10-62 | C4a2 |
| 2010 | Sichuan | AB679730 | SC10-64 | C4a2 |
| 2010 | Sichuan | AB679731 | SC10-131 | C4a2 |
| 2010 | Sichuan | AB679732 | SC10-229 | C4a2 |
| 2010 | Sichuan | AB679733 | SC10-227 | C4a2 |
| 2010 | Sichuan | AB679734 | SC10-236 | C4a2 |
| 2010 | Sichuan | AB679735 | SC10-239 | C4a2 |
| 2010 | Sichuan | AB679736 | SC10-246 | C4a2 |
| 2010 | Sichuan | AB679737 | SC10-255 | C4a2 |
| 2010 | Sichuan | AB679738 | SC10-256 | C4a2 |
| 2010 | Sichuan | AB679739 | SC10-259 | C4a2 |
| 2010 | Sichuan | AB679740 | SC10-267 | C4a2 |
| 2010 | Sichuan | AB679741 | SC10-268 | C4a2 |
| 2010 | Sichuan | AB679742 | SC10-274 | C4a2 |
| 2010 | Sichuan | AB679743 | SC10-290 | C4a2 |
| 2010 | Sichuan | AB679744 | SC10-294 | C4a2 |
| 2010 | Sichuan | AB675638 | DY2010-23 | C4a2 |
| 2010 | Sichuan | AB675639 | DY2010-38 | C4a2 |
| 2010 | Sichuan | HQ668181 | 735/CQ/CHN/2010 | C4a2 |
| 2010 | Sichuan | HQ668185 | 770/CQ/CHN/2010 | C4a2 |
| 2010 | Sichuan | HQ668187 | 756/CQ/CHN/2010 | C4a2 |
| 2010 | Sichuan | HQ668188 | 740/CQ/CHN/2010 | C4a2 |
| 2010 | Sichuan | HQ668190 | 711/CQ/CHN/2010 | C4a2 |
| 2010 | Sichuan | HQ668193 | 509/CQ/CHN/2010 | C4a2 |
| 2010 | Sichuan | HQ668195 | 705/CQ/CHN/2010 | C4a2 |
| 2010 | Sichuan | HQ668196 | 696/CQ/CHN/2010 | C4a2 |
| 2010 | Sichuan | HQ668201 | 746/CQ/CHN/2010 | C4a2 |
| 2010 | Sichuan | HQ668205 | 703/CQ/CHN/2010 | C4a2 |
| 2010 | Sichuan | HQ668208 | 170/CQ/CHN/2010 | C4a2 |
| 2010 | Sichuan | HQ668209 | 769/CQ/CHN/2010 | C4a2 |
| 2010 | Sichuan | HQ668210 | 533/CQ/CHN/2010 | C4a2 |
| 2010 | Sichuan | HQ668211 | 535/CQ/CHN/2010 | C4a2 |
| 2010 | Sichuan | HQ668213 | 800/CQ/CHN/2010 | C4a2 |
| 2010 | Sichuan | HQ668216 | 755/CQ/CHN/2010 | C4a2 |
| 2010 | Sichuan | HQ668217 | 765/CQ/CHN/2010 | C4a2 |
| 2010 | Sichuan | HQ668218 | 772/CQ/CHN/2010 | C4a2 |
| 2010 | Sichuan | HQ668219 | 712/CQ/CHN/2010 | C4a2 |
| 2010 | Sichuan | HQ668221 | 780/CQ/CHN/2010 | C4a2 |
| 2010 | Sichuan | HQ668222 | 803/CQ/CHN/2010 | C4a2 |
| 2010 | Sichuan | HQ668228 | 713/CQ/CHN/2010 | C4a2 |
| 2010 | Sichuan | HQ668229 | 771/CQ/CHN/2010 | C4a2 |
| 2010 | Sichuan | HQ668232 | 762/CQ/CHN/2010 | C4a2 |
| 2010 | Sichuan | HQ668239 | 790/CQ/CHN/2010 | C4a2 |
| 2010 | Sichuan | HQ668240 | 724/CQ/CHN/2010 | C4a2 |
| 2010 | Sichuan | HQ668243 | 701/CQ/CHN/2010 | C4a2 |
| 2010 | Sichuan | HQ668244 | 720/CQ/CHN/2010 | C4a2 |
| 2010 | Sichuan | HQ668245 | 684/CQ/CHN/2010 | C4a2 |
| 2010 | Sichuan | HQ668252 | 157/CQ/CHN/2010 | C4a2 |
| 2010 | Sichuan | HQ668253 | 190/CQ/CHN/2010 | C4a2 |
| 2010 | Sichuan | HQ668256 | 744/CQ/CHN/2010 | C4a2 |
| 2010 | Sichuan | HQ668257 | 759/CQ/CHN/2010 | C4a2 |
| 2010 | Sichuan | HQ668258 | 760/CQ/CHN/2010 | C4a2 |
| 2010 | Sichuan | HQ668259 | 686/CQ/CHN/2010 | C4a2 |
| 2010 | Sichuan | HQ668260 | 717/CQ/CHN/2010 | C4a2 |
| 2010 | Sichuan | HQ668262 | 667/CQ/CHN/2010 | C4a2 |
| 2010 | Sichuan | HQ668263 | 732/CQ/CHN/2010 | C4a2 |
| 2010 | Sichuan | HQ668264 | 734/CQ/CHN/2010 | C4a2 |
| 2010 | Sichuan | HQ668265 | 767/CQ/CHN/2010 | C4a2 |
| 2010 | Sichuan | HQ668266 | 662/CQ/CHN/2010 | C4a2 |
| 2010 | Sichuan | HQ668268 | 182/CQ/CHN/2010 | C4a2 |
| 2010 | Sichuan | HQ668277 | 526/CQ/CHN/2010 | C4a2 |
| 2010 | Sichuan | HQ668279 | 719/CQ/CHN/2010 | C4a2 |
| 2010 | Sichuan | HQ668284 | 527/CQ/CHN/2010 | C4a2 |
| 2010 | Sichuan | HQ668285 | 140/CQ/CHN/2010 | C4a2 |
| 2010 | Sichuan | HQ668286 | 141/CQ/CHN/2010 | C4a2 |
| 2010 | Sichuan | HQ668287 | 783/CQ/CHN/2010 | C4a2 |
| 2010 | Sichuan | HQ668288 | 531/CQ/CHN/2010 | C4a2 |
| 2010 | Sichuan | HQ668289 | 552/CQ/CHN/2010 | C4a2 |
| 2010 | Sichuan | HQ668291 | 670/CQ/CHN/2010 | C4a2 |
| 2010 | Sichuan | HQ668292 | 678/CQ/CHN/2010 | C4a2 |
| 2010 | Sichuan | HQ668293 | 731/CQ/CHN/2010 | C4a2 |
| 2010 | Sichuan | HQ668294 | 784/CQ/CHN/2010 | C4a2 |
| 2010 | Sichuan | HQ668295 | 117/CQ/CHN/2010 | C4a2 |
| 2010 | Sichuan | HQ668296 | 172/CQ/CHN/2010 | C4a2 |
| 2010 | Sichuan | HQ668297 | 835/CQ/CHN/2010 | C4a2 |
| 2010 | Sichuan | HQ668298 | 757/CQ/CHN/2010 | C4a2 |
| 2010 | Sichuan | HQ668299 | 801/CQ/CHN/2010 | C4a2 |
| 2010 | Sichuan | HQ668300 | 802/CQ/CHN/2010 | C4a2 |
| 2010 | Sichuan | HQ668301 | 697/CQ/CHN/2010 | C4a2 |
| 2010 | Sichuan | HQ668304 | 540/CQ/CHN/2010 | C4a2 |
| 2010 | Sichuan | HQ668307 | 729/CQ/CHN/2010 | C4a2 |
| 2010 | Sichuan | HQ668313 | 671/CQ/CHN/2010 | C4a2 |
| 2010 | Sichuan | HQ668314 | 674/CQ/CHN/2010 | C4a2 |
| 2010 | Sichuan | HQ668316 | 781/CQ/CHN/2010 | C4a2 |
| 2010 | Sichuan | HQ668319 | 176/CQ/CHN/2010 | C4a2 |
| 2010 | Sichuan | HQ668320 | 690/CQ/CHN/2010 | C4a2 |
| 2010 | Sichuan | HQ668325 | 659/CQ/CHN/2010 | C4a2 |
| 2010 | Sichuan | HQ668329 | 716/CQ/CHN/2010 | C4a2 |
| 2010 | Sichuan | HQ668333 | 698/CQ/CHN/2010 | C4a2 |
| 2010 | Sichuan | HQ668335 | 788/CQ/CHN/2010 | C4a2 |
| 2010 | Sichuan | HQ668336 | 661/CQ/CHN/2010 | C4a2 |
| 2010 | Sichuan | HQ668337 | 669/CQ/CHN/2010 | C4a2 |
| 2010 | Sichuan | HQ668338 | 668/CQ/CHN/2010 | C4a2 |
| 2010 | Sichuan | HQ668339 | 679/CQ/CHN/2010 | C4a2 |
| 2010 | Sichuan | HQ668340 | 776/CQ/CHN/2010 | C4a2 |
| 2010 | Sichuan | HQ668341 | 174/CQ/CHN/2010 | C4a2 |
| 2010 | Sichuan | HQ668344 | 736/CQ/CHN/2010 | C4a2 |
| 2010 | Sichuan | HQ668345 | 510/CQ/CHN/2010 | C4a2 |
| 2010 | Sichuan | HQ668346 | 513/CQ/CHN/2010 | C4a2 |
| 2010 | Sichuan | HQ668347 | 156/CQ/CHN/2010 | C4a2 |
| 2010 | Sichuan | HQ668348 | 160/CQ/CHN/2010 | C4a2 |
| 2010 | Sichuan | HQ668349 | 728/CQ/CHN/2010 | C4a2 |
| 2010 | Sichuan | HQ668350 | 544/CQ/CHN/2010 | C4a2 |
| 2010 | Sichuan | HQ668351 | 714/CQ/CHN/2010 | C4a2 |
| 2010 | Sichuan | HQ668356 | 785/CQ/CHN/2010 | C4a2 |
| 2010 | Sichuan | HQ668358 | 704/CQ/CHN/2010 | C4a2 |
| 2010 | Sichuan | HQ668371 | 677/CQ/CHN/2010 | C4a2 |
| 2010 | Sichuan | HQ668375 | 144/CQ/CHN/2010 | C4a2 |
| 2010 | Sichuan | HQ668378 | 676/CQ/CHN/2010 | C4a2 |
| 2010 | Sichuan | HQ668379 | 529/CQ/CHN/2010 | C4a2 |
| 2010 | Sichuan | HQ668395 | 673/CQ/CHN/2010 | C4a2 |
| 2010 | Sichuan | HQ668402 | 693/CQ/CHN/2010 | C4a2 |
| 2010 | Sichuan | HQ668406 | 818/CQ/CHN/2010 | C4a2 |
| 2010 | Sichuan | HQ668437 | 726/CQ/CHN/2010 | C4a2 |
| 2010 | Sichuan | HQ668439 | 152/CQ/CHN/2010 | C4a2 |
| 2010 | Yunnan | JN251918 | 5/EV71/YN/CHN/2010 | C4a2 |
| 2010 | Yunnan | JN251920 | 7/EV71/YN/CHN/2010 | C4a2 |
| 2010 | Yunnan | JN251921 | 8/EV71/YN/CHN/2010 | C4a2 |
| 2010 | Yunnan | JN251922 | 9/EV71/YN/CHN/2010 | C4a2 |
| 2010 | Yunnan | JN251923 | 10/EV71/YN/CHN/2010 | C4a2 |
| 2010 | Yunnan | JN251925 | 13-EV71-YN-CHN-2010 | C4a2 |
| 2010 | Yunnan | JN251926 | 14/EV71/YN/CHN/2010 | C4a2 |
| 2010 | Yunnan | JN251927 | 15/EV71/YN/CHN/2010 | C4a2 |
| 2010 | Yunnan | JN251929 | 17/EV71/YN/CHN/2010 | C4a2 |
| 2010 | Yunnan | JN251930 | 18/EV71/YN/CHN/2010 | C4a2 |
| 2010 | Yunnan | JN251931 | 20/EV71/YN/CHN/2010 | C4a2 |
| 2010 | Yunnan | JN251932 | 23/EV71/YN/CHN/2010 | C4a2 |
| 2010 | Yunnan | JN251933 | 24/EV71/YN/CHN/2010 | C4a2 |
| 2010 | Yunnan | JN251934 | 25/EV71/YN/CHN/2010 | C4a2 |
| 2010 | Yunnan | JN251935 | 27/EV71/YN/CHN/2010 | C4a2 |
| 2010 | Yunnan | JN251936 | 28/EV71/YN/CHN/2010 | C4a2 |
| 2010 | Yunnan | JN251937 | 29-EV71-YN-CHN-2010 | C4a2 |
| 2010 | Yunnan | JN251938 | 31/EV71/YN/CHN/2010 | C4a2 |
| 2010 | Yunnan | JN251939 | 32-EV71-YN-CHN-2010 | C4a2 |
| 2010 | Yunnan | JN251940 | 33/EV71/YN/CHN/2010 | C4a2 |
| 2010 | Yunnan | JN251941 | 36/EV71/YN/CHN/2010 | C4a2 |
| 2010 | Yunnan | JN251942 | 43/EV71/YN/CHN/2010 | C4a2 |
| 2010 | Yunnan | JN251943 | 44/EV71/YN/CHN/2010 | C4a2 |
| 2010 | Yunnan | JN256068 | T126/YN/CHN/2010 | C4a2 |
| 2010 | Zhejiang | HM855950 | 01-ZJ-CHN-Z-10 | C4a2 |
| 2010 | Zhejiang | HM855951 | 02-ZJ-CHN-Z-10 | C4a2 |
| 2010 | Zhejiang | HM855952 | 03-ZJ-CHN-Z-10 | C4a2 |
| 2010 | Zhejiang | HM855953 | 04-ZJ-CHN-Z-10 | C4a2 |
| 2010 | Zhejiang | HM855954 | 05-ZJ-CHN-Z-10 | C4a2 |
| 2010 | Zhejiang | HM855955 | 06-ZJ-CHN-Z-10 | C4a2 |
| 2010 | Zhejiang | HQ828086 | NBChina01 | C4a2 |
| 2010 | Zhejiang | JN001860 | NB/2010/01 | C4a2 |
| 2010 | Zhejiang | JF830007 | EV71/Ningbo.CHN/065/2010 | C4a2 |
| 2010 | Shanxi | JN256067 | SX006/SX/CHN/2010 | C4a2 |
| 2010 | Wuhan | KF501389 | EV71/wuhan/3018/2010 | A |
| 2011 | Sichuan | AB675640 | DY2011-24 | C4a2 |
| 2011 | Sichuan | AB675641 | DY2011-25 | C4a2 |
| 2011 | Sichuan | AB675642 | DY2011-27 | C4a2 |
| 2011 | Sichuan | AB675643 | DY2011-41 | C4a2 |
| 2011 | Sichuan | AB675644 | DY2011-42 | C4a2 |
| 2011 | Sichuan | AB675645 | DY2011-45 | C4a2 |
| 2011 | Sichuan | AB675646 | DY2011-55 | C4a2 |
| 2011 | Sichuan | AB675647 | DY2011-126 | C4a2 |
| 2011 | Sichuan | AB675648 | DY2011-140 | C4a2 |
| 2011 | Sichuan | AB675649 | DY2011-145 | C4a2 |
| 2011 | Sichuan | AB675650 | DY2011-149 | C4a2 |
| 2011 | Sichuan | AB675651 | DY2011-151 | C4a2 |
| 2011 | Beijing | JF894381 | MP9-1 | C4a2 |
| 2011 | Beijing | JF894382 | MP9-2 | C4a2 |
| 2011 | Beijing | JF894383 | MP10-2 | C4a2 |
| 2011 | Henan | JN020147 | Luoyang/2011 | C4a2 |
| 2011 | Henan | JN052925 | Nanyang/2011-China | C4a2 |
| 2011 | Zhejiang | JN205805 | Hangzhou-15-2011 | C4a2 |
| 2011 | Zhejiang | JN205806 | Hangzhou-23-2011 | C4a2 |
| 2011 | Zhejiang | JN205807 | Hangzhou-40-2011 | C4a2 |
| 2011 | Zhejiang | JN205808 | Hangzhou-41-2011 | C4a2 |
| 2011 | Shan’xi | JN712916 | SX/EV-4/EV71/2011 | C4a2 |
| 2011 | Zhejiang | JQ086365 | EV71/Ningbo.CHN/107-2/2009 | C4a2 |
| 2011 | Zhejiang | JQ086366 | EV71/Ningbo.CHN/061/2011 | C4a2 |
| 2011 | Zhejiang | JQ284022 | Ningbo.CHN/cx005/2011 | C4a2 |
| 2011 | Zhejiang | JQ315061 | Ningbo.CHN/015/2011 | C4a2 |
| 2011 | Zhejiang | JQ315063 | Ningbo.CHN/046/2011 | C4a2 |
| 2011 | Zhejiang | JQ315066 | Ningbo.CHN/061/2011 | C4a2 |
| 2011 | Zhejiang | JQ315068 | Ningbo.CHN/044/2011 | C4a2 |
| 2011 | Zhejiang | JQ315070 | Ningbo.CHN/108/2011 | C4a2 |
| 2011 | Zhejiang | JQ315071 | Ningbo.CHN/111/2011 | C4a2 |
| 2011 | Zhejiang | JQ315073 | Ningbo.CHN/199/2011 | C4a2 |
| 2011 | Zhejiang | JQ315075 | Ningbo.CHN/332/2011 | C4a2 |
| 2011 | Zhejiang | JQ315078 | Ningbo.CHN/004/2011 | C4a2 |
| 2011 | Zhejiang | JQ315091 | Cixi.CHN/012/2011 | C4a2 |
| 2011 | Zhejiang | JQ315092 | Cixi.CHN/015/2011 | C4a2 |
| 2011 | Zhejiang | JQ315093 | Cixi.CHN/016/2011 | C4a2 |
| 2011 | Kunming | JQ316638 | HQ09231463 | C4a2 |
| 2011 | Henan | JQ639383 | HN1360/HN/CHN/2011 | C4a2 |
| 2011 | Henan | JQ639384 | HN318/HN/CHN/2011 | C4a2 |
| 2011 | Shanghai | JQ766140 | SCDC012011 | C4a2 |
| 2011 | Shanghai | JQ766141 | SCDC022011 | C4a2 |
| 2011 | Shanghai | JQ766142 | SCDC032011 | C4a2 |
| 2011 | Shanghai | JQ766143 | SCDC042011 | C4a2 |
| 2011 | Shanghai | JQ766144 | SCDC052011 | C4a2 |
| 2011 | Shanghai | JQ766145 | SCDC062011 | C4a2 |
| 2011 | Shanghai | JQ766146 | SCDC072011 | C4a2 |
| 2011 | Shanghai | JQ766147 | SCDC082011 | C4a2 |
| 2011 | Shanghai | JQ766148 | SCDC092011 | C4a2 |
| 2011 | Shanghai | JQ766190 | Q1EV | C4a2 |
| 2011 | Shanghai | JQ766191 | Q3EV | C4a2 |
| 2011 | Shanghai | JQ766192 | WHEV | C4a2 |
| 2011 | Shanghai | JQ766193 | XF1 | C4a2 |
| 2011 | Shanghai | JQ766194 | XF3 | C4a2 |
| 2011 | Shanghai | JQ766195 | XFE3 | C4a2 |
| 2011 | Shanghai | JQ766196 | XFE4 | C4a2 |
| 2011 | Jiangxi | JQ806378 | 35/Jingdezhen/China/HFMD_Severe/2011 | C4a2 |
| 2011 | Hubei | JQ906802 | EV71/XY4/2011 | C4a2 |
| 2011 | Hubei | JQ906803 | EV71/XY5/2011 | C4a2 |
| 2011 | Hubei | JQ906804 | EV71/XYZZ8/2011 | C4a2 |
| 2011 | Hubei | JQ906805 | EV71/XY18/2011 | C4a2 |
| 2011 | Hubei | JQ906806 | EV71/XY20/2011 | C4a2 |
| 2011 | Shandong | JQ922517 | Chi11-EV71-74jn | C4a2 |
| 2011 | Shandong | JQ922518 | Chi11-EV71-96jn | C4a2 |
| 2011 | Shandong | JQ922519 | Chi11-EV71-97jn | C4a2 |
| 2011 | Shandong | JQ922520 | Chi11-EV71-211jn | C4a2 |
| 2011 | Shanxi | JX014230 | SX/EV-5/EV71/2011 | C4a2 |
| 2011 | Henan | JX017384 | 01011Y | C4a2 |
| 2011 | Hubei | JX075098 | EV71/XY4/2011 | C4a2 |
| 2011 | Guangdong | JX103502 | Huizhou-83 | C4a2 |
| 2011 | Guangdong | JX103503 | 117/HZ/GD/CHN/2011 | C4a2 |
| 2011 | Guangdong | JX103504 | 292/HZ/GD/CHN | C4a2 |
| 2011 | Guangdong | JX103505 | 440/HZ/GD/CHN | C4a2 |
| 2011 | Guangdong | JX103506 | 602/HZ/GD/CHN | C4a2 |
| 2011 | Guangdong | JX111888 | GD-DG2011-1 | C4a2 |
| 2011 | Guangdong | JX111889 | GD-DG2011-2 | C4a2 |
| 2011 | Guangdong | JX111890 | GD-DG2011-3 | C4a2 |
| 2011 | Guangdong | JX111891 | GD-DG2011-5 | C4a2 |
| 2011 | Guangdong | JX111892 | GD-DG2011-8 | C4a2 |
| 2011 | Guangdong | JX111893 | GD-DG2011-9 | C4a2 |
| 2011 | Henan | JX155254 | HENXX01/2011 | C4a2 |
| 2011 | Henan | JX155255 | HENXX02/2011 | C4a2 |
| 2011 | Henan | JX155256 | HENXX03/2011 | C4a2 |
| 2011 | Henan | JX155257 | HENXX04/2011 | C4a2 |
| 2011 | Henan | JX155258 | HENXX05/2011 | C4a2 |
| 2011 | Henan | JX155259 | HENXX06/2011 | C4a2 |
| 2011 | Henan | JX155260 | HENXX07/2011 | C4a2 |
| 2011 | Henan | JX155261 | HENXX08/2011 | C4a2 |
| 2011 | Henan | JX155262 | HENXX09/2011 | C4a2 |
| 2011 | Henan | JX155263 | HENXX10/2011 | C4a2 |
| 2011 | Guizhou | JX203260 | AS17-2011 | C4a2 |
| 2011 | Guizhou | JX203261 | AS69-2011 | C4a2 |
| 2011 | Guizhou | JX203262 | AS159-2011 | C4a2 |
| 2011 | Guizhou | JX203263 | AS163-2011 | C4a2 |
| 2011 | Guizhou | JX203264 | BJ184-2011 | C4a2 |
| 2011 | Guizhou | JX203265 | GY25-2011 | C4a2 |
| 2011 | Guizhou | JX203266 | GY100-2011 | C4a2 |
| 2011 | Guizhou | JX203267 | GY309-2011 | C4a2 |
| 2011 | Guizhou | JX203268 | H5-2011 | C4a2 |
| 2011 | Guizhou | JX203269 | H38-2011 | C4a2 |
| 2011 | Guizhou | JX203270 | LPS8-2011 | C4a2 |
| 2011 | Guizhou | JX203271 | LPS11-2011 | C4a2 |
| 2011 | Guizhou | JX203272 | LPS42-2011 | C4a2 |
| 2011 | Guizhou | JX203273 | LPS62-2011 | C4a2 |
| 2011 | Guizhou | JX203274 | LPS126-2011 | C4a2 |
| 2011 | Guizhou | JX203275 | LPS153-2011 | C4a2 |
| 2011 | Guizhou | JX203276 | QDN74-2011 | C4a2 |
| 2011 | Guizhou | JX203277 | QN4-2011 | C4a2 |
| 2011 | Guizhou | JX203278 | QN61-2011 | C4a2 |
| 2011 | Guizhou | JX203279 | TR23-2011 | C4a2 |
| 2011 | Guizhou | JX203280 | TR52-2011 | C4a2 |
| 2011 | Guizhou | JX203281 | TR126-2011 | C4a2 |
| 2011 | Guizhou | JX203282 | ZY30-2011 | C4a2 |
| 2011 | Guizhou | JX203283 | ZY61-2011 | C4a2 |
| 2011 | Guizhou | JX203284 | ZY471-2011 | C4a2 |
| 2011 | Guizhou | JX203285 | ZY524-2011 | C4a2 |
| 2011 | Guizhou | JX203286 | ZY554-2011 | C4a2 |
| 2011 | Guizhou | JX203287 | ZY619-2011 | C4a2 |
| 2011 | Guizhou | JX203288 | ZY898-2011 | C4a2 |
| 2011 | Guangdong | JX473300 | EV71-SHZH2011-0509 | C4a2 |
| 2011 | Guangdong | JX473319 | EV71-SHZH2011-1001 | C4a2 |
| 2011 | Hunan | JX509919 | 201108 | C4a2 |
| 2011 | Hunan | JX509920 | 201107 | C4a2 |
| 2011 | Hunan | JX509921 | 201106 | C4a2 |
| 2011 | Hunan | JX509922 | 201105 | C4a2 |
| 2011 | Hunan | JX509923 | 201104 | C4a2 |
| 2011 | Hunan | JX509924 | 201103 | C4a2 |
| 2011 | Hunan | JX509925 | 201102 | C4a2 |
| 2011 | Hunan | JX509926 | 201101 | C4a2 |
| 2011 | Guangdong | JX961705 | 226/HZ/GD/CHN | C4a2 |
| 2011 | Guangdong | JX961706 | 233/HZ/GD/CHN | C4a2 |
| 2011 | Hubei | JX986737 | Wuhan1042/HuB/CHN/2011 | C4a2 |
| 2011 | Hubei | JX986738 | Wuhan1117/HuB/CHN/2011 | C4a2 |
| 2011 | Hubei | JX986739 | Wuhan1143/HuB/CHN/2011 | C4a2 |
| 2011 | Jiangxi | KC007533 | NC399/NC/CHN/2011 | C4a2 |
| 2011 | Jiangxi | KC007534 | NC429/NC/CHN/2011 | C4a2 |
| 2011 | Jiangxi | KC007536 | NC497/NC/CHN/2011 | C4a2 |
| 2011 | Jiangxi | KC007537 | NC434/NC/CHN/2011 | C4a2 |
| 2011 | Jiangxi | KC007538 | NC419/NC/CHN/2011 | C4a2 |
| 2011 | Jiangxi | KC007539 | NC500/NC/CHN/2011 | C4a2 |
| 2011 | Jiangxi | KC007540 | NC511/NC/CHN/2011 | C4a2 |
| 2011 | Jidezheng | KC109780 | 202/Jingdezhen/China/HFMD_Severe/2011 | C4a2 |
| 2011 | Kunming | KC414134 | JiLin-11-China | C4a2 |
| 2011 | Guangdong | KC801027 | GD-FS-985/2011 | C4a2 |
| 2011 | Guangdong | KC801028 | GD-FS-315/2011 | C4a2 |
| 2011 | Guangdong | KC801029 | GD-FS-094/2011 | C4a2 |
| 2011 | Guangdong | KC801033 | GD-FS-886/2011 | C4a2 |
| 2011 | Guangdong | KC801034 | GD-FS-882/2011 | C4a2 |
| 2011 | Guangdong | KC866764 | JB141130103 | C4a2 |
| 2011 | Guangdong | KC866765 | JB141130106 | C4a2 |
| 2011 | Guangdong | KC866766 | JB141130114 | C4a2 |
| 2011 | Guangdong | KC866767 | JB141130125 | C4a2 |
| 2011 | Guangdong | KC866768 | JB141130126 | C4a2 |
| 2011 | Guangdong | KC866769 | JB141130136 | C4a2 |
| 2011 | Guangdong | KC866770 | JB141130142 | C4a2 |
| 2011 | Guangdong | KC866771 | JB141130146 | C4a2 |
| 2011 | Guangdong | KC866772 | JB141130153 | C4a2 |
| 2011 | Guangdong | KC866773 | JB141130160 | C4a2 |
| 2011 | Guangdong | KC866774 | JB141130168 | C4a2 |
| 2011 | Guangdong | KC866775 | JB141130176 | C4a2 |
| 2011 | Guangdong | KC866776 | JB141130186 | C4a2 |
| 2011 | Guangdong | KC866777 | JB141130196 | C4a2 |
| 2011 | Shandong | KF150174 | 202/LY/CHN/AM/11/EV71 | C4a2 |
| 2011 | Zhejiang | KF358275 | EV71-Jiaxing-1 | C4a2 |
| 2011 | Anhui | KF925273 | FL001T/AH/CHN/2011 | C4a2 |
| 2011 | Anhui | KF925274 | FL003T/AH/CHN/2011 | C4a2 |
| 2011 | Anhui | KF925275 | FL004T/AH/CHN/2011 | C4a2 |
| 2011 | Anhui | KF925276 | FL005T/AH/CHN/2011 | C4a2 |
| 2011 | Anhui | KF925277 | FL008T/AH/CHN/2011 | C4a2 |
| 2011 | Anhui | KF925278 | FL009T/AH/CHN/2011 | C4a2 |
| 2011 | Anhui | KF925279 | FL010T/AH/CHN/2011 | C4a2 |
| 2011 | Anhui | KF925280 | FL011T/AH/CHN/2011 | C4a2 |
| 2011 | Anhui | KF925281 | FL012T/AH/CHN/2011 | C4a2 |
| 2011 | Anhui | KF925282 | FL013T/AH/CHN/2011 | C4a2 |
| 2011 | Anhui | KF925283 | FL015T/AH/CHN/2011 | C4a2 |
| 2011 | Anhui | KF925284 | FL017T/AH/CHN/2011 | C4a2 |
| 2011 | Beijing | KJ004552 | EV71/Hun11-32/2011 | C4a2 |
| 2011 | Beijing | KJ004553 | EV71/Hun11-4/2011 | C4a2 |
| 2011 | Shanghai | KJ188623 | SHC058/CHN/2011 | C4a2 |
| 2011 | Shanghai | KJ188624 | SHC057/CHN/2011 | C4a2 |
| 2011 | Shanghai | KJ188625 | SHC055/CHN/2011 | C4a2 |
| 2011 | Shanghai | KJ188626 | SHC052/CHN/2011 | C4a2 |
| 2011 | Shanghai | KJ188627 | SHC014/CHN/2011 | C4a2 |
| 2011 | Shanghai | KJ188628 | SHC013/CHN/2011 | C4a2 |
| 2011 | Shanghai | KJ188629 | SHC011/CHN/2011 | C4a2 |
| 2011 | Shanghai | KJ188630 | SHC019/CHN/2011 | C4a2 |
| 2011 | Shanghai | KJ188631 | SHC032/CHN/2011 | C4a2 |
| 2011 | Shanghai | KJ188632 | SHC059/CHN/2011 | C4a2 |
| 2011 | Shanghai | KJ188633 | SHC046/CHN/2011 | C4a2 |
| 2011 | Shanghai | KJ188634 | SHC002/CHN/2011 | C4a2 |
| 2011 | Shanghai | KJ188635 | SHC040/CHN/2011 | C4a2 |
| 2011 | Shanghai | KJ188636 | SHC045/CHN/2011 | C4a2 |
| 2011 | Shanghai | KJ188637 | SHC077/CHN/2011 | C4a2 |
| 2011 | Shanghai | KJ188638 | SHC070/CHN/2011 | C4a2 |
| 2011 | Shanghai | KJ188639 | SHC068/CHN/2011 | C4a2 |
| 2011 | Shanghai | KJ188640 | SHC063/CHN/2011 | C4a2 |
| 2011 | Shanghai | KJ188641 | SHC082/CHN/2011 | C4a2 |
| 2011 | Shanghai | KJ188642 | SHC080/CHN/2011 | C4a2 |
| 2011 | Zhejiang | KJ632497 | wenzhou011 | C4a2 |
| 2011 | Zhejiang | KJ632498 | wenzhou256 | C4a2 |
| 2011 | Zhejiang | KJ632499 | wenzhou227 | C4a2 |
| 2011 | Shandong | KJ772423 | 11061/SD/CHN | C4a2 |
| 2011 | Shandong | KJ772424 | 11072/SD/CHN | C4a2 |
| 2011 | Shandong | KJ772425 | 11144C1/SD/CHN | C4a2 |
| 2011 | Shandong | KJ772426 | 11144C5/SD/CHN | C4a2 |
| 2011 | Shandong | KJ772427 | 11171/SD/CHN | C4a2 |
| 2011 | Shandong | KJ772428 | 11236/SD/CHN | C4a2 |
| 2011 | Shandong | KJ772429 | 11298C1/SD/CHN | C4a2 |
| 2011 | Shandong | KJ772430 | 11317/SD/CHN | C4a2 |
| 2011 | Shandong | KJ772431 | 11318/SD/CHN | C4a2 |
| 2011 | Shandong | KJ772432 | 11475C4/SD/CHN | C4a2 |
| 2011 | Zhejiang | KJ784495 | HuzhouE371/2011/CHN | C4a2 |
| 2011 | Henan | KM201232 | HeN-11-Sanmenxia-3 | C4a2 |
| 2011 | Henan | KM201233 | HeN-11-Sanmenxia-4 | C4a2 |
| 2011 | Henan | KM201234 | HeN-11-Sanmenxia-7 | C4a2 |
| 2011 | Henan | KM201235 | HeN-11-Sanmenxia-11 | C4a2 |
| 2011 | Henan | KM201236 | HeN-11-Sanmenxia-13 | C4a2 |
| 2011 | Henan | KM201237 | HeN-11-Sanmenxia-18 | C4a2 |
| 2011 | Henan | KM201238 | HeN-11-Sanmenxia-20 | C4a2 |
| 2011 | Henan | KM201239 | HeN-11-Sanmenxia-23 | C4a2 |
| 2011 | Henan | KM201240 | HeN-11-Sanmenxia-24 | C4a2 |
| 2011 | Henan | KM201241 | HeN-11-Sanmenxia-30 | C4a2 |
| 2011 | Henan | KM201242 | HeN-11-Sanmenxia-33 | C4a2 |
| 2011 | Henan | KM201243 | HeN-11-Sanmenxia-37 | C4a2 |
| 2011 | Henan | KM201244 | HeN-11-Sanmenxia-38 | C4a2 |
| 2011 | Henan | KM201245 | HeN-11-Sanmenxia-39 | C4a2 |
| 2011 | Henan | KM201246 | HeN-11-Sanmenxia-43 | C4a2 |
| 2011 | Henan | KM201247 | HeN-11-Sanmenxia-44 | C4a2 |
| 2011 | Henan | KM201248 | HeN-11-Sanmenxia-45 | C4a2 |
| 2011 | Henan | KM201249 | HeN-11-Sanmenxia-46 | C4a2 |
| 2011 | Henan | KM201250 | HeN-11-Sanmenxia-50 | C4a2 |
| 2011 | Henan | KM260012 | 7-Henan-2011 | C4a2 |
| 2011 | Henan | KM260013 | 9-Henan-2011 | C4a2 |
| 2011 | Henan | KM260014 | 143-Henan-2011 | C4a2 |
| 2011 | Henan | KM260015 | 153-Henan-2011 | C4a2 |
| 2011 | Henan | KM260016 | 170-Henan-2011 | C4a2 |
| 2011 | Henan | KM260017 | 173-Henan-2011 | C4a2 |
| 2011 | Henan | KM260018 | 208-Henan-2011 | C4a2 |
| 2011 | Henan | KM260019 | 512-Henan-2011 | C4a2 |
| 2011 | Henan | KM260020 | 533-Henan-2011 | C4a2 |
| 2011 | Zhejiang | KM408445 | 01Zhenjiang2011 | C4a2 |
| 2011 | Zhejiang | KM408446 | 02Zhenjiang2011 | C4a2 |
| 2011 | Zhejiang | KM408447 | 03Zhenjiang2011 | C4a2 |
| 2011 | Zhejiang | KM408448 | 04Zhenjiang2011 | C4a2 |
| 2011 | Zhejiang | KM408449 | 05Zhenjiang2011 | C4a2 |
| 2011 | Zhejiang | KM408450 | 06Zhenjiang2011 | C4a2 |
| 2011 | Zhejiang | KM408451 | 07Zhenjiang2011 | C4a2 |
| 2011 | Zhejiang | KM408452 | 08Zhenjiang2011 | C4a2 |
| 2011 | Zhejiang | KM408453 | 09Zhenjiang2011 | C4a2 |
| 2011 | Zhejiang | KM408454 | 10Zhenjiang2011 | C4a2 |
| 2011 | Zhejiang | KM408455 | 11Zhenjiang2011 | C4a2 |
| 2011 | Zhejiang | KM408456 | 12Zhenjiang2011 | C4a2 |
| 2011 | Jiangsu | KT327138 | 319/SZ/CHN/2011 | C4a2 |
| 2011 | Jiangsu | KT327139 | 311/SZ/CHN/2011 | C4a2 |
| 2011 | Jiangsu | KT327140 | 317/SZ/CHN/2011 | C4a2 |
| 2011 | Jiangsu | KT327143 | 156/SZ/CHN/2011 | C4a2 |
| 2011 | Jiangsu | KT717749 | 11F92_2011.02_TZ | C4a2 |
| 2011 | Jiangsu | KT717750 | 11F93_2011.03_TZ | C4a2 |
| 2011 | Jiangsu | KT717751 | 11MF11_2011.03_JJ | C4a2 |
| 2011 | Jiangsu | KT717752 | 11MG1_2011.01_GG | C4a2 |
| 2011 | Jiangsu | KT717753 | 11MM14_2011.06_JY | C4a2 |
| 2011 | Jiangsu | KT717754 | 11MA113_2011.06_TZ | C4a2 |
| 2011 | Jiangsu | KT717755 | 11MF27_2011.05_JJ | C4a2 |
| 2011 | Jiangsu | KT717756 | 11MG16_2011.06_GG | C4a2 |
| 2011 | Jiangsu | KT717757 | 11MF36_2011.06_JJ | C4a2 |
| 2011 | Jiangsu | KT717758 | 11MF47_2011.06_JJ | C4a2 |
| 2011 | Jiangsu | KT717759 | 11MM13_2011.06_JY | C4a2 |
| 2011 | Jiangsu | KT717760 | 11MA114_2011.06_TZ | C4a2 |
| 2011 | Jiangsu | KT717761 | 11MF46_2011.06_JJ | C4a2 |
| 2011 | Jiangsu | KT717762 | 11MF31_2011.05_JJ | C4a2 |
| 2011 | Jiangsu | KT717763 | 11F115_2011.06_TZ | C4a2 |
| 2011 | Jiangsu | KT717764 | 11MF24_2011.05_JJ | C4a2 |
| 2011 | Jiangsu | KT717765 | 11MH29_2011.07_TZ | C4a2 |
| 2011 | Jiangsu | KT717766 | 11MD21_2011.07_TZ | C4a2 |
| 2011 | Jiangsu | KT717767 | 11MG21_2011.07_GG | C4a2 |
| 2011 | Jiangsu | KT717768 | 11MM16_2011.07_JY | C4a2 |
| 2011 | Jiangsu | KT717769 | 11MM19_2011.07_JY | C4a2 |
| 2011 | Jiangsu | KT717770 | 11MH27_2011.07_TZ | C4a2 |
| 2011 | Jiangsu | KT717771 | 11MA123_2011.08_TZ | C4a2 |
| 2011 | Jiangsu | KT717772 | 11MA124_2011.08_TZ | C4a2 |
| 2011 | Jiangsu | KT717773 | 11MC43_2011.09_XH | C4a2 |
| 2011 | Jiangsu | KT717774 | 11MC45_2011.09_XH | C4a2 |
| 2011 | Jiangsu | KT717775 | 11MD28_2011.09_TZ | C4a2 |
| 2011 | Jiangsu | KT717776 | 11MJ58_2011.12_TX | C4a2 |
| 2011 | Jiangsu | KT717777 | 11MC51_2011.10_XH | C4a2 |
| 2011 | Jiangsu | KT717778 | 11MA137_2011.10_TZ | C4a2 |
| 2011 | Jiangsu | KT717779 | 11MH41_2011.10_TZ | C4a2 |
| 2011 | Jiangsu | KT717780 | 11MC53_2011.11_XH | C4a2 |
| 2011 | Jiangsu | KT717781 | 11MD30_2011.11_TZ | C4a2 |
| 2011 | Jiangsu | KT717782 | 11ME25_2011.11_TZ | C4a2 |
| 2011 | Jiangsu | KT717783 | 11MA141_2011.11_TZ | C4a2 |
| 2011 | Jiangsu | KT717784 | 11MJ55_2011.11_TX | C4a2 |
| 2011 | Jiangsu | KT717785 | 11MC7_2011.02_XH | C4a2 |
| 2011 | Jiangsu | KT717786 | 11MA147_2011.12_TZ | C4a2 |
| 2011 | Jiangsu | KT717787 | 11MF73_2011.12_JJ | C4a2 |
| 2011 | Jiangsu | KT717788 | 11MF74_2011.12_JJ | C4a2 |
| 2011 | Jiangsu | KT717789 | 11MG46_2011.12_GG | C4a2 |
| 2011 | Jiangsu | KT717790 | 11MA140_2011.11_TZ | C4a2 |
| 2011 | Jiangsu | KT717791 | 11MH49_2011.12_TZ | C4a2 |
| 2011 | Jiangsu | KT717792 | 11MC54_2011.11_XH | C4a2 |
| 2011 | Jiangsu | KT717793 | 11MF69_2011.11_JJ | C4a2 |
| 2011 | Jiangsu | KT717794 | 11MJ51_2011.10_TX | C4a2 |
| 2011 | Fujian | KU595758 | EV71/FJFZ050/CHN/2011 | C4a2 |
| 2011 | Fujian | KU595759 | EV71/FJFZ053/CHN/2011 | C4a2 |
| 2011 | Fujian | KU595760 | EV71/FJFZ058/CHN/2011 | C4a2 |
| 2011 | Fujian | KU595761 | EV71/FJFZ066/CHN/2011 | C4a2 |
| 2011 | Fujian | KU595762 | EV71/FJXM010/CHN/2011 | C4a2 |
| 2011 | Fujian | KU595763 | EV71/FJXM028/CHN/2011 | C4a2 |
| 2011 | Fujian | KU595764 | EV71/FJXM033/CHN/2011 | C4a2 |
| 2011 | Fujian | KU595765 | EV71/FJPT004/CHN/2011 | C4a2 |
| 2011 | Fujian | KU595766 | EV71/FJPT005/CHN/2011 | C4a2 |
| 2011 | Fujian | KU595767 | EV71/FJSM004/CHN/2011 | C4a2 |
| 2011 | Fujian | KU595768 | EV71/FJQZ001/CHN/2011 | C4a2 |
| 2011 | Fujian | KU595769 | EV71/FJQZ009/CHN/2011 | C4a2 |
| 2011 | Fujian | KU595770 | EV71/FJQZ013/CHN/2011 | C4a2 |
| 2011 | Fujian | KU595771 | EV71/FJZZ024/CHN/2011 | C4a2 |
| 2011 | Fujian | KU595772 | EV71/FJNP023/CHN/2011 | C4a2 |
| 2011 | Fujian | KU595773 | EV71/FJLY005/CHN/2011 | C4a2 |
| 2011 | Fujian | KU595774 | EV71/FJLY010/CHN/2011 | C4a2 |
| 2011 | Fujian | KU595775 | EV71/FJLY011/CHN/2011 | C4a2 |
| 2011 | Fujian | KU595776 | EV71/FJLY016/CHN/2011 | C4a2 |
| 2011 | Fujian | KU595777 | EV71/FJND010/CHN/2011 | C4a2 |
| 2011 | Fujian | KU595778 | EV71/FJND015/CHN/2011 | C4a2 |
| 2011 | Fujian | KU595779 | EV71/FJND019/CHN/2011 | C4a2 |
| 2011 | Fujian | KU595780 | EV71/FJND056/CHN/2011 | C4a2 |
| 2011 | Fujian | KU595781 | EV71/FJNP026/CHN/2011 | C4a2 |
| 2011 | Fujian | KU595782 | EV71/FJFZ106/CHN/2011 | C4a2 |
| 2011 | Fujian | KU595783 | EV71/FJNP027/CHN/2011 | C4a2 |
| 2011 | Fujian | KU595784 | EV71/FJQZ034/CHN/2011 | C4a2 |
| 2011 | Fujian | KU595785 | EV71/FJQZ142/CHN/2011 | C4a2 |
| 2011 | Fujian | KU595786 | EV71/FJQZ101/CHN/2011 | C4a2 |
| 2011 | Fujian | KU595787 | EV71/FJSM013/CHN/2011 | C4a2 |
| 2011 | Hubei | KX791189 | wh71-CHN-11 | C4a2 |
| 2011 | Hubei | KX791190 | wh82-CHN-11 | C4a2 |
| 2011 | Hubei | KX791191 | wh92-CHN-11 | C4a2 |
| 2011 | Hubei | KX791192 | wh99-CHN-11 | C4a2 |
| 2011 | Hebei | KY081978 | SJZ2011-0002T/HeB/CHN/2011 | C4a2 |
| 2011 | Hebei | KY081979 | SJZ2011-0149F/HeB/CHN/2011 | C4a2 |
| 2011 | Yunnan | LC147338 | 201B-YN-CHN-2011 | C4a2 |
| 2011 | Yunnan | LC147339 | 205B-YN-CHN-2011 | C4a2 |
| 2011 | Yunnan | LC147340 | 210B-YN-CHN-2011 | C4a2 |
| 2011 | Yunnan | LC147341 | 223B-YN-CHN-2011 | C4a2 |
| 2011 | Yunnan | LC147342 | 229B-YN-CHN-2011 | C4a2 |
| 2011 | Yunnan | LC147343 | 232B-YN-CHN-2011 | C4a2 |
| 2011 | Yunnan | LC147344 | 233B-YN-CHN-2011 | C4a2 |
| 2011 | Yunnan | LC147345 | 236B-YN-CHN-2011 | C4a2 |
| 2011 | Yunnan | LC147346 | 239B-YN-CHN-2011 | C4a2 |
| 2011 | Yunnan | LC147347 | 241B-YN-CHN-2011 | C4a2 |
| 2011 | Yunnan | LC147348 | 242B-YN-CHN-2011 | C4a2 |
| 2011 | Yunnan | LC147349 | 244B-YN-CHN-2011 | C4a2 |
| 2011 | Yunnan | LC147350 | 260B-YN-CHN-2011 | C4a2 |
| 2011 | Yunnan | LC147351 | 262B-YN-CHN-2011 | C4a2 |
| 2011 | Yunnan | LC147352 | 264B-YN-CHN-2011 | C4a2 |
| 2011 | Yunnan | LC147353 | 273B-YN-CHN-2011 | C4a2 |
| 2011 | Yunnan | LC147354 | 275B-YN-CHN-2011 | C4a2 |
| 2011 | Yunnan | LC147355 | 276B-YN-CHN-2011 | C4a2 |
| 2011 | Yunnan | LC147356 | 277B-YN-CHN-2011 | C4a2 |
| 2011 | Yunnan | LC147357 | 278B-YN-CHN-2011 | C4a2 |
| 2011 | Yunnan | LC147358 | 281B-YN-CHN-2011 | C4a2 |
| 2011 | Yunnan | LC147359 | 282B-YN-CHN-2011 | C4a2 |
| 2011 | Yunnan | LC147360 | 284B-YN-CHN-2011 | C4a2 |
| 2011 | Yunnan | LC147361 | 285B-YN-CHN-2011 | C4a2 |
| 2012 | Jiangxi | JX427561 | 1201/NC/CHN/2012 | C4a2 |
| 2012 | Jiangxi | JX427562 | 1203/NC/CHN/2012 | C4a2 |
| 2012 | Jiangxi | KC007535 | NC406/NC/CHN/2012 | C4a2 |
| 2012 | Shanghai | KC570452 | SH12-036 | C4a2 |
| 2012 | Shanghai | KC570453 | SH12-276 | C4a2 |
| 2012 | Guangdong | KC801024 | GD-DG-310/2012 | C4a2 |
| 2012 | Guangdong | KC801025 | GD-QY-223/2012 | C4a2 |
| 2012 | Guangdong | KC801026 | GD-SZ-222/2012 | C4a2 |
| 2012 | Guangdong | KC801031 | GD-DG-210/2012 | C4a2 |
| 2012 | Guangdong | KC801032 | GD-SZ-186/2012 | C4a2 |
| 2012 | Guangdong | KC866778 | JB141230098 | C4a2 |
| 2012 | Guangdong | KC866779 | JB141230188 | C4a2 |
| 2012 | Guangdong | KC866780 | JB141230197 | C4a2 |
| 2012 | Guangdong | KC866781 | JB141230201 | C4a2 |
| 2012 | Guangdong | KC866782 | JB141230202 | C4a2 |
| 2012 | Guangdong | KC866783 | JB141230203 | C4a2 |
| 2012 | Guangdong | KC866784 | JB141230204 | C4a2 |
| 2012 | Guangdong | KC866785 | JB141230208 | C4a2 |
| 2012 | Guangdong | KC866786 | JB141230233 | C4a2 |
| 2012 | Guangdong | KC866787 | JB141230237 | C4a2 |
| 2012 | Guangdong | KC866788 | JB141230238 | C4a2 |
| 2012 | Guangdong | KC866789 | JB141230244 | C4a2 |
| 2012 | Guangdong | KC866790 | JB141230247 | C4a2 |
| 2012 | Guangdong | KC866791 | JB141230342 | C4a2 |
| 2012 | Guangdong | KC866792 | JB141230357 | C4a2 |
| 2012 | Guangdong | KC866793 | JB141230371 | C4a2 |
| 2012 | Guangdong | KC866794 | JB141210002 | C4a2 |
| 2012 | Guangdong | KC866795 | JB141210004 | C4a2 |
| 2012 | Guangdong | KC866796 | JB141210006 | C4a2 |
| 2012 | Guangdong | KC866797 | JB141210007 | C4a2 |
| 2012 | Guangdong | KC866798 | JB141210009 | C4a2 |
| 2012 | Guangdong | KC866799 | JB141210011 | C4a2 |
| 2012 | Guangdong | KC866800 | JB141210012 | C4a2 |
| 2012 | Guangdong | KC866801 | JB141210014 | C4a2 |
| 2012 | Guangdong | KC866802 | JB141210015 | C4a2 |
| 2012 | Guangdong | KC866803 | JB141210020 | C4a2 |
| 2012 | Guangdong | KC866804 | JB141210021 | C4a2 |
| 2012 | Guangdong | KC866805 | JB141210022 | C4a2 |
| 2012 | Guangdong | KC866806 | JB141210023 | C4a2 |
| 2012 | Guangdong | KC866807 | JB141210025 | C4a2 |
| 2012 | Guangdong | KC866808 | JB141210026 | C4a2 |
| 2012 | Guangdong | KC866809 | JB141210028 | C4a2 |
| 2012 | Guangdong | KC866810 | JB141210029 | C4a2 |
| 2012 | Guangdong | KC866811 | JB141210030 | C4a2 |
| 2012 | Guangdong | KC866812 | JB141210031 | C4a2 |
| 2012 | Guangdong | KC866813 | JB141210032 | C4a2 |
| 2012 | Guangdong | KC866814 | JB141210033 | C4a2 |
| 2012 | Guangdong | KC866815 | JB141210034 | C4a2 |
| 2012 | Zhejiang | KF358276 | EV71-Jiaxing-2 | C4a2 |
| 2012 | Jiangsu | KF826491 | TZ06 | C4a2 |
| 2012 | Anhui | KF925268 | FL002T/AH/CHN/2012 | C4a2 |
| 2012 | Anhui | KF925269 | FL003T/AH/CHN/2012 | C4a2 |
| 2012 | Anhui | KF925270 | FL012T/AH/CHN/2012 | C4a2 |
| 2012 | Anhui | KF925271 | FL028T/AH/CHN/2012 | C4a2 |
| 2012 | Anhui | KF925272 | FL031T/AH/CHN/2012 | C4a2 |
| 2012 | Liaoning | KF982854 | DL71 | C6 |
| 2012 | Beijing | KJ004554 | EV71/Hun12-14/2012 | C4a2 |
| 2012 | Beijing | KJ004555 | EV71/Hun12-10/2012 | C4a2 |
| 2012 | Shanghai | KJ139450 | SH/CHN/2012 | C4a2 |
| 2012 | Changsha | KJ156339 | CS591/HN/CHN/12 | C4a2 |
| 2012 | Changsha | KJ156340 | CS207/HN/CHN/12 | C4a2 |
| 2012 | Changsha | KJ156341 | CS688/HN/CHN/12 | C4a2 |
| 2012 | Changsha | KJ156342 | CS423/HN/CHN/12 | C4a2 |
| 2012 | Changsha | KJ156343 | CS487/HN/CHN/12 | C4a2 |
| 2012 | Hubei | KJ508182 | Hubei-HG/CHN/2012 | C4a2 |
| 2012 | Jilin | KJ508817 | A71 | C4a1 |
| 2012 | Shandong | KJ772433 | 12256/SD/CHN | C4a2 |
| 2012 | Shandong | KJ772434 | 12295/SD/CHN | C4a2 |
| 2012 | Zhejiang | KJ784496 | HuzhouE696/2012/CHN | C4a2 |
| 2012 | Changsha | KJ784497 | CS15/HN/CHN/12 | C4a2 |
| 2012 | Changsha | KJ784498 | CS98/HN/CHN/12 | C4a2 |
| 2012 | Changsha | KJ784499 | CS156/HN/CHN/12 | C4a2 |
| 2012 | Changsha | KJ784500 | CS731/HN/CHN/12 | C4a2 |
| 2012 | Guangdong | KJ865518 | 120465/GZ/2012/EV71 | C4a2 |
| 2012 | Guangdong | KJ865520 | 127583/GZ/2012/EV71 | C4a2 |
| 2012 | Guangdong | KJ865521 | 129157/GZ/2012/EV71 | C4a2 |
| 2012 | Guangdong | KJ865525 | 1213845/GZ/2012/EV71 | C4a2 |
| 2012 | Henan | KM211579 | Puyang-451-2012 | C4a2 |
| 2012 | Henan | KM211580 | Puyang-461-2012 | C4a2 |
| 2012 | Henan | KM260021 | 873-Henan-2012 | C4a2 |
| 2012 | Henan | KM260022 | 1571-Henan-2012 | C4a2 |
| 2012 | Henan | KM260023 | 1573-Henan-2012 | C4a2 |
| 2012 | Henan | KM260024 | 1576-Henan-2012 | C4a2 |
| 2012 | Jiangsu | KM408458 | 02Zhenjiang2012 | C4a2 |
| 2012 | Jiangsu | KM408459 | 03Zhenjiang2012 | C4a2 |
| 2012 | Jiangsu | KM408460 | 04Zhenjiang2012 | C4a2 |
| 2012 | Jiangsu | KM408461 | 05ZhenjiangZZ2012 | C4a2 |
| 2012 | Jiangsu | KM408462 | 06Zhenjiang2012 | C4a2 |
| 2012 | Shanghai | KM978832 | 207/SH/CHN/2012 | C4a2 |
| 2012 | Shanghai | KP137019 | Sh004/CHN/2012 | C4a2 |
| 2012 | Shanghai | KP137020 | Sh014/CHN/2012 | C4a2 |
| 2012 | Shanghai | KP137021 | Sh022/CHN/2012 | C4a2 |
| 2012 | Shanghai | KP137022 | Sh023/CHN/2012 | C4a2 |
| 2012 | Shanghai | KP137023 | Sh078/CHN/2012 | C4a2 |
| 2012 | Shanghai | KP137024 | Sh095/CHN/2012 | C4a2 |
| 2012 | Shanghai | KP137025 | Sh100/CHN/2012 | C4a2 |
| 2012 | Shanghai | KP137026 | Sh128/CHN/2012 | C4a2 |
| 2012 | Shanghai | KP137027 | Sh142/CHN/2012 | C4a2 |
| 2012 | Shanghai | KP137028 | Sh157/CHN/2012 | C4a2 |
| 2012 | Shanghai | KP137029 | Sh175/CHN/2012 | C4a2 |
| 2012 | Shanghai | KP137030 | Sh177/CHN/2012 | C4a2 |
| 2012 | Shanghai | KP137031 | Sh213/CHN/2012 | C4a2 |
| 2012 | Shanghai | KP137032 | Sh226/CHN/2012 | C4a2 |
| 2012 | Shanghai | KP137033 | Sh242/CHN/2012 | C4a2 |
| 2012 | Shanghai | KP137034 | Sh282/CHN/2012 | C4a2 |
| 2012 | Shanghai | KP137035 | Sh305/CHN/2012 | C4a2 |
| 2012 | Shanghai | KP137036 | Sh319/CHN/2012 | C4a2 |
| 2012 | Shanghai | KP137037 | Sh364/CHN/2012 | C4a2 |
| 2012 | Shanghai | KP137038 | Sh381/CHN/2012 | C4a2 |
| 2012 | Shanghai | KP137039 | Sh417/CHN/2012 | C4a2 |
| 2012 | Shanghai | KP192486 | Shp250/2012/China | C4a2 |
| 2012 | Shanghai | KP192487 | Shp263/2012/China | C4a2 |
| 2012 | Shanghai | KP192488 | Shp233/2012/China | C4a2 |
| 2012 | Shanghai | KP192489 | Shp348/2012/China | C4a2 |
| 2012 | Hubei | KP198623 | Hubei-WH/CHN/2012 | C4a2 |
| 2012 | Henan | KP198624 | Henan-ZMD/CHN/2012 | C4a2 |
| 2012 | Yunnan | KR779221 | YN2012-A039 | C4a2 |
| 2012 | Yunnan | KR779222 | YN2012-A076 | C4a2 |
| 2012 | Yunnan | KR779223 | YN2012-A083 | C4a2 |
| 2012 | Yunnan | KR779224 | YN2012-A096 | C4a2 |
| 2012 | Yunnan | KR779226 | YN2012-A006 | C4a2 |
| 2012 | Yunnan | KR779227 | YN2012-A017 | C4a2 |
| 2012 | Yunnan | KR779228 | YN2012-A025 | C4a2 |
| 2012 | Yunnan | KR779229 | YN2012-A072 | C4a2 |
| 2012 | Yunnan | KR779230 | YN2012-A087 | C4a2 |
| 2012 | Yunnan | KR779231 | YN2012-C001 | C4a2 |
| 2012 | Yunnan | KR782316 | YN2012-A002 | C4a2 |
| 2012 | Yunnan | KR782317 | YN2012-A011 | C4a2 |
| 2012 | Yunnan | KR782318 | YN2012-A013 | C4a2 |
| 2012 | Yunnan | KR782319 | YN2012-A016 | C4a2 |
| 2012 | Yunnan | KR782320 | YN2012-A019 | C4a2 |
| 2012 | Yunnan | KR782321 | YN2012-A033 | C4a2 |
| 2012 | Yunnan | KR782322 | YN2012-A035 | C4a2 |
| 2012 | Yunnan | KR782323 | YN2012-A043 | C4a2 |
| 2012 | Yunnan | KR782324 | YN2012-A048 | C4a2 |
| 2012 | Yunnan | KR782325 | YN2012-A049 | C4a2 |
| 2012 | Yunnan | KR782326 | YN2012-A052 | C4a2 |
| 2012 | Yunnan | KR782327 | YN2012-A053 | C4a2 |
| 2012 | Yunnan | KR782328 | YN2012-A057 | C4a2 |
| 2012 | Yunnan | KR782329 | YN2012-A058 | C4a2 |
| 2012 | Yunnan | KR782330 | YN2012-A059 | C4a2 |
| 2012 | Yunnan | KR782331 | YN2012-A060 | C4a2 |
| 2012 | Yunnan | KR782332 | YN2012-A079 | C4a2 |
| 2012 | Yunnan | KR782333 | YN2012-A088 | C4a2 |
| 2012 | Yunnan | KR782334 | YN2012-A094 | C4a2 |
| 2012 | Yunnan | KR782335 | YN2012-A098 | C4a2 |
| 2012 | Yunnan | KR782336 | YN2012-A099 | C4a2 |
| 2012 | Yunnan | KR782337 | YN2012-C002 | C4a2 |
| 2012 | Yunnan | KR782338 | YN2012-C005 | C4a2 |
| 2012 | Yunnan | KR782339 | YN2012-C006 | C4a2 |
| 2012 | Yunnan | KR782340 | YN2012-C007 | C4a2 |
| 2012 | Yunnan | KR782341 | YN2012-C010 | C4a2 |
| 2012 | Yunnan | KR782342 | YN2012-C011 | C4a2 |
| 2012 | Yunnan | KR782343 | YN2012-C023 | C4a2 |
| 2012 | Yunnan | KR782344 | YN2012-C043 | C4a2 |
| 2012 | Yunnan | KR782345 | YN2012-C044 | C4a2 |
| 2012 | Yunnan | KR782346 | YN2012-C046 | C4a2 |
| 2012 | Yunnan | KR782347 | YN2012-C075 | C4a2 |
| 2012 | Yunnan | KR782348 | YN2012-C083 | C4a2 |
| 2012 | Yunnan | KR782349 | YN2012-C087 | C4a2 |
| 2012 | Yunnan | KR782350 | YN2012-C089 | C4a2 |
| 2012 | Yunnan | KR782351 | YN2012-C104 | C4a2 |
| 2012 | Yunnan | KR782352 | YN2012-C113 | C4a2 |
| 2012 | Yunnan | KR782353 | YN2012-C132 | C4a2 |
| 2012 | Yunnan | KR782354 | YN2012-C133 | C4a2 |
| 2012 | Jiangsu | KT327133 | 524/SZ/CHN/2012 | C4a2 |
| 2012 | Jiangsu | KT327137 | 531/SZ/CHN/2012 | C4a2 |
| 2012 | Jiangsu | KT327144 | 526/SZ/CHN/2012 | C4a2 |
| 2012 | Jiangsu | KT717795 | 12ME1_2012.01_TZ | C4a2 |
| 2012 | Jiangsu | KT717796 | 12MG1_2012.01_GG | C4a2 |
| 2012 | Jiangsu | KT717797 | 12MC3_2012.01_XH | C4a2 |
| 2012 | Jiangsu | KT717798 | 12MJ2_2012.01_TX | C4a2 |
| 2012 | Jiangsu | KT717799 | 12MA155_2012.02_TZ | C4a2 |
| 2012 | Jiangsu | KT717800 | 12MF6_2012.02_JJ | C4a2 |
| 2012 | Jiangsu | KT717801 | 12MF8_2012.02_JJ | C4a2 |
| 2012 | Jiangsu | KT717802 | 12MA156_2012.03_TZ | C4a2 |
| 2012 | Jiangsu | KT717803 | 12MA157_2012.03_TZ | C4a2 |
| 2012 | Jiangsu | KT717804 | 12MA158_2012.03_TZ | C4a2 |
| 2012 | Jiangsu | KT717805 | 12MA160_2012.03_TZ | C4a2 |
| 2012 | Jiangsu | KT717806 | 12MA159_2012.03_TZ | C4a2 |
| 2012 | Jiangsu | KT717807 | 12MF16_2012.03_JJ | C4a2 |
| 2012 | Jiangsu | KT717808 | 12MG3_2012.01_GG | C4a2 |
| 2012 | Jiangsu | KT717809 | 12MA152_2012.02_TZ | C4a2 |
| 2012 | Jiangsu | KT717810 | 12MC20_2012.04_XH | C4a2 |
| 2012 | Jiangsu | KT717811 | 12MM14_2012.04_JY | C4a2 |
| 2012 | Jiangsu | KT717812 | 12MA164_2012.04_TZ | C4a2 |
| 2012 | Jiangsu | KT717813 | 12MA166_2012.04_TZ | C4a2 |
| 2012 | Jiangsu | KT717814 | 12MG12_2012.04_GG | C4a2 |
| 2012 | Jiangsu | KT717815 | 12MC18_2012.04_XH | C4a2 |
| 2012 | Jiangsu | KT717816 | 12MC19_2012.04_XH | C4a2 |
| 2012 | Jiangsu | KT717817 | 12MF20_2012.04_JJ | C4a2 |
| 2012 | Jiangsu | KT717818 | 12MJ18_2012.04_TX | C4a2 |
| 2012 | Jiangsu | KT717819 | 12MH56_2012.04_TZ | C4a2 |
| 2012 | Jiangsu | KT717820 | 12MH57_2012.04_TZ | C4a2 |
| 2012 | Jiangsu | KT717821 | 12MD22_2012.05_TZ | C4a2 |
| 2012 | Jiangsu | KT717822 | 12MD32_2012.06_TZ | C4a2 |
| 2012 | Jiangsu | KT717823 | 12MA169_2012.05_TZ | C4a2 |
| 2012 | Jiangsu | KT717824 | 12MA171_2012.05_TZ | C4a2 |
| 2012 | Jiangsu | KT717825 | 12ME08_2012.05_TZ | C4a2 |
| 2012 | Jiangsu | KT717826 | 12MD19_2012.05_TZ | C4a2 |
| 2012 | Jiangsu | KT717827 | 12MD26_2012.05_TZ | C4a2 |
| 2012 | Jiangsu | KT717828 | 12MC22_2012.05_XH | C4a2 |
| 2012 | Jiangsu | KT717829 | 12MC25_2012.05_XH | C4a2 |
| 2012 | Jiangsu | KT717830 | 12MA176_2012.06_TZ | C4a2 |
| 2012 | Jiangsu | KT717831 | 12ME09_2012.06_TZ | C4a2 |
| 2012 | Jiangsu | KT717832 | 12MC30_2012.06_XH | C4a2 |
| 2012 | Jiangsu | KT717833 | 12MM23_2012.06_JY | C4a2 |
| 2012 | Jiangsu | KT717834 | 12MG20_2012.06_GG | C4a2 |
| 2012 | Jiangsu | KT717835 | 12MC28_2012.06_XH | C4a2 |
| 2012 | Jiangsu | KT717836 | 12MH66_2012.07_TZ | C4a2 |
| 2012 | Jiangsu | KT717837 | 12MC33_2012.07_XH | C4a2 |
| 2012 | Jiangsu | KT717838 | 12MC35_2012.07_XH | C4a2 |
| 2012 | Jiangsu | KT717839 | 12MG23_2012.07_GG | C4a2 |
| 2012 | Jiangsu | KT717840 | 12MG26_2012.07_GG | C4a2 |
| 2012 | Jiangsu | KT717841 | 12ME12_2012.07_TZ | C4a2 |
| 2012 | Jiangsu | KT717842 | 12MH69_2012.08_TZ | C4a2 |
| 2012 | Jiangsu | KT717843 | 12MG28_2012.08_GG | C4a2 |
| 2012 | Jiangsu | KT717844 | 12MJ41_2012.09_TX | B5 |
| 2012 | Jiangsu | KT717845 | 12MG31_2012.10_GG | C4a2 |
| 2012 | Jiangsu | KT717846 | 12MC39_2012.08_XH | C4a2 |
| 2012 | Jiangsu | KT717847 | 12MG33_2012.10_GG | C4a2 |
| 2012 | Jiangsu | KT717848 | 12MH72_2012.10_TZ | C4a2 |
| 2012 | Jiangsu | KT717849 | 12MG37_2012.11_GG | C4a2 |
| 2012 | Jiangsu | KT717850 | 12MG36_2012.11_GG | C4a2 |
| 2012 | Jiangsu | KT717851 | 12MF80_2012.12_JJ | C4a2 |
| 2012 | Jiangsu | KT717852 | 12MJ56_2012.12_TX | C4a2 |
| 2012 | Jiangsu | KT717853 | 12MJ59_2012.12_TX | C4a2 |
| 2012 | Jiangsu | KT717854 | 12MJ60_2012.12_TX | C4a2 |
| 2012 | Jiangsu | KT717855 | 12MC36_2012.08_XH | C4a2 |
| 2012 | Jiangsu | KT717856 | 13MF12_2013.03_JJ | C4a2 |
| 2012 | Guangdong | KU578264 | 05/SHENZHEN/12/China/HFMD severe/2012 | C4a2 |
| 2012 | Guangdong | KU578265 | 08/SHENZHEN/12/China/HFMD fatal/2012 | C4a2 |
| 2012 | Guangdong | KU578266 | 09/SHENZHEN/12/China/HFMD severe/2012 | C4a2 |
| 2012 | Guangdong | KU578267 | 12/SHENZHEN/12/China/HFMD severe/2012 | C4a2 |
| 2012 | Guangdong | KU578268 | 13/SHENZHEN/12/China/HFMD severe/2012 | C4a2 |
| 2012 | Guangdong | KU578269 | 228/SHENZHEN/12/China/HFMD mild/2012 | C4a2 |
| 2012 | Guangdong | KU578270 | 230/SHENZHEN/12/China/HFMD mild/2012 | C4a2 |
| 2012 | Guangdong | KU578271 | 541/SHENZHEN/12/China/HFMD mild/2012 | C4a2 |
| 2012 | Guangdong | KU578272 | 436/SHENZHEN/12/China/HFMD mild/2012 | C4a2 |
| 2012 | Guangdong | KU578273 | 220/SHENZHEN/12/China/HFMD mild/2012 | C4a2 |
| 2012 | Fujian | KU595788 | EV71/FJFZ001/CHN/2012 | C4a2 |
| 2012 | Fujian | KU595789 | EV71/FJFZ011/CHN/2012 | C4a2 |
| 2012 | Fujian | KU595790 | EV71/FJFZ013/CHN/2012 | C4a2 |
| 2012 | Fujian | KU595791 | EV71/FJFZ059/CHN/2012 | C4a2 |
| 2012 | Fujian | KU595792 | EV71/FJFZ097/CHN/2012 | C4a2 |
| 2012 | Fujian | KU595793 | EV71/FJXM032/CHN/2012 | C4a2 |
| 2012 | Fujian | KU595794 | EV71/FJXM033/CHN/2012 | C4a2 |
| 2012 | Fujian | KU595795 | EV71/FJXM048/CHN/2012 | C4a2 |
| 2012 | Fujian | KU595796 | EV71/FJXM051/CHN/2012 | C4a2 |
| 2012 | Fujian | KU595797 | EV71/FJPT040/CHN/2012 | C4a2 |
| 2012 | Fujian | KU595798 | EV71/FJSM003/CHN/2012 | C4a2 |
| 2012 | Fujian | KU595799 | EV71/FJSM005/CHN/2012 | C4a2 |
| 2012 | Fujian | KU595800 | EV71/FJSM009/CHN/2012 | C4a2 |
| 2012 | Fujian | KU595801 | EV71/FJSM032/CHN/2012 | C4a2 |
| 2012 | Fujian | KU595802 | EV71/FJSM057/CHN/2012 | C4a2 |
| 2012 | Fujian | KU595803 | EV71/FJSM062/CHN/2012 | C4a2 |
| 2012 | Fujian | KU595804 | EV71/FJQZ001/CHN/2012 | C4a2 |
| 2012 | Fujian | KU595805 | EV71/FJQZ002/CHN/2012 | C4a2 |
| 2012 | Fujian | KU595806 | EV71/FJQZ003/CHN/2012 | C4a2 |
| 2012 | Fujian | KU595807 | EV71/FJQZ005/CHN/2012 | C4a2 |
| 2012 | Fujian | KU595808 | EV71/FJQZ011/CHN/2012 | C4a2 |
| 2012 | Fujian | KU595809 | EV71/FJQZ015/CHN/2012 | C4a2 |
| 2012 | Fujian | KU595810 | EV71/FJQZ067/CHN/2012 | C4a2 |
| 2012 | Fujian | KU595811 | EV71/FJQZ103/CHN/2012 | C4a2 |
| 2012 | Fujian | KU595812 | EV71/FJZZ001/CHN/2012 | C4a2 |
| 2012 | Fujian | KU595813 | EV71/FJZZ002/CHN/2012 | C4a2 |
| 2012 | Fujian | KU595814 | EV71/FJNP002/CHN/2012 | C4a2 |
| 2012 | Fujian | KU595815 | EV71/FJNP007/CHN/2012 | C4a2 |
| 2012 | Fujian | KU595816 | EV71/FJNP013/CHN/2012 | C4a2 |
| 2012 | Fujian | KU595817 | EV71/FJNP017/CHN/2012 | C4a2 |
| 2012 | Fujian | KU595818 | EV71/FJLY002/CHN/2012 | C4a2 |
| 2012 | Fujian | KU595819 | EV71/FJLY004/CHN/2012 | C4a2 |
| 2012 | Fujian | KU595820 | EV71/FJLY008/CHN/2012 | C4a2 |
| 2012 | Fujian | KU595821 | EV71/FJLY012/CHN/2012 | C4a2 |
| 2012 | Fujian | KU595822 | EV71/FJND003/CHN/2012 | C4a2 |
| 2012 | Fujian | KU595823 | EV71/FJND011/CHN/2012 | C4a2 |
| 2012 | Fujian | KU595824 | EV71/FJND015/CHN/2012 | C4a2 |
| 2012 | Fujian | KU595825 | EV71/FJND017/CHN/2012 | C4a2 |
| 2012 | Fujian | KU595826 | EV71/FJPTN002/CHN/2012 | C4a2 |
| 2012 | Fujian | KU595827 | EV71/FJPTN005/CHN/2012 | C4a2 |
| 2012 | Fujian | KU595828 | EV71/FJPT011/CHN/2012 | C4a2 |
| 2012 | Fujian | KU595829 | EV71/FJZZ008/CHN/2012 | C4a2 |
| 2012 | Fujian | KU595830 | EV71/FJPT013/CHN/2012 | C4a2 |
| 2012 | Hubei | KX791193 | wh170-CHN-12 | C4a2 |
| 2012 | Hubei | KX791194 | wh205-CHN-12 | C4a2 |
| 2012 | Hubei | KX791195 | wh207-CHN-12 | C4a2 |
| 2012 | Shanghai | KX871480 | SHEV2012-010 | C4a2 |
| 2012 | Shanghai | KX871481 | SHEV2012-013 | C4a2 |
| 2012 | Shanghai | KX871482 | SHEV2012-017 | C4a2 |
| 2012 | Shanghai | KX871483 | SHEV2012-018 | C4a2 |
| 2012 | Shanghai | KX871484 | SHEV2012-026 | C4a2 |
| 2012 | Shanghai | KX871485 | SHEV2012-032 | C4a2 |
| 2012 | Shanghai | KX871486 | SHEV2012-036 | C4a2 |
| 2012 | Shanghai | KX871487 | SHEV2012-038 | C4a2 |
| 2012 | Shanghai | KX871488 | SHEV2012-039 | C4a2 |
| 2012 | Shanghai | KX871489 | SHEV2012-040 | C4a2 |
| 2012 | Shanghai | KX871490 | SHEV2012-042 | C4a2 |
| 2012 | Shanghai | KX871491 | SHEV2012-060 | C4a2 |
| 2012 | Shanghai | KX871492 | SHEV2012-E3 | C4a2 |
| 2012 | Shanghai | KX871493 | SHEV2012-E4 | C4a2 |
| 2012 | Shanghai | KX871494 | SHEV2012-E5 | C4a2 |
| 2012 | Shanghai | KX871495 | SHEV2012-JW1 | C4a2 |
| 2012 | Shanghai | KX871496 | SHEV2012-JW10 | C4a2 |
| 2012 | Shanghai | KX871497 | SHEV2012-JW11 | C4a2 |
| 2012 | Shanghai | KX871498 | SHEV2012-JW12 | C4a2 |
| 2012 | Shanghai | KX871499 | SHEV2012-JW13 | C4a2 |
| 2012 | Shanghai | KX871500 | SHEV2012-JW14 | C4a2 |
| 2012 | Shanghai | KX871501 | SHEV2012-JW2 | C4a2 |
| 2012 | Shanghai | KX871502 | SHEV2012-JW3 | C4a2 |
| 2012 | Shanghai | KX871503 | SHEV2012-JW6 | C4a2 |
| 2012 | Shanghai | KX871504 | SHEV2012-JW7 | C4a2 |
| 2012 | Shanghai | KX871505 | SHEV2012-JW8 | C4a2 |
| 2012 | Shanghai | KX871673 | SHEV2012-033 | C4a2 |
| 2012 | Shanghai | KX871674 | SHEV2012-059 | C4a2 |
| 2012 | Hebei | KY081980 | SJZ2012-0885T/HeB/CHN/2012 | C4a2 |
| 2012 | Hebei | KY081981 | SJZ2012-0985T/HeB/CHN/2012 | C4a2 |
| 2012 | Hebei | KY081982 | SJZ2012-1022T/HeB/CHN/2012 | C4a2 |
| 2012 | Guangxi | MF185262 | 7146_GX_2012 | C4a2 |
| 2012 | Guangxi | MF185263 | 208-1_GX_2012 | C4a2 |
| 2012 | Guangxi | MF185264 | 218-1_GX_2012 | C4a2 |
| 2012 | Guangxi | MF185265 | 160-5_GX_2012 | C4a2 |
| 2012 | Guangxi | MF185266 | 270-1_GX_2012 | C4a2 |
| 2012 | Guangxi | MF185268 | 207-3_GX_2012 | C4a2 |
| 2012 | Guangxi | MF185269 | 190-3_GX_2012 | C4a2 |
| 2012 | Guangxi | MF185270 | 171-1_GX_2012 | C4a2 |
| 2012 | Guangxi | MF185271 | 162-1_GX_2012 | C4a2 |
| 2012 | Guangxi | MF185272 | 124-3_GX_2012 | C4a2 |
| 2012 | Guangxi | MF185273 | 124-1_GX_2012 | C4a2 |
| 2012 | Guangxi | MF185274 | 113-1_GX_2012 | C4a2 |
| 2012 | Guangxi | MF185275 | 110-5_GX_2012 | C4a2 |
| 2012 | Guangxi | MF185276 | 110-2_GX_2012 | C4a2 |
| 2012 | Guangxi | MF185277 | 110-1_GX_2012 | C4a2 |
| 2012 | Guangxi | MF185278 | 098-4_GX_2012 | C4a2 |
| 2012 | Guangxi | MF185279 | 098-1_GX_2012 | C4a2 |
| 2012 | Guangxi | MF185280 | 058-1_GX_2012 | C4a2 |
| 2012 | Guangxi | MF185281 | 058-3_GX_2012 | C4a2 |
| 2012 | Guangxi | MF185282 | 059-1_GX_2012 | C4a2 |
| 2012 | Guangxi | MF185283 | 059-2_GX_2012 | C4a2 |
| 2012 | Guangxi | MF185284 | 084-1_GX_2012 | C4a2 |
| 2012 | Guangxi | MF185285 | 084-2_GX_2012 | C4a2 |
| 2012 | Guangxi | MF185286 | 086-1_GX_2012 | C4a2 |
| 2012 | Guangxi | MF185287 | 093-1_GX_2012 | C4a2 |
| 2012 | Guangxi | MF185288 | 093-3_GX_2012 | C4a2 |
| 2012 | Guangxi | MF185289 | 093-4_GX_2012 | C4a2 |
| 2012 | Guangxi | MF185290 | 7118_GX_2012 | C4a2 |
| 2013 | Hunan | KF142411 | HNCZ/201203 | C4a2 |
| 2013 | Hunan | KF142412 | HNCZ/201208 | C4a2 |
| 2013 | Hunan | KF142413 | HNCZ/201211 | C4a2 |
| 2013 | Hunan | KF142414 | HNCZ-2012-15 | C4a2 |
| 2013 | Hunan | KF142415 | HNCZ-2012-07 | C4a2 |
| 2013 | Hunan | KF142416 | HNCZ-2012-18 | C4a2 |
| 2013 | Hunan | KF142417 | HNCZ-2012-01 | C4a2 |
| 2013 | Hunan | KF142418 | HNCZ-2012-09 | C4a2 |
| 2013 | Hunan | KF142419 | HNCZ-2012-13 | C4a2 |
| 2013 | Hunan | KF142420 | HNCZ-2012-17 | C4a2 |
| 2013 | Shandong | KJ772435 | 13004/SD/CHN | C4a2 |
| 2013 | Shandong | KJ772436 | 13061/SD/CHN | C4a2 |
| 2013 | Shandong | KJ772437 | 13112/SD/CHN | C4a2 |
| 2013 | Shandong | KJ772438 | 13138/SD/CHN | C4a2 |
| 2013 | Shandong | KJ772439 | 13164/SD/CHN | C4a2 |
| 2013 | Shandong | KJ772440 | 13168/SD/CHN | C4a2 |
| 2013 | Shandong | KJ772441 | 13175/SD/CHN | C4a2 |
| 2013 | Shandong | KJ772442 | 13196/SD/CHN | C4a2 |
| 2013 | Shandong | KJ772443 | 13390/SD/CHN | C4a2 |
| 2013 | Shandong | KJ772444 | 13411/SD/CHN | C4a2 |
| 2013 | Guangdong | KJ865522 | 135520/GZ/2013/EV71 | C4a2 |
| 2013 | Guangdong | KJ865526 | 1311423/GZ/2013/EV71 | C4a2 |
| 2013 | Guangdong | KJ865527 | 1320703/GZ/2013/EV71 | C4a2 |
| 2013 | Guangdong | KJ865528 | 1322103/GZ/2013/EV71 | C4a2 |
| 2013 | Guangdong | KJ865529 | 1328233/GZ/2013/EV71 | C4a2 |
| 2013 | Henan | KM260025 | 82-Henan-2013 | C4a2 |
| 2013 | Henan | KM260026 | 83-Henan-2013 | C4a2 |
| 2013 | Henan | KM260027 | 156-Henan-2013 | C4a2 |
| 2013 | Henan | KM260028 | 157-Henan-2013 | C4a2 |
| 2013 | Henan | KM260029 | 212-Henan-2013 | C4a2 |
| 2013 | Henan | KM260030 | 229-Henan-2013 | C4a2 |
| 2013 | Henan | KM260031 | 355-Henan-2013 | C4a2 |
| 2013 | Henan | KM260032 | 473-Henan-2013 | C4a2 |
| 2013 | Henan | KM260033 | 762-Henan-2013 | C4a2 |
| 2013 | Henan | KM260034 | 917-Henan-2013 | C4a2 |
| 2013 | Jiangsu | KM408463 | 01Zhenjiang2013 | C4a2 |
| 2013 | Jiangsu | KM408464 | 02Zhenjiang2013 | C4a2 |
| 2013 | Jiangsu | KM408465 | 03Zhenjiang2013 | C4a2 |
| 2013 | Jiangsu | KM408466 | 04Zhenjiang2013 | C4a2 |
| 2013 | Jiangsu | KM408467 | 05Zhenjiang2013 | C4a2 |
| 2013 | Jiangsu | KM408468 | 06Zhenjiang2013 | C4a2 |
| 2013 | Guangdong | KP005789 | JB141310002-EV71 | C4a2 |
| 2013 | Guangdong | KP005790 | JB141310003-EV71 | C4a2 |
| 2013 | Guangdong | KP005791 | JB141310006-EV71 | C4a2 |
| 2013 | Guangdong | KP005792 | JB141310007-EV71 | C4a2 |
| 2013 | Guangdong | KP005793 | JB141310008-EV71 | C4a2 |
| 2013 | Guangdong | KP005794 | JB141310009-EV71 | C4a2 |
| 2013 | Guangdong | KP005795 | JB141310011-EV71 | C4a2 |
| 2013 | Guangdong | KP005796 | JB141310012-EV71 | C4a2 |
| 2013 | Guangdong | KP005797 | JB141310013-EV71 | C4a2 |
| 2013 | Guangdong | KP005798 | JB141310014-EV71 | C4a2 |
| 2013 | Guangdong | KP005799 | JB141330004-EV71 | C4a2 |
| 2013 | Guangdong | KP005800 | JB141330007-EV71 | C4a2 |
| 2013 | Guangdong | KP005801 | JB141330010-EV71 | C4a2 |
| 2013 | Guangdong | KP005802 | JB141330012-EV71 | C4a2 |
| 2013 | Guangdong | KP005803 | JB141330013-EV71 | C4a2 |
| 2013 | Guangdong | KP005804 | JB141330023-EV71 | C4a2 |
| 2013 | Guangdong | KP005805 | JB141330026-EV71 | C4a2 |
| 2013 | Guangdong | KP005806 | JB141330029-EV71 | C4a2 |
| 2013 | Guangdong | KP005807 | JB141330033-EV71 | C4a2 |
| 2013 | Guangdong | KP005808 | JB141330034-EV71 | C4a2 |
| 2013 | Guangdong | KP005809 | JB141330036-EV71 | C4a2 |
| 2013 | Guangdong | KP005810 | JB141330037-EV71 | C4a2 |
| 2013 | Guangdong | KP005811 | JB141330048-EV71 | C4a2 |
| 2013 | Guangdong | KP005812 | JB14133049-EV71 | C4a2 |
| 2013 | Guangdong | KP005813 | JB14133059-EV71 | C4a2 |
| 2013 | Guangdong | KP005814 | JB141330066-EV71 | C4a2 |
| 2013 | Guangdong | KP005815 | JB141330073-EV71 | C4a2 |
| 2013 | Guangdong | KP005816 | JB141330075-EV71 | C4a2 |
| 2013 | Guangdong | KP005817 | JB141330085-EV71 | C4a2 |
| 2013 | Guangdong | KP005818 | JB141330092-EV71 | C4a2 |
| 2013 | Guangdong | KP005819 | JB141330094-EV71 | C4a2 |
| 2013 | Guangdong | KP005820 | JB141330105-EV71 | C4a2 |
| 2013 | Guangdong | KP005821 | JB141330108-EV71 | C4a2 |
| 2013 | Guangdong | KP005822 | JB141330135-EV71 | C4a2 |
| 2013 | Guangdong | KP005823 | JB141330150-EV71 | C4a2 |
| 2013 | Guangdong | KP005824 | JB141330193-EV71 | C4a2 |
| 2013 | Guangdong | KP005825 | JB141330220-EV71 | C4a2 |
| 2013 | Guangdong | KP005826 | JB141330225-EV71 | C4a2 |
| 2013 | Guangdong | KP005827 | JB141330229-EV71 | C4a2 |
| 2013 | Guangdong | KP005828 | JB141330230-EV71 | C4a2 |
| 2013 | Guangdong | KP005829 | JB141330231-EV71 | C4a2 |
| 2013 | Guangdong | KP005830 | JB141330232-EV71 | C4a2 |
| 2013 | Guangdong | KP005831 | JB141330302-EV71 | C4a2 |
| 2013 | Guangdong | KP005832 | JB141330303-EV71 | C4a2 |
| 2013 | Guangdong | KP005833 | JB141330305-EV71 | C4a2 |
| 2013 | Guangdong | KP005834 | JB141330327-EV71 | C4a2 |
| 2013 | Beijing | KP177477 | BJ13018/CHN/13 | C4a2 |
| 2013 | Beijing | KP177478 | BJ13033/CHN/13 | C4a2 |
| 2013 | Beijing | KP177479 | BJ13037/CHN/13 | C4a2 |
| 2013 | Beijing | KP177480 | BJ13038/CHN/13 | C4a2 |
| 2013 | Beijing | KP177481 | BJ13041/CHN/13 | C4a2 |
| 2013 | Beijing | KP177482 | BJ13050/CHN/13 | C4a2 |
| 2013 | Beijing | KP177483 | BJ13056/CHN/13 | C4a2 |
| 2013 | Beijing | KP177484 | BJ13057/CHN/13 | C4a2 |
| 2013 | Beijing | KP177485 | BJ13058/CHN/13 | C4a2 |
| 2013 | Beijing | KP177486 | BJ13062/CHN/13 | C4a2 |
| 2013 | Beijing | KP177487 | BJ13069/CHN/13 | C4a2 |
| 2013 | Beijing | KP177488 | BJ13097/CHN/13 | C4a2 |
| 2013 | Beijing | KP177489 | BJ13106/CHN/13 | C4a2 |
| 2013 | Beijing | KP177490 | BJ13114/CHN/13 | C4a2 |
| 2013 | Beijing | KP177491 | BJ13116/CHN/13 | C4a2 |
| 2013 | Beijing | KP177492 | BJ13120/CHN/13 | C4a2 |
| 2013 | Beijing | KP177493 | BJ13127/CHN/13 | C4a2 |
| 2013 | Beijing | KP177494 | BJ13128/CHN/13 | C4a2 |
| 2013 | Beijing | KP177495 | BJ13165/CHN/13 | C4a2 |
| 2013 | Beijing | KP177496 | BJ13166/CHN/13 | C4a2 |
| 2013 | Beijing | KP177497 | BJ13171/CHN/13 | C4a2 |
| 2013 | Beijing | KP177498 | BJ13192/CHN/13 | C4a2 |
| 2013 | Beijing | KP177499 | BJ13212/CHN/13 | C4a2 |
| 2013 | Beijing | KP177500 | BJ13217/CHN/13 | C4a2 |
| 2013 | Beijing | KP177501 | BJ13227/CHN/13 | C4a2 |
| 2013 | Beijing | KP177502 | BJ13298/CHN/13 | C4a2 |
| 2013 | Beijing | KP177503 | BJ13394/CHN/13 | C4a2 |
| 2013 | Beijing | KP177504 | BJ13420/CHN/13 | C4a2 |
| 2013 | Beijing | KP289417 | EV71/P1027/2013/China | C4a2 |
| 2013 | Beijing | KP289418 | EV71/P1031/2013/China | C4a2 |
| 2013 | Beijing | KP289419 | EV71/P1034/2013/China | C4a2 |
| 2013 | Beijing | KP289420 | EV71/P123/2013/China | C4a2 |
| 2013 | Beijing | KP289421 | EV71/P156/2013/China | C4a2 |
| 2013 | Beijing | KP289422 | EV71/P16/2013/China | C4a2 |
| 2013 | Beijing | KP289423 | EV71/P222/2013/China | C4a2 |
| 2013 | Beijing | KP289424 | EV71/P267/2013/China | C4a2 |
| 2013 | Beijing | KP289425 | EV71/P352/2013/China | C4a2 |
| 2013 | Beijing | KP289426 | EV71/P40/2013/China | C4a2 |
| 2013 | Beijing | KP289427 | EV71/P454/2013/China | C4a2 |
| 2013 | Beijing | KP289428 | EV71/P63/2013/China | C4a2 |
| 2013 | Beijing | KP289429 | EV71/P654/2013/China | C4a2 |
| 2013 | Beijing | KP289430 | EV71/P868/2013/China | C4a2 |
| 2013 | Beijing | KP289431 | EV71/P977/2013/China | C4a2 |
| 2013 | Beijing | KP289432 | EV71/P990/2013/China | C4a2 |
| 2013 | Yunnan | KR779225 | YN2013-A001 | C4a2 |
| 2013 | Yunnan | KR782355 | YN2013-A005 | C4a2 |
| 2013 | Yunnan | KR782356 | YN2013-A019 | C4a2 |
| 2013 | Yunnan | KR782357 | YN2013-A066 | C4a2 |
| 2013 | Yunnan | KR782358 | YN2013-A078 | C4a2 |
| 2013 | Yunnan | KR782359 | YN2013-A093 | C4a2 |
| 2013 | Yunnan | KR782360 | YN2013-A106 | C4a2 |
| 2013 | Yunnan | KR782361 | YN2013-A121 | C4a2 |
| 2013 | Yunnan | KR782362 | YN2013-A125 | C4a2 |
| 2013 | Yunnan | KR782363 | YN2013-A127 | C4a2 |
| 2013 | Yunnan | KR782364 | YN2013-A129 | C4a2 |
| 2013 | Yunnan | KR782365 | YN2013-A137 | C4a2 |
| 2013 | Yunnan | KR782366 | YN2013-A138 | C4a2 |
| 2013 | Yunnan | KR782367 | YN2013-A161 | C4a2 |
| 2013 | Yunnan | KR782368 | YN2013-A162 | C4a2 |
| 2013 | Yunnan | KR782369 | YN2013-A186 | C4a2 |
| 2013 | Yunnan | KR782370 | YN2013-A188 | C4a2 |
| 2013 | Yunnan | KR782371 | YN2013-A207 | C4a2 |
| 2013 | Yunnan | KR782372 | YN2013-C016 | C4a2 |
| 2013 | Yunnan | KR782373 | YN2013-C017 | C4a2 |
| 2013 | Yunnan | KR782374 | YN2013-C020 | C4a2 |
| 2013 | Yunnan | KR782375 | YN2013-C103 | C4a2 |
| 2013 | Yunnan | KR782376 | YN2013-C105 | C4a2 |
| 2013 | Yunnan | KR782377 | YN2013-C111 | C4a2 |
| 2013 | Yunnan | KR782378 | YN2013-C115 | C4a2 |
| 2013 | Yunnan | KR782379 | YN2013-C119 | C4a2 |
| 2013 | Yunnan | KR782380 | YN2013-C120 | C4a2 |
| 2013 | Yunnan | KR782381 | YN2013-C203 | C4a2 |
| 2013 | Yunnan | KR782382 | YN2013-C210 | C4a2 |
| 2013 | Yunnan | KR782383 | YN2013-C216 | C4a2 |
| 2013 | Yunnan | KR782384 | YN2013-C218 | C4a2 |
| 2013 | Yunnan | KR782385 | YN2013-A015 | C4a2 |
| 2013 | Yunnan | KR782386 | YN2013-A051 | C4a2 |
| 2013 | Yunnan | KR782387 | YN2013-A052 | C4a2 |
| 2013 | Yunnan | KR782388 | YN2013-A053 | C4a2 |
| 2013 | Yunnan | KR782389 | YN2013-A054 | C4a2 |
| 2013 | Yunnan | KR782390 | YN2013-A055 | C4a2 |
| 2013 | Yunnan | KR782391 | YN2013-A056 | C4a2 |
| 2013 | Yunnan | KR782392 | YN2013-A057 | C4a2 |
| 2013 | Yunnan | KR782393 | YN2013-A062 | C4a2 |
| 2013 | Yunnan | KR782394 | YN2013-A116 | C4a2 |
| 2013 | Yunnan | KR782395 | YN2013-A128 | C4a2 |
| 2013 | Yunnan | KR782396 | YN2013-A146 | C4a2 |
| 2013 | Yunnan | KR782397 | YN2013-A148 | C4a2 |
| 2013 | Yunnan | KR782398 | YN2013-A149 | C4a2 |
| 2013 | Yunnan | KR782399 | YN2013-A178 | C4a2 |
| 2013 | Yunnan | KR782400 | YN2013-A201 | C4a2 |
| 2013 | Yunnan | KR782401 | YN2013-A202 | C4a2 |
| 2013 | Yunnan | KR782402 | YN2013-A210 | C4a2 |
| 2013 | Yunnan | KR782403 | YN2013-A211 | C4a2 |
| 2013 | Yunnan | KR782404 | YN2013-A212 | C4a2 |
| 2013 | Yunnan | KR782405 | YN2013-A213 | C4a2 |
| 2013 | Yunnan | KR782406 | YN2013-A216 | C4a2 |
| 2013 | Yunnan | KR782407 | YN2013-A217 | C4a2 |
| 2013 | Yunnan | KR782408 | YN2013-C008 | C4a2 |
| 2013 | Yunnan | KR782409 | YN2013-C106 | C4a2 |
| 2013 | Jiangsu | KT327132 | 311/SZ/CHN/2013 | C4a2 |
| 2013 | Jiangsu | KT327134 | 278/SZ/CHN/2013 | C4a2 |
| 2013 | Jiangsu | KT327135 | 279/SZ/CHN/2013 | C4a2 |
| 2013 | Jiangsu | KT327136 | 299/SZ/CHN/2013 | C4a2 |
| 2013 | Jiangsu | KT327141 | 304/SZ/CHN/2013 | C4a2 |
| 2013 | Jiangsu | KT327142 | 281/SZ/CHN/2013 | C4a2 |
| 2013 | Jiangsu | KT327145 | 273/SZ/CHN/2013 | C4a2 |
| 2013 | Jiangsu | KT327146 | 96/SZ/CHN/2013 | C4a2 |
| 2013 | Jiangsu | KT327147 | 91/SZ/CHN/2013 | C4a2 |
| 2013 | Jiangsu | KT717857 | 13MF13_2013.03_JJ | C4a2 |
| 2013 | Jiangsu | KT717858 | 13MG13_2013.03_GG | C4a2 |
| 2013 | Jiangsu | KT717859 | 13MEJ3_2013.04_TZ | C4a2 |
| 2013 | Jiangsu | KT717860 | 13MJ24_2013.05_TX | C4a2 |
| 2013 | Jiangsu | KT717861 | 13MM22_2013.05_JY | C4a2 |
| 2013 | Jiangsu | KT717862 | 13MM25_2013.05_JY | C4a2 |
| 2013 | Jiangsu | KT717863 | 13MA230_2013.05_TZ | C4a2 |
| 2013 | Jiangsu | KT717864 | 13MG21_2013.05_GG | C4a2 |
| 2013 | Jiangsu | KT717865 | 13MM21_2013.05_JY | C4a2 |
| 2013 | Jiangsu | KT717866 | 13MH10_2013.05_TZ | C4a2 |
| 2013 | Jiangsu | KT717867 | 13MA226_2013.05_TZ | C4a2 |
| 2013 | Jiangsu | KT717868 | 13MH13_2013.05_TZ | C4a2 |
| 2013 | Jiangsu | KT717869 | 13MA228_2013.05_TZ | C4a2 |
| 2013 | Jiangsu | KT717870 | 13MA227_2013.05_TZ | C4a2 |
| 2013 | Jiangsu | KT717871 | 13MA225_2013.05_TZ | C4a2 |
| 2013 | Jiangsu | KT717872 | 13MA231_2013.06_TZ | C4a2 |
| 2013 | Jiangsu | KT717873 | 13MA233_2013.07_TZ | C4a2 |
| 2013 | Jiangsu | KT717874 | 13MA236_2013.07_TZ | C4a2 |
| 2013 | Jiangsu | KT717875 | 13MA232_2013.07_TZ | C4a2 |
| 2013 | Jiangsu | KT717876 | 13MF35_2013.07_JJ | C4a2 |
| 2013 | Jiangsu | KT717877 | 13MJ29_2013.06_TX | C4a2 |
| 2013 | Jiangsu | KT717878 | 13MA246_2013.09_TZ | C4a2 |
| 2013 | Jiangsu | KT717879 | 13MG26_2013.06_GG | C4a2 |
| 2013 | Jiangsu | KT717880 | 13MM31_2013.07_JY | C4a2 |
| 2013 | Jiangsu | KT717881 | 13MG28_2013.06_GG | C4a2 |
| 2013 | Jiangsu | KT717882 | 13MD14_2013.06_TZ | C4a2 |
| 2013 | Jiangsu | KT717883 | 13MJ44_2013.09_TX | C4a2 |
| 2013 | Jiangsu | KT717884 | 13MC55_2013.10_XH | C4a2 |
| 2013 | Jiangsu | KT717885 | 13MG46_2013.11_GG | C4a2 |
| 2013 | Jiangsu | KT717886 | 13MA242_2013.08_TZ | C4a2 |
| 2013 | Shandong | KT985001 | 13HF119/QD/2013/EV71 | C4a2 |
| 2013 | Shandong | KT985002 | 13HF121/QD/2013/EV71 | C4a2 |
| 2013 | Shandong | KT985003 | 13HF122/QD/2013/EV71 | C4a2 |
| 2013 | Shandong | KT985004 | 13HF54/QD/2013/EV71 | C4a2 |
| 2013 | Shandong | KT985005 | 13HS117/QD/2013/EV71 | C4a2 |
| 2013 | Shandong | KT985006 | 13HS15/QD/2013/EV71 | C4a2 |
| 2013 | Shandong | KT985007 | 13HS8/QD/2013/EV71 | C4a2 |
| 2013 | Fujian | KU595831 | EV71/FJFZ001/CHN/2013 | C4a2 |
| 2013 | Fujian | KU595832 | EV71/FJFZ004/CHN/2013 | C4a2 |
| 2013 | Fujian | KU595833 | EV71/FJFZ033/CHN/2013 | C4a2 |
| 2013 | Fujian | KU595834 | EV71/FJLY012/CHN/2013 | C4a2 |
| 2013 | Fujian | KU595835 | EV71/FJLY095/CHN/2013 | C4a2 |
| 2013 | Fujian | KU595836 | EV71/FJND011/CHN/2013 | C4a2 |
| 2013 | Fujian | KU595837 | EV71/FJND157/CHN/2013 | C4a2 |
| 2013 | Fujian | KU595838 | EV71/FJNP005/CHN/2013 | C4a2 |
| 2013 | Fujian | KU595839 | EV71/FJNP265/CHN/2013 | C4a2 |
| 2013 | Fujian | KU595840 | EV71/FJNP310/CHN/2013 | C4a2 |
| 2013 | Fujian | KU595841 | EV71/FJNP325/CHN/2013 | C4a2 |
| 2013 | Fujian | KU595842 | EV71/FJNP417/CHN/2013 | C4a2 |
| 2013 | Fujian | KU595843 | EV71/FJPT16G/CHN/2013 | C4a2 |
| 2013 | Fujian | KU595844 | EV71/FJPT052/CHN/2013 | C4a2 |
| 2013 | Fujian | KU595845 | EV71/FJPT068/CHN/2013 | C4a2 |
| 2013 | Fujian | KU595846 | EV71/FJPT086/CHN/2013 | C4a2 |
| 2013 | Fujian | KU595847 | EV71/FJPT91G/CHN/2013 | C4a2 |
| 2013 | Fujian | KU595848 | EV71/FJPT206/CHN/2013 | C4a2 |
| 2013 | Fujian | KU595849 | EV71/FJQZ410/CHN/2013 | C4a2 |
| 2013 | Fujian | KU595850 | EV71/FJSM086/CHN/2013 | C4a2 |
| 2013 | Fujian | KU595851 | EV71/FJSM342/CHN/2013 | C4a2 |
| 2013 | Fujian | KU595852 | EV71/FJXM293/CHN/2013 | C4a2 |
| 2013 | Fujian | KU595853 | EV71/FJZZ315/CHN/2013 | C4a2 |
| 2013 | Fujian | KU595854 | EV71/FJZZ463/CHN/2013 | C4a2 |
| 2013 | Fujian | KU595855 | EV71/FJFZ175/CHN/2013 | C4a2 |
| 2013 | Fujian | KU595856 | EV71/FJLY062/CHN/2013 | C4a2 |
| 2013 | Fujian | KU595857 | EV71/FJND224/CHN/2013 | C4a2 |
| 2013 | Fujian | KU595858 | EV71/FJQZ101/CHN/2013 | C4a2 |
| 2013 | Fujian | KU595859 | EV71/FJQZ133/CHN/2013 | C4a2 |
| 2013 | Fujian | KU595860 | EV71/FJZZ044/CHN/2013 | C4a2 |
| 2013 | Anhui | KX372688 | FL13001T/AH/CHN/2013 | C4a2 |
| 2013 | Anhui | KX372689 | FL13022T/AH/CHN/2013 | C4a2 |
| 2013 | Anhui | KX372690 | FL13047T/AH/CHN/2013 | C4a2 |
| 2013 | Anhui | KX372691 | FL13052T/AH/CHN/2013 | C4a2 |
| 2013 | Anhui | KX372692 | FL13058T/AH/CHN/2013 | C4a2 |
| 2013 | Anhui | KX372693 | FL13061T/AH/CHN/2013 | C4a2 |
| 2013 | Anhui | KX372694 | FL13073T/AH/CHN/2013 | C4a2 |
| 2013 | Anhui | KX372695 | FL13077T/AH/CHN/2013 | C4a2 |
| 2013 | Shanghai | KX871506 | SHEV2013-001 | C4a2 |
| 2013 | Shanghai | KX871507 | SHEV2013-002 | C4a2 |
| 2013 | Shanghai | KX871508 | SHEV2013-003 | C4a2 |
| 2013 | Shanghai | KX871509 | SHEV2013-004 | C4a2 |
| 2013 | Shanghai | KX871510 | SHEV2013-005 | C4a2 |
| 2013 | Shanghai | KX871511 | SHEV2013-006 | C4a2 |
| 2013 | Shanghai | KX871512 | SHEV2013-007 | C4a2 |
| 2013 | Shanghai | KX871514 | SHEV2013-009 | C4a2 |
| 2013 | Shanghai | KX871515 | SHEV2013-010 | C4a2 |
| 2013 | Shanghai | KX871516 | SHEV2013-011 | C4a2 |
| 2013 | Shanghai | KX871517 | SHEV2013-012 | C4a2 |
| 2013 | Shanghai | KX871518 | SHEV2013-013 | C4a2 |
| 2013 | Shanghai | KX871519 | SHEV2013-014 | C4a2 |
| 2013 | Shanghai | KX871520 | SHEV2013-015 | C4a2 |
| 2013 | Shanghai | KX871521 | SHEV2013-028 | C4a2 |
| 2013 | Shanghai | KX871522 | SHEV2013-030 | C4a2 |
| 2013 | Shanghai | KX871523 | SHEV2013-031 | C4a2 |
| 2013 | Shanghai | KX871524 | SHEV2013-102 | C4a2 |
| 2013 | Shanghai | KX871525 | SHEV2013-108 | C4a2 |
| 2013 | Shanghai | KX871526 | SHEV2013-110 | C4a2 |
| 2013 | Shanghai | KX871527 | SHEV2013-111 | C4a2 |
| 2013 | Shanghai | KX871528 | SHEV2013-132 | C4a2 |
| 2013 | Shanghai | KX871529 | SHEV2013-JW1 | C4a2 |
| 2013 | Shanghai | KX871530 | SHEV2013-JW10 | C4a2 |
| 2013 | Shanghai | KX871531 | SHEV2013-JW11 | C4a2 |
| 2013 | Shanghai | KX871532 | SHEV2013-JW14 | C4a2 |
| 2013 | Shanghai | KX871533 | SHEV2013-JW16 | C4a2 |
| 2013 | Shanghai | KX871534 | SHEV2013-JW2 | C4a2 |
| 2013 | Shanghai | KX871535 | SHEV2013-JW20 | C4a2 |
| 2013 | Shanghai | KX871536 | SHEV2013-JW21 | C4a2 |
| 2013 | Shanghai | KX871537 | SHEV2013-JW3 | C4a2 |
| 2013 | Shanghai | KX871538 | SHEV2013-JW4 | C4a2 |
| 2013 | Shanghai | KX871539 | SHEV2013-JW48 | C4a2 |
| 2013 | Shanghai | KX871540 | SHEV2013-JW5 | C4a2 |
| 2013 | Shanghai | KX871541 | SHEV2013-JW6 | C4a2 |
| 2013 | Shanghai | KX871542 | SHEV2013-JW8 | C4a2 |
| 2013 | Shanghai | KX871543 | SHEV2013-JW9 | C4a2 |
| 2013 | Shanghai | KX871544 | SHEV2013-QI1 | C4a2 |
| 2013 | Shanghai | KX871675 | SHEV2013-JW12 | C4a2 |
| 2013 | Shanghai | KX871676 | SHEV2013-JW13 | C4a2 |
| 2013 | Yunnan | KY425527 | CSF15/YN/CHN/2013 | C4a2 |
| 2013 | Yunnan | LC147362 | 50B-YN-CHN-2013 | C4a2 |
| 2013 | Yunnan | LC147363 | 165B-YN-CHN-2013 | C4a2 |
| 2013 | Guangxi | MF185255 | A069_GX_2013 | C4a2 |
| 2013 | Guangxi | MF185256 | A012_GX_2013 | C4a2 |
| 2013 | Guangxi | MF185257 | 195-1_GX_2013 | C4a2 |
| 2013 | Guangxi | MF185258 | A006_GX_2013 | C4a2 |
| 2013 | Guangxi | MF185259 | 184-4_GX_2013 | C4a2 |
| 2013 | Guangxi | MF185260 | 162-1_GX_2013 | C4a2 |
| 2013 | Guangxi | MF185261 | 078-1_GX_2013 | C4a2 |
| 2013 | Guangdong | MF405075 | EV71/SZ08/CHN/2013 | C4a2 |
| 2013 | Guangdong | MF431793 | EV71/SZ17/CHN/2013 | C4a2 |
| 2014 | Henan | KM260035 | 2-Henan-2014 | C4a2 |
| 2014 | Henan | KM260036 | 4-Henan-2014 | C4a2 |
| 2014 | Henan | KM260037 | 5-Henan-2014 | C4a2 |
| 2014 | Henan | KM260038 | 6-Henan-2014 | C4a2 |
| 2014 | Henan | KM260039 | 8-Henan-2014 | C4a2 |
| 2014 | Henan | KM260040 | 9-Henan-2014 | C4a2 |
| 2014 | Henan | KM260041 | 11-Henan-2014 | C4a2 |
| 2014 | Henan | KM260042 | 13-Henan-2014 | C4a2 |
| 2014 | Henan | KM260043 | 14-Henan-2014 | C4a2 |
| 2014 | Henan | KM260044 | 15-Henan-2014 | C4a2 |
| 2014 | Henan | KM260045 | 16-Henan-2014 | C4a2 |
| 2014 | Henan | KM260046 | 17-Henan-2014 | C4a2 |
| 2014 | Henan | KM260047 | 18-Henan-2014 | C4a2 |
| 2014 | Henan | KM260048 | 20-Henan-2014 | C4a2 |
| 2014 | Henan | KM260049 | 21-Henan-2014 | C4a2 |
| 2014 | Henan | KM260051 | 89-Henan-2014 | C4a2 |
| 2014 | Henan | KM260052 | 113-Henan-2014 | C4a2 |
| 2014 | Henan | KM260053 | 118-Henan-2014 | C4a2 |
| 2014 | Henan | KM260054 | 152-Henan-2014 | C4a2 |
| 2014 | Henan | KM260055 | 163-Henan-2014 | C4a2 |
| 2014 | Jiangxi | KP171496 | 34/NC/CHN/2014 | C4a2 |
| 2014 | Jiangxi | KP171497 | 45/NC/CHN/2014 | C4a2 |
| 2014 | Jiangxi | KP171498 | 63/NC/CHN/2014 | C4a2 |
| 2014 | Jiangxi | KP171499 | 67/NC/CHN/2014 | C4a2 |
| 2014 | Jiangxi | KP171500 | 73/NC/CHN/2014 | C4a2 |
| 2014 | Jiangxi | KP171501 | 97/NC/CHN/2014 | C4a2 |
| 2014 | Jiangxi | KP171502 | 156/NC/CHN/2014 | C4a2 |
| 2014 | Jiangxi | KP171503 | 158/NC/CHN/2014 | C4a2 |
| 2014 | Jiangxi | KP171504 | 159/NC/CHN/2014 | C4a2 |
| 2014 | Jiangxi | KP171505 | 163/NC/CHN/2014 | C4a2 |
| 2014 | Jiangxi | KP171506 | 164/NC/CHN/2014 | C4a2 |
| 2014 | Jiangxi | KP171507 | 165/NC/CHN/2014 | C4a2 |
| 2014 | Jiangxi | KP171508 | 166/NC/CHN/2014 | C4a2 |
| 2014 | Jiangxi | KP171509 | 167/NC/CHN/2014 | C4a2 |
| 2014 | Zhejiang | KP723549 | 109/EV71/Wenzhou/CHN/2014 | C4a2 |
| 2014 | Zhejiang | KT008669 | 4/EV71/Wenzhou/CHN/2014 | C4a2 |
| 2014 | Zhejiang | KT008670 | 11/EV71/Wenzhou/CHN/2014 | C4a2 |
| 2014 | Zhejiang | KT008671 | 109/EV71/Wenzhou/CHN/2014 | C4a2 |
| 2014 | Zhejiang | KT008672 | 116/EV71/Wenzhou/CHN/2014 | C4a2 |
| 2014 | Jiangsu | KT327148 | 10/SZ/CHN/2014 | C4a2 |
| 2014 | Jiangsu | KT327149 | 08/SZ/CHN/2014 | C4a2 |
| 2014 | Jiangsu | KT327150 | 14/SZ/CHN/2014 | C4a2 |
| 2014 | Jiangsu | KT327151 | 13/SZ/CHN/2014 | C4a2 |
| 2014 | Jiangsu | KT327152 | 12/SZ/CHN/2014 | C4a2 |
| 2014 | Jiangsu | KT327153 | 09/SZ/CHN/2014 | C4a2 |
| 2014 | Zhejiang | KT345959 | 120/EV71/Wenzhou/CHN/2014 | C4a2 |
| 2014 | Zhejiang | KT345960 | 15/EV71/Wenzhou/CHN/2014 | C4a2 |
| 2014 | Guangdong | KT428644 | EV71/SZ04/CHN/2014 | C4a2 |
| 2014 | Guangdong | KT428645 | EV71/SZ07/CHN/2014 | C4a2 |
| 2014 | Guangdong | KT428646 | EV71/SZ12/CHN/2014 | C4a2 |
| 2014 | Guangdong | KT428647 | EV71/SZ25/CHN/2014 | C4a2 |
| 2014 | Guangdong | KT428648 | EV71/SZ42/CHN/2014 | C4a2 |
| 2014 | Guangdong | KT428649 | EV71/SZ50/CHN/2014 | C4a2 |
| 2014 | Guangdong | KT428650 | EV71/SZ88/CHN/2014 | C4a2 |
| 2014 | Beijing | KU254595 | BJ14-1 | C4a2 |
| 2014 | Beijing | KU254596 | BJ14-2 | C4a2 |
| 2014 | Fujian | KU595862 | EV71/FJFZ282/CHN/2014 | C4a2 |
| 2014 | Fujian | KU595863 | EV71/FJFZ569/CHN/2014 | C4a2 |
| 2014 | Fujian | KU595864 | EV71/FJFZ587/CHN/2014 | C4a2 |
| 2014 | Fujian | KU595865 | EV71/FJFZ365/CHN/2014 | C4a2 |
| 2014 | Fujian | KU595866 | EV71/FJLY180/CHN/2014 | C4a2 |
| 2014 | Fujian | KU595867 | EV71/FJLY130/CHN/2014 | C4a2 |
| 2014 | Fujian | KU595868 | EV71/FJLY306/CHN/2014 | C4a2 |
| 2014 | Fujian | KU595869 | EV71/FJLY310/CHN/2014 | C4a2 |
| 2014 | Fujian | KU595870 | EV71/FJNP342/CHN/2014 | C4a2 |
| 2014 | Fujian | KU595871 | EV71/FJNP398/CHN/2014 | C4a2 |
| 2014 | Fujian | KU595872 | EV71/FJNP108/CHN/2014 | C4a2 |
| 2014 | Fujian | KU595873 | EV71/FJNP214/CHN/2014 | C4a2 |
| 2014 | Fujian | KU595874 | EV71/FJPT035G/CHN/2014 | C4a2 |
| 2014 | Fujian | KU595875 | EV71/FJPT071G/CHN/2014 | C4a2 |
| 2014 | Fujian | KU595876 | EV71/FJPT077G/CHN/2014 | C4a2 |
| 2014 | Fujian | KU595877 | EV71/FJPT096/CHN/2014 | C4a2 |
| 2014 | Fujian | KU595878 | EV71/FJPT102G/CHN/2014 | C4a2 |
| 2014 | Fujian | KU595879 | EV71/FJPT003G/CHN/2014 | C4a2 |
| 2014 | Fujian | KU595880 | EV71/FJPTN036/CHN/2014 | C4a2 |
| 2014 | Fujian | KU595881 | EV71/FJQZ259/CHN/2014 | C4a2 |
| 2014 | Fujian | KU595882 | EV71/FJQZ317/CHN/2014 | C4a2 |
| 2014 | Fujian | KU595883 | EV71/FJQZ338/CHN/2014 | C4a2 |
| 2014 | Fujian | KU595884 | EV71/FJQZ342/CHN/2014 | C4a2 |
| 2014 | Fujian | KU595885 | EV71/FJQZ343/CHN/2014 | C4a2 |
| 2014 | Fujian | KU595886 | EV71/FJSM410/CHN/2014 | C4a2 |
| 2014 | Fujian | KU595887 | EV71/FJSM048/CHN/2014 | C4a2 |
| 2014 | Fujian | KU595888 | EV71/FJXM334/CHN/2014 | C4a2 |
| 2014 | Fujian | KU595889 | EV71/FJXM409/CHN/2014 | C4a2 |
| 2014 | Fujian | KU595890 | EV71/FJXM427/CHN/2014 | C4a2 |
| 2014 | Fujian | KU595891 | EV71/FJZZ476/CHN/2014 | C4a2 |
| 2014 | Fujian | KU595892 | EV71/FJZZ016/CHN/2014 | C4a2 |
| 2014 | Beijing | KU710722 | BJ14-2 | C4a2 |
| 2014 | Beijing | KU710723 | BJ14-6 | C4a2 |
| 2014 | Beijing | KU710724 | BJ14-8 | C4a2 |
| 2014 | Beijing | KU710725 | BJ14-10 | C4a2 |
| 2014 | Beijing | KU710726 | BJ14-12 | C4a2 |
| 2014 | Beijing | KU710727 | BJ14-14 | C4a2 |
| 2014 | Beijing | KU710728 | BJ14-16 | C4a2 |
| 2014 | Beijing | KU710729 | BJ14-18 | C4a2 |
| 2014 | Beijing | KU710730 | BJ14-1 | C4a2 |
| 2014 | Beijing | KU710731 | BJ14-5 | C4a2 |
| 2014 | Beijing | KU710732 | BJ14-7 | C4a2 |
| 2014 | Beijing | KU710733 | BJ14-9 | C4a2 |
| 2014 | Beijing | KU710734 | BJ14-11 | C4a2 |
| 2014 | Beijing | KU710735 | BJ14-13 | C4a2 |
| 2014 | Beijing | KU710736 | BJ14-15 | C4a2 |
| 2014 | Beijing | KU710737 | BJ14-17 | C4a2 |
| 2014 | Beijing | KU710738 | BJ14-19 | C4a2 |
| 2014 | Beijing | KU710739 | BJ14-21 | C4a2 |
| 2014 | Beijing | KU710740 | BJ14-23 | C4a2 |
| 2014 | Henan | KU726099 | 614-2014-Henan | C4a2 |
| 2014 | Henan | KU726100 | 972-2014-Henan | C4a2 |
| 2014 | Henan | KU726101 | 62-2014-Henan | C4a2 |
| 2014 | Henan | KU726102 | 88-2014-Henan | C4a2 |
| 2014 | Henan | KU726103 | 94-2014-Henan | C4a2 |
| 2014 | Henan | KU726104 | 97-2014-Henan | C4a2 |
| 2014 | Henan | KU726105 | 98-2014-Henan | C4a2 |
| 2014 | Henan | KU726106 | 119-2014-Henan | C4a2 |
| 2014 | Henan | KU726107 | 123-2014-Henan | C4a2 |
| 2014 | Henan | KU726108 | 143-2014-Henan | C4a2 |
| 2014 | Henan | KU726109 | 145-2014-Henan | C4a2 |
| 2014 | Henan | KU726110 | 150-2014-Henan | C4a2 |
| 2014 | Henan | KU726111 | 151-2014-Henan | C4a2 |
| 2014 | Henan | KU726112 | 153-2014-Henan | C4a2 |
| 2014 | Henan | KU726113 | 157-2014-Henan | C4a2 |
| 2014 | Henan | KU726114 | 158-2014-Henan | C4a2 |
| 2014 | Henan | KU726115 | 172-2014-Henan | C4a2 |
| 2014 | Henan | KU726116 | 217-2014-Henan | C4a2 |
| 2014 | Henan | KU726117 | 241-2014-Henan | C4a2 |
| 2014 | Henan | KU726118 | 242-2014-Henan | C4a2 |
| 2014 | Henan | KU726119 | 243-2014-Henan | C4a2 |
| 2014 | Henan | KU726120 | 247-2014-Henan | C4a2 |
| 2014 | Henan | KU726121 | 248-2014-Henan | C4a2 |
| 2014 | Henan | KU726122 | 267-2014-Henan | C4a2 |
| 2014 | Henan | KU726123 | 268-2014-Henan | C4a2 |
| 2014 | Henan | KU726124 | 280-2014-Henan | C4a2 |
| 2014 | Henan | KU726125 | 294-2014-Henan | C4a2 |
| 2014 | Henan | KU726126 | 311-2014-Henan | C4a2 |
| 2014 | Henan | KU726127 | 317-2014-Henan | C4a2 |
| 2014 | Henan | KU726128 | 318-2014-Henan | C4a2 |
| 2014 | Henan | KU726129 | 327-2014-Henan | C4a2 |
| 2014 | Henan | KU726130 | 332-2014-Henan | C4a2 |
| 2014 | Henan | KU726131 | 345-2014-Henan | C4a2 |
| 2014 | Henan | KU726132 | 372-2014-Henan | C4a2 |
| 2014 | Henan | KU726133 | 379-2014-Henan | C4a2 |
| 2014 | Henan | KU726134 | 428-2014-Henan | C4a2 |
| 2014 | Henan | KU726135 | 433-2014-Henan | C4a2 |
| 2014 | Henan | KU726136 | 462-2014-Henan | C4a2 |
| 2014 | Henan | KU726137 | 467-2014-Henan | C4a2 |
| 2014 | Henan | KU726138 | 493-2014-Henan | C4a2 |
| 2014 | Henan | KU726139 | 498-2014-Henan | C4a2 |
| 2014 | Henan | KU726140 | 398-2014-Henan | C4a2 |
| 2014 | Henan | KU726141 | 501-2014-Henan | C4a2 |
| 2014 | Henan | KU726142 | 502-2014-Henan | C4a2 |
| 2014 | Henan | KU726143 | 505-2014-Henan | C4a2 |
| 2014 | Henan | KU726144 | 565-2014-Henan | C4a2 |
| 2014 | Henan | KU726145 | 576-2014-Henan | C4a2 |
| 2014 | Henan | KU726146 | 583-2014-Henan | C4a2 |
| 2014 | Henan | KU726147 | 588-2014-Henan | C4a2 |
| 2014 | Henan | KU726148 | 667-2014-Henan | C4a2 |
| 2014 | Henan | KU726149 | 700-2014-Henan | C4a2 |
| 2014 | Henan | KU726150 | 701-2014-Henan | C4a2 |
| 2014 | Henan | KU726151 | 784-2014-Henan | C4a2 |
| 2014 | Henan | KU726152 | 841-2014-Henan | C4a2 |
| 2014 | Henan | KU726153 | 842-2014-Henan | C4a2 |
| 2014 | Henan | KU726154 | 843-2014-Henan | C4a2 |
| 2014 | Henan | KU726155 | 850-2014-Henan | C4a2 |
| 2014 | Henan | KU726156 | 852-2014-Henan | C4a2 |
| 2014 | Henan | KU726157 | 853-2014-Henan | C4a2 |
| 2014 | Henan | KU726158 | 854-2014-Henan | C4a2 |
| 2014 | Henan | KU726159 | 859-2014-Henan | C4a2 |
| 2014 | Henan | KU726160 | 860-2014-Henan | C4a2 |
| 2014 | Henan | KU726161 | 861-2014-Henan | C4a2 |
| 2014 | Henan | KU726162 | 863-2014-Henan | C4a2 |
| 2014 | Henan | KU726163 | 1054-2014-Henan | C4a2 |
| 2014 | Henan | KU726164 | 1055-2014-Henan | C4a2 |
| 2014 | Henan | KU726165 | 1058-2014-Henan | C4a2 |
| 2014 | Henan | KU726166 | 1073-2014-Henan | C4a2 |
| 2014 | Henan | KU726167 | 1074-2014-Henan | C4a2 |
| 2014 | Henan | KU726168 | 1075-2014-Henan | C4a2 |
| 2014 | Henan | KU726169 | 1090-2014-Henan | C4a2 |
| 2014 | Henan | KU726170 | 1097-2014-Henan | C4a2 |
| 2014 | Henan | KU726171 | 1099-2014-Henan | C4a2 |
| 2014 | Henan | KU726172 | 1115-2014-Henan | C4a2 |
| 2014 | Henan | KU726173 | 1116-2014-Henan | C4a2 |
| 2014 | Henan | KU726174 | 1119-2014-Henan | C4a2 |
| 2014 | Henan | KU726175 | 1127-2014-Henan | C4a2 |
| 2014 | Henan | KU726176 | 1142-2014-Henan | C4a2 |
| 2014 | Henan | KU726177 | 1143-2014-Henan | C4a2 |
| 2014 | Henan | KU726178 | 1144-2014-Henan | C4a2 |
| 2014 | Henan | KU726179 | 1150-2014-Henan | C4a2 |
| 2014 | Henan | KU726180 | 405-2014-Henan | C4a2 |
| 2014 | Henan | KU726181 | 407-2014-Henan | C4a2 |
| 2014 | Henan | KU726182 | 408-2014-Henan | C4a2 |
| 2014 | Henan | KU726183 | 413-2014-Henan | C4a2 |
| 2014 | Henan | KU726184 | 414-2014-Henan | C4a2 |
| 2014 | Henan | KU726185 | 538-2014-Henan | C4a2 |
| 2014 | Henan | KU726186 | 545-2014-Henan | C4a2 |
| 2014 | Henan | KU726187 | 561-2014-Henan | C4a2 |
| 2014 | Henan | KU726188 | 563-2014-Henan | C4a2 |
| 2014 | Henan | KU726189 | 566-2014-Henan | C4a2 |
| 2014 | Henan | KU726190 | 568-2014-Henan | C4a2 |
| 2014 | Henan | KU726191 | 590-2014-Henan | C4a2 |
| 2014 | Henan | KU726192 | 877-2014-Henan | C4a2 |
| 2014 | Henan | KU726193 | 878-2014-Henan | C4a2 |
| 2014 | Henan | KU726194 | 970-2014-Henan | C4a2 |
| 2014 | Henan | KU726195 | 983-2014-Henan | C4a2 |
| 2014 | Henan | KU726196 | 985-2014-Henan | C4a2 |
| 2014 | Henan | KU726197 | 987-2014-Henan | C4a2 |
| 2014 | Henan | KU726198 | 990-2014-Henan | C4a2 |
| 2014 | Henan | KU726199 | 992-2014-Henan | C4a2 |
| 2014 | Henan | KU726200 | 977-2014-Henan | C4a2 |
| 2014 | Henan | KU726201 | 1000-2014-Henan | C4a2 |
| 2014 | Henan | KU726202 | 1005-2014-Henan | C4a2 |
| 2014 | Henan | KU726203 | 1016-2014-Henan | C4a2 |
| 2014 | Henan | KU726204 | 1187-2014-Henan | C4a2 |
| 2014 | Henan | KU726205 | 1188-2014-Henan | C4a2 |
| 2014 | Henan | KU726206 | 1214-2014-Henan | C4a2 |
| 2014 | Henan | KU726207 | 1222-2014-Henan | C4a2 |
| 2014 | Henan | KU726208 | 1230-2014-Henan | C4a2 |
| 2014 | Henan | KU726209 | 1231-2014-Henan | C4a2 |
| 2014 | Henan | KU726210 | 1238-2014-Henan | C4a2 |
| 2014 | Henan | KU726211 | 1252-2014-Henan | C4a2 |
| 2014 | Henan | KU726212 | 1272-2014-Henan | C4a2 |
| 2014 | Henan | KU726213 | 1275-2014-Henan | C4a2 |
| 2014 | Henan | KU726214 | 1279-2014-Henan | C4a2 |
| 2014 | Henan | KU726215 | 1324-2014-Henan | C4a2 |
| 2014 | Henan | KU726216 | 1342-2014-Henan | C4a2 |
| 2014 | Henan | KU726217 | 1350-2014-Henan | C4a2 |
| 2014 | Henan | KU726218 | 1351-2014-Henan | C4a2 |
| 2014 | Henan | KU726219 | 1383-2014-Henan | C4a2 |
| 2014 | Henan | KU726220 | 1384-2014-Henan | C4a2 |
| 2014 | Henan | KU726221 | 1385-2014-Henan | C4a2 |
| 2014 | Henan | KU726222 | 1398-2014-Henan | C4a2 |
| 2014 | Henan | KU726223 | 1401-2014-Henan | C4a2 |
| 2014 | Henan | KU726224 | 1404-2014-Henan | C4a2 |
| 2014 | Henan | KU726225 | 1419-2014-Henan | C4a2 |
| 2014 | Henan | KU726226 | 1425-2014-Henan | C4a2 |
| 2014 | Henan | KU726227 | 1441-2014-Henan | C4a2 |
| 2014 | Henan | KU726228 | 1442-2014-Henan | C4a2 |
| 2014 | Henan | KU726229 | 1450-2014-Henan | C4a2 |
| 2014 | Shanghai | KU936120 | SHAPHC5218/SH/CHN/14 | C4a2 |
| 2014 | Shanghai | KU936121 | SHAPHC5251/SH/CHN/14 | C4a2 |
| 2014 | Shanghai | KU936122 | SHAPHC5267/SH/CHN/14 | C4a2 |
| 2014 | Shanghai | KU936123 | SHAPHC5271/SH/CHN/14 | C4a2 |
| 2014 | Shanghai | KU936124 | SHAPHC5287/SH/CHN/14 | C4a2 |
| 2014 | Shanghai | KU936125 | SHAPHC5307/SH/CHN/14 | C4a2 |
| 2014 | Shanghai | KU936126 | SHAPHC5510/SH/CHN/14 | C4a2 |
| 2014 | Shanghai | KU936127 | SHAPHC5589/SH/CHN/14 | C4a2 |
| 2014 | Shanghai | KU936128 | SHAPHC5365/SH/CHN/14 | C4a2 |
| 2014 | Shanghai | KU936129 | SHAPHC5427/SH/CHN/14 | C4a2 |
| 2014 | Shanghai | KU936130 | SHAPHC5330/SH/CHN/14 | C4a2 |
| 2014 | Shanghai | KU936131 | SHAPHC5432/SH/CHN/14 | C4a2 |
| 2014 | Shanghai | KU936132 | SHAPHC5468/SH/CHN/14 | C4a2 |
| 2014 | Shanghai | KX871545 | SHEV2014-016 | C4a2 |
| 2014 | Shanghai | KX871546 | SHEV2014-017 | C4a2 |
| 2014 | Shanghai | KX871547 | SHEV2014-018 | C4a2 |
| 2014 | Shanghai | KX871548 | SHEV2014-019 | C4a2 |
| 2014 | Shanghai | KX871549 | SHEV2014-020 | C4a2 |
| 2014 | Shanghai | KX871550 | SHEV2014-021 | C4a2 |
| 2014 | Shanghai | KX871551 | SHEV2014-022 | C4a2 |
| 2014 | Shanghai | KX871552 | SHEV2014-023 | C4a2 |
| 2014 | Shanghai | KX871553 | SHEV2014-024 | C4a2 |
| 2014 | Shanghai | KX871554 | SHEV2014-025 | C4a2 |
| 2014 | Shanghai | KX871555 | SHEV2014-026 | C4a2 |
| 2014 | Shanghai | KX871556 | SHEV2014-032 | C4a2 |
| 2014 | Shanghai | KX871557 | SHEV2014-033 | C4a2 |
| 2014 | Shanghai | KX871558 | SHEV2014-034 | C4a2 |
| 2014 | Shanghai | KX871559 | SHEV2014-037 | C4a2 |
| 2014 | Shanghai | KX871560 | SHEV2014-038 | C4a2 |
| 2014 | Shanghai | KX871561 | SHEV2014-040 | C4a2 |
| 2014 | Shanghai | KX871562 | SHEV2014-041 | C4a2 |
| 2014 | Shanghai | KX871563 | SHEV2014-042 | C4a2 |
| 2014 | Shanghai | KX871564 | SHEV2014-045 | C4a2 |
| 2014 | Shanghai | KX871565 | SHEV2014-046 | C4a2 |
| 2014 | Shanghai | KX871566 | SHEV2014-047 | C4a2 |
| 2014 | Shanghai | KX871567 | SHEV2014-116 | C4a2 |
| 2014 | Shanghai | KX871568 | SHEV2014-117 | C4a2 |
| 2014 | Shanghai | KX871569 | SHEV2014-120 | C4a2 |
| 2014 | Shanghai | KX871570 | SHEV2014-127 | C4a2 |
| 2014 | Shanghai | KX871571 | SHEV2014-905 | C4a2 |
| 2014 | Shanghai | KX871572 | SHEV2014-906 | C4a2 |
| 2014 | Shanghai | KX871573 | SHEV2014-907 | C4a2 |
| 2014 | Shanghai | KX871574 | SHEV2014-914 | C4a2 |
| 2014 | Shanghai | KX871575 | SHEV2014-918 | C4a2 |
| 2014 | Shanghai | KX871576 | SHEV2014-923 | C4a2 |
| 2014 | Shanghai | KX871577 | SHEV2014-924 | C4a2 |
| 2014 | Shanghai | KX871578 | SHEV2014-925 | C4a2 |
| 2014 | Shanghai | KX871579 | SHEV2014-927 | C4a2 |
| 2014 | Shanghai | KX871580 | SHEV2014-928 | C4a2 |
| 2014 | Shanghai | KX871581 | SHEV2014-931 | C4a2 |
| 2014 | Shanghai | KX871582 | SHEV2014-933 | C4a2 |
| 2014 | Shanghai | KX871583 | SHEV2014-934 | C4a2 |
| 2014 | Shanghai | KX871584 | SHEV2014-939 | C4a2 |
| 2014 | Shanghai | KX871585 | SHEV2014-943 | C4a2 |
| 2014 | Shanghai | KX871586 | SHEV2014-944 | C4a2 |
| 2014 | Shanghai | KX871587 | SHEV2014-945 | C4a2 |
| 2014 | Shanghai | KX871588 | SHEV2014-948 | C4a2 |
| 2014 | Shanghai | KX871589 | SHEV2014-JW1 | C4a2 |
| 2014 | Shanghai | KX871590 | SHEV2014-JW10 | C4a2 |
| 2014 | Shanghai | KX871591 | SHEV2014-JW11 | C4a2 |
| 2014 | Shanghai | KX871592 | SHEV2014-JW14 | C4a2 |
| 2014 | Shanghai | KX871593 | SHEV2014-JW16 | C4a2 |
| 2014 | Shanghai | KX871594 | SHEV2014-JW2 | C4a2 |
| 2014 | Shanghai | KX871595 | SHEV2014-JW21 | C4a2 |
| 2014 | Shanghai | KX871596 | SHEV2014-JW22 | C4a2 |
| 2014 | Shanghai | KX871597 | SHEV2014-JW24 | C4a2 |
| 2014 | Shanghai | KX871598 | SHEV2014-JW25 | C4a2 |
| 2014 | Shanghai | KX871599 | SHEV2014-JW3 | C4a2 |
| 2014 | Shanghai | KX871600 | SHEV2014-JW30 | C4a2 |
| 2014 | Shanghai | KX871601 | SHEV2014-JW31 | C4a2 |
| 2014 | Shanghai | KX871602 | SHEV2014-JW32 | C4a2 |
| 2014 | Shanghai | KX871603 | SHEV2014-JW34 | C4a2 |
| 2014 | Shanghai | KX871604 | SHEV2014-JW35 | C4a2 |
| 2014 | Shanghai | KX871605 | SHEV2014-JW36 | C4a2 |
| 2014 | Shanghai | KX871606 | SHEV2014-JW37 | C4a2 |
| 2014 | Shanghai | KX871607 | SHEV2014-JW4 | C4a2 |
| 2014 | Shanghai | KX871608 | SHEV2014-JW48 | C4a2 |
| 2014 | Shanghai | KX871609 | SHEV2014-JW5 | C4a2 |
| 2014 | Shanghai | KX871610 | SHEV2014-JW6 | C4a2 |
| 2014 | Shanghai | KX871611 | SHEV2014-JW8 | C4a2 |
| 2014 | Shanghai | KX871612 | SHEV2014-JW9 | C4a2 |
| 2014 | Shanghai | KX871677 | SHEV2014-035 | C4a2 |
| 2014 | Shanghai | KX871678 | SHEV2014-044 | C4a2 |
| 2014 | Shanghai | KX871679 | SHEV2014-125 | C4a2 |
| 2014 | Shanghai | KX871680 | SHEV2014-929 | C4a2 |
| 2014 | Shanghai | KX871681 | SHEV2014-932 | C4a2 |
| 2014 | Shanghai | KX871682 | SHEV2014-938 | C4a2 |
| 2014 | Shanghai | KX871683 | SHEV2014-946 | C4a2 |
| 2014 | Shanghai | KX871684 | SHEV2014-947 | C4a2 |
| 2014 | Shanghai | KX871685 | SHEV2014-JW12 | C4a2 |
| 2014 | Shanghai | KX871686 | SHEV2014-JW13 | C4a2 |
| 2014 | Shanghai | KX871687 | SHEV2014-JW20 | C4a2 |
| 2014 | Hubei | KY100879 | EV71-HuB-E47-2014 | C4a2 |
| 2014 | Hubei | KY100880 | EV71-HuB-E01-2014 | C4a2 |
| 2014 | Hubei | KY100881 | EV71-HuB-E02-2014 | C4a2 |
| 2014 | Hubei | KY100882 | EV71-HuB-E22-2014 | C4a2 |
| 2014 | Hubei | KY100883 | EV71-HuB-E03-2014 | C4a2 |
| 2014 | Hubei | KY100884 | EV71-HuB-E14-2014 | C4a2 |
| 2014 | Hubei | KY100885 | EV71-HuB-E05-2014 | C4a2 |
| 2014 | Hubei | KY100886 | EV71-HuB-E11-2014 | C4a2 |
| 2014 | Hubei | KY100887 | EV71-HuB-E21-2014 | C4a2 |
| 2014 | Hubei | KY100888 | EV71-HuB-E13-2014 | C4a2 |
| 2014 | Hubei | KY100889 | EV71-HuB-E09-2014 | C4a2 |
| 2014 | Hubei | KY100890 | EV71-HuB-E41-2014 | C4a2 |
| 2014 | Hubei | KY100891 | EV71-HuB-E18-2014 | C4a2 |
| 2014 | Hubei | KY100892 | EV71-HuB-E23-2014 | C4a2 |
| 2014 | Hubei | KY100893 | EV71-HuB-E24-2014 | C4a2 |
| 2014 | Hubei | KY100894 | EV71-HuB-E26-2014 | C4a2 |
| 2014 | Zhejiang | KY406797 | ZJ/CHN/201401 | C4a2 |
| 2014 | Zhejiang | KY406798 | ZJ/CHN/201402 | C4a2 |
| 2014 | Zhejiang | KY406799 | ZJ/CHN/201403 | C4a2 |
| 2014 | Zhejiang | KY406800 | ZJ/CHN/201404 | C4a2 |
| 2014 | Zhejiang | KY406801 | ZJ/CHN/201405 | C4a2 |
| 2014 | Zhejiang | KY406802 | ZJ/CHN/201406 | C4a2 |
| 2014 | Zhejiang | KY406803 | ZJ/CHN/201407 | C4a2 |
| 2014 | Yunnan | LC120905 | 15-YN-CHN-2014JK | C4a2 |
| 2014 | Yunnan | LC147364 | 40Y-YN-CHN-2014 | C4a2 |
| 2014 | Yunnan | LC147365 | 76B-YN-CHN-2014 | C4a2 |
| 2014 | Yunnan | LC147366 | 98Y-YN-CHN-2014 | C4a2 |
| 2014 | Yunnan | LC147367 | 99Y-YN-CHN-2014 | C4a2 |
| 2014 | Yunnan | LC147368 | 139Y-YN-CHN-2014 | C4a2 |
| 2014 | Yunnan | LC147369 | 140Y-YN-CHN-2014 | C4a2 |
| 2014 | Yunnan | LC147370 | 141Y-YN-CHN-2014 | C4a2 |
| 2014 | Yunnan | LC147371 | 143Y-YN-CHN-2014 | C4a2 |
| 2014 | Yunnan | LC147372 | 147Y-YN-CHN-2014 | C4a2 |
| 2014 | Yunnan | LC411773 | A6/YN/CHN/2014 | C4a2 |
| 2014 | Yunnan | LC411774 | A8/YN/CHN/2014 | C4a2 |
| 2014 | Yunnan | LC411775 | A9/YN/CHN/2014 | C4a2 |
| 2014 | Yunnan | LC411776 | A10/YN/CHN/2014 | C4a2 |
| 2014 | Yunnan | LC411777 | A14/YN/CHN/2014 | C4a2 |
| 2014 | Yunnan | LC411778 | A23/YN/CHN/2014 | C4a2 |
| 2014 | Yunnan | LC411780 | A41/YN/CHN/2014 | C4a2 |
| 2014 | Yunnan | LC411781 | A45/YN/CHN/2014 | C4a2 |
| 2014 | Yunnan | LC411782 | A052/YN/CHN/2014 | C4a2 |
| 2014 | Yunnan | LC411783 | A057/YN/CHN/2014 | C4a2 |
| 2014 | Yunnan | LC411784 | A067/YN/CHN/2014 | C4a2 |
| 2014 | Yunnan | LC411785 | A069/YN/CHN/2014 | C4a2 |
| 2014 | Yunnan | LC411786 | A071/YN/CHN/2014 | C4a2 |
| 2014 | Yunnan | LC411787 | A074/YN/CHN/2014 | C4a2 |
| 2014 | Yunnan | LC411789 | A080/YN/CHN/2014 | C4a2 |
| 2014 | Yunnan | LC411790 | A099/YN/CHN/2014 | C4a2 |
| 2014 | Yunnan | LC411792 | A112/YN/CHN/2014 | C4a2 |
| 2014 | Yunnan | LC411793 | A118/YN/CHN/2014 | C4a2 |
| 2014 | Yunnan | LC411796 | A161/YN/CHN/2014 | C4a2 |
| 2014 | Yunnan | LC411798 | A174/YN/CHN/2014 | C4a2 |
| 2014 | Yunnan | LC411799 | A176/YN/CHN/2014 | C4a2 |
| 2014 | Yunnan | LC411801 | A180/YN/CHN/2014 | C4a2 |
| 2014 | Yunnan | LC411802 | A188/YN/CHN/2014 | C4a2 |
| 2014 | Yunnan | LC411803 | A205/YN/CHN/2014 | C4a2 |
| 2014 | Yunnan | LC411804 | A207/YN/CHN/2014 | C4a2 |
| 2014 | Yunnan | LC411805 | A226/YN/CHN/2014 | C4a2 |
| 2014 | Yunnan | LC411806 | A227/YN/CHN/2014 | C4a2 |
| 2014 | Yunnan | LC411807 | C2/YN/CHN/2014 | C4a2 |
| 2014 | Yunnan | LC411808 | C12/YN/CHN/2014 | C4a2 |
| 2014 | Yunnan | LC411809 | C18/YN/CHN/2014 | C4a2 |
| 2014 | Yunnan | LC411810 | C20/YN/CHN/2014 | C4a2 |
| 2014 | Yunnan | LC411811 | C21/YN/CHN/2014 | C4a2 |
| 2014 | Yunnan | LC411812 | C31/YN/CHN/2014 | C4a2 |
| 2014 | Yunnan | LC411813 | C32/YN/CHN/2014 | C4a2 |
| 2014 | Yunnan | LC411814 | C43/YN/CHN/2014 | C4a2 |
| 2014 | Yunnan | LC411815 | C69/YN/CHN/2014 | C4a2 |
| 2014 | Yunnan | LC411816 | C81/YN/CHN/2014 | C4a2 |
| 2014 | Yunnan | LC411817 | C122/YN/CHN/2014 | C4a2 |
| 2014 | Yunnan | LC411818 | C163/YN/CHN/2014 | C4a2 |
| 2014 | Yunnan | LC411819 | C165/YN/CHN/2014 | C4a2 |
| 2014 | Yunnan | LC411820 | D20/YN/CHN/2014 | C4a2 |
| 2014 | Yunnan | LC411821 | D32/YN/CHN/2014 | C4a2 |
| 2014 | Yunnan | LC411822 | G022/YN/CHN/2014 | C4a2 |
| 2014 | Yunnan | LC411823 | G041/YN/CHN/2014 | C4a2 |
| 2014 | Yunnan | LC411824 | G043/YN/CHN/2014 | C4a2 |
| 2014 | Yunnan | LC411825 | J102/YN/CHN/2014 | C4a2 |
| 2014 | Yunnan | LC411826 | J105/YN/CHN/2014 | C4a2 |
| 2014 | Yunnan | LC411827 | J115/YN/CHN/2014 | C4a2 |
| 2014 | Yunnan | LC411828 | J181/YN/CHN/2014 | C4a2 |
| 2014 | Yunnan | LC411829 | J225/YN/CHN/2014 | C4a2 |
| 2014 | Yunnan | LC411830 | J228/YN/CHN/2014 | C4a2 |
| 2014 | Yunnan | LC411831 | J241/YN/CHN/2014 | C4a2 |
| 2014 | Yunnan | LC411832 | J245/YN/CHN/2014 | C4a2 |
| 2014 | Yunnan | LC411833 | J279/YN/CHN/2014 | C4a2 |
| 2014 | Yunnan | LC411834 | J281/YN/CHN/2014 | C4a2 |
| 2014 | Yunnan | LC411835 | L4/YN/CHN/2014 | C4a2 |
| 2014 | Yunnan | LC411836 | L5/YN/CHN/2014 | C4a2 |
| 2014 | Yunnan | LC411837 | L6/YN/CHN/2014 | C4a2 |
| 2014 | Yunnan | LC411838 | L8/YN/CHN/2014 | C4a2 |
| 2014 | Yunnan | LC411839 | L10/YN/CHN/2014 | C4a2 |
| 2014 | Yunnan | LC411840 | L14/YN/CHN/2014 | C4a2 |
| 2014 | Yunnan | LC411841 | L15/YN/CHN/2014 | C4a2 |
| 2014 | Yunnan | LC411842 | L16/YN/CHN/2014 | C4a2 |
| 2014 | Yunnan | LC411843 | L18/YN/CHN/2014 | C4a2 |
| 2014 | Yunnan | LC411844 | L25/YN/CHN/2014 | C4a2 |
| 2014 | Yunnan | LC411845 | L27/YN/CHN/2014 | C4a2 |
| 2014 | Yunnan | LC411846 | L29/YN/CHN/2014 | C4a2 |
| 2014 | Yunnan | LC411847 | L31/YN/CHN/2014 | C4a2 |
| 2014 | Yunnan | LC411848 | L32/YN/CHN/2014 | C4a2 |
| 2014 | Yunnan | LC411849 | L38/YN/CHN/2014 | C4a2 |
| 2014 | Yunnan | LC411850 | M24/YN/CHN/2014 | C4a2 |
| 2014 | Yunnan | LC411851 | M26/YN/CHN/2014 | C4a2 |
| 2014 | Yunnan | LC411852 | M33/YN/CHN/2014 | C4a2 |
| 2014 | Yunnan | LC411853 | M34/YN/CHN/2014 | C4a2 |
| 2014 | Yunnan | LC411854 | M35/YN/CHN/2014 | C4a2 |
| 2014 | Yunnan | LC411855 | M047/YN/CHN/2014 | C4a2 |
| 2014 | Yunnan | LC411856 | M048/YN/CHN/2014 | C4a2 |
| 2014 | Yunnan | LC411857 | M049/YN/CHN/2014 | C4a2 |
| 2014 | Yunnan | LC411858 | M050/YN/CHN/2014 | C4a2 |
| 2014 | Yunnan | LC411859 | M051/YN/CHN/2014 | C4a2 |
| 2014 | Yunnan | LC411860 | M062/YN/CHN/2014 | C4a2 |
| 2014 | Yunnan | LC411861 | M69/YN/CHN/2014 | C4a2 |
| 2014 | Yunnan | LC411862 | M072/YN/CHN/2014 | C4a2 |
| 2014 | Yunnan | LC411863 | M092/YN/CHN/2014 | C4a2 |
| 2014 | Yunnan | LC411864 | M095/YN/CHN/2014 | C4a2 |
| 2014 | Yunnan | LC411865 | M111/YN/CHN/2014 | C4a2 |
| 2014 | Yunnan | LC411866 | M112/YN/CHN/2014 | C4a2 |
| 2014 | Yunnan | LC411867 | M113/YN/CHN/2014 | C4a2 |
| 2014 | Yunnan | LC411868 | M114/YN/CHN/2014 | C4a2 |
| 2014 | Yunnan | LC411869 | M117/YN/CHN/2014 | C4a2 |
| 2014 | Yunnan | LC411870 | M126/YN/CHN/2014 | C4a2 |
| 2014 | Yunnan | LC411871 | M129/YN/CHN/2014 | C4a2 |
| 2014 | Yunnan | LC411872 | M131/YN/CHN/2014 | C4a2 |
| 2014 | Yunnan | LC411873 | M135/YN/CHN/2014 | C4a2 |
| 2014 | Yunnan | LC411874 | M139/YN/CHN/2014 | C4a2 |
| 2014 | Yunnan | LC411875 | M148/YN/CHN/2014 | C4a2 |
| 2014 | Yunnan | LC411876 | M150/YN/CHN/2014 | C4a2 |
| 2014 | Yunnan | LC411877 | M159/YN/CHN/2014 | C4a2 |
| 2014 | Yunnan | LC411878 | M161/YN/CHN/2014 | C4a2 |
| 2014 | Yunnan | LC411879 | M168/YN/CHN/2014 | C4a2 |
| 2014 | Yunnan | LC411880 | M174/YN/CHN/2014 | C4a2 |
| 2014 | Yunnan | LC411881 | M176/YN/CHN/2014 | C4a2 |
| 2014 | Yunnan | LC411882 | N002/YN/CHN/2014 | C4a2 |
| 2014 | Yunnan | LC411883 | N004/YN/CHN/2014 | C4a2 |
| 2014 | Yunnan | LC411884 | N010/YN/CHN/2014 | C4a2 |
| 2014 | Yunnan | LC411885 | N012/YN/CHN/2014 | C4a2 |
| 2014 | Yunnan | LC411886 | N013/YN/CHN/2014 | C4a2 |
| 2014 | Yunnan | LC411887 | N014/YN/CHN/2014 | C4a2 |
| 2014 | Yunnan | LC411888 | N015/YN/CHN/2014 | C4a2 |
| 2014 | Yunnan | LC411889 | N023/YN/CHN/2014 | C4a2 |
| 2014 | Yunnan | LC411890 | N025/YN/CHN/2014 | C4a2 |
| 2014 | Yunnan | LC411891 | N028/YN/CHN/2014 | C4a2 |
| 2014 | Yunnan | LC411892 | N030/YN/CHN/2014 | C4a2 |
| 2014 | Yunnan | LC411893 | R4/YN/CHN/2014 | C4a2 |
| 2014 | Yunnan | LC411894 | R5/YN/CHN/2014 | C4a2 |
| 2014 | Yunnan | LC411895 | R27/YN/CHN/2014 | C4a2 |
| 2014 | Yunnan | LC411896 | R31/YN/CHN/2014 | C4a2 |
| 2014 | Yunnan | LC411897 | R33/YN/CHN/2014 | C4a2 |
| 2014 | Yunnan | LC411898 | R71/YN/CHN/2014 | C4a2 |
| 2014 | Yunnan | LC411899 | R92/YN/CHN/2014 | C4a2 |
| 2014 | Guangxi | MF185291 | 517_GX_2014 | C4a2 |
| 2014 | Guangxi | MF185292 | 519_GX_2014 | C4a2 |
| 2014 | Guangxi | MF185293 | 521_GX_2014 | C4a2 |
| 2014 | Guangxi | MF185294 | 523_GX_2014 | C4a2 |
| 2014 | Guangxi | MF185295 | 525_GX_2014 | C4a2 |
| 2014 | Guangxi | MF185296 | 527_GX_2014 | C4a2 |
| 2014 | Guangxi | MF185297 | 533_GX_2014 | C4a2 |
| 2014 | Guangxi | MF185298 | 535_GX_2014 | C4a2 |
| 2014 | Guangxi | MF185299 | 537_GX_2014 | C4a2 |
| 2014 | Guangxi | MF185305 | 551_GX_2014 | C4a2 |
| 2014 | Guangxi | MF185306 | 553_GX_2014 | C4a2 |
| 2014 | Guangxi | MF185307 | 555_GX_2014 | C4a2 |
| 2014 | Guangxi | MF185308 | 557_GX_2014 | C4a2 |
| 2014 | Guangxi | MF185309 | 561_GX_2014 | C4a2 |
| 2014 | Guangxi | MF185310 | 565_GX_2014 | C4a2 |
| 2014 | Guangxi | MF185311 | 567_GX_2014 | C4a2 |
| 2014 | Shandong | MG588043 | Zhucheng/355/SD/CHN/2014/EV71 | C4a2 |
| 2014 | Shandong | MG588044 | Zhucheng/354/SD/CHN/2014/EV71 | C4a2 |
| 2014 | Shandong | MG588045 | Xiashan/242/SD/CHN/2014/EV71 | C4a2 |
| 2014 | Shandong | MG588046 | Gaomi/339/SD/CHN/2014/EV71 | C4a2 |
| 2014 | Shandong | MG588047 | Gaomi/337/SD/CHN/2014/EV71 | C4a2 |
| 2014 | Shandong | MG588048 | Gaomi/308/SD/CHN/2014/EV71 | C4a2 |
| 2014 | Shandong | MG588049 | Gaomi/218/SD/CHN/2014/EV71 | C4a2 |
| 2014 | Shandong | MG588050 | Fagnzi/127/SD/CHN/2014/EV71 | C4a2 |
| 2014 | Shandong | MG588051 | Anqiu/91/SD/CHN/2014/EV71 | C4a2 |
| 2014 | Shandong | MG588052 | Anqiu/90/SD/CHN/2014/EV71 | C4a2 |
| 2014 | Anhui | MG773122 | Huainan201401 | C4a2 |
| 2014 | Anhui | MG773123 | Huainan201402 | C4a2 |
| 2014 | Anhui | MG773124 | Huainan201403 | C4a2 |
| 2014 | Anhui | MG773125 | Huainan201404 | C4a2 |
| 2014 | Anhui | MG773126 | Huainan201405 | C4a2 |
| 2014 | Guangdong | MH716145 | EV71/sHFMD01/Shenzhen/2014 | C4a2 |
| 2014 | Guangdong | MH716146 | EV71/sHFMD02/Shenzhen/2014 | C4a2 |
| 2014 | Guangdong | MH716147 | EV71/sHFMD13/Shenzhen/2014 | C4a2 |
| 2014 | Guangdong | MH716148 | EV71/sHFMD24/Shenzhen/2014 | C4a2 |
| 2014 | Chongqing | KU647000 | CQ2014-86 | B5 |
| 2015 | Beijng | KU376383 | 15XCHFM-2 | C4a2 |
| 2015 | Beijng | KU376384 | 15XCHFM-13 | C4a2 |
| 2015 | Beijng | KU376385 | 15XCHFM-35 | C4a2 |
| 2015 | Beijng | KU376386 | 15XCHFM-36 | C4a2 |
| 2015 | Beijng | KU376387 | 15XCHFM-102 | C4a2 |
| 2015 | Beijng | KU376388 | 15XCHFM-155 | C4a2 |
| 2015 | Fujian | KU595893 | EV71/FJQZ230/CHN/2015 | C4a2 |
| 2015 | Fujian | KU595894 | EV71/FJQZ227/CHN/2015 | C4a2 |
| 2015 | Fujian | KU595895 | EV71/FJQZ224/CHN/2015 | C4a2 |
| 2015 | Fujian | KU595896 | EV71/FJQZ190/CHN/2015 | C4a2 |
| 2015 | Fujian | KU595897 | EV71/FJQZ185/CHN/2015 | C4a2 |
| 2015 | Fujian | KU595898 | EV71/FJQZ183/CHN/2015 | C4a2 |
| 2015 | Fujian | KU595899 | EV71/FJQZ105/CHN/2015 | C4a2 |
| 2015 | Fujian | KU595900 | EV71/FJQZ088/CHN/2015 | C4a2 |
| 2015 | Fujian | KU595901 | EV71/FJQZ003/CHN/2015 | C4a2 |
| 2015 | Fujian | KU595902 | EV71/FJPTN027/CHN/2015 | C4a2 |
| 2015 | Fujian | KU595903 | EV71/FJPT063/CHN/2015 | C4a2 |
| 2015 | Fujian | KU595904 | EV71/FJPT040/CHN/2015 | C4a2 |
| 2015 | Fujian | KU595905 | EV71/FJPT029/CHN/2015 | C4a2 |
| 2015 | Fujian | KU595906 | EV71/FJPT020/CHN/2015 | C4a2 |
| 2015 | Fujian | KU595907 | EV71/FJPT008/CHN/2015 | C4a2 |
| 2015 | Fujian | KU595908 | EV71/FJPT005/CHN/2015 | C4a2 |
| 2015 | Fujian | KU595909 | EV71/FJPT003/CHN/2015 | C4a2 |
| 2015 | Fujian | KU595910 | EV71/FJNP168/CHN/2015 | C4a2 |
| 2015 | Fujian | KU595911 | EV71/FJNP151/CHN/2015 | C4a2 |
| 2015 | Fujian | KU595912 | EV71/FJNP065/CHN/2015 | C4a2 |
| 2015 | Fujian | KU595913 | EV71/FJNP007/CHN/2015 | C4a2 |
| 2015 | Fujian | KU595914 | EV71/FJND082/CHN/2015 | C4a2 |
| 2015 | Fujian | KU595915 | EV71/FJND059/CHN/2015 | C4a2 |
| 2015 | Fujian | KU595916 | EV71/FJND028/CHN/2015 | C4a2 |
| 2015 | Fujian | KU595917 | EV71/FJLY070/CHN/2015 | C4a2 |
| 2015 | Fujian | KU595918 | EV71/FJLY069/CHN/2015 | C4a2 |
| 2015 | Fujian | KU595919 | EV71/FJLY064/CHN/2015 | C4a2 |
| 2015 | Fujian | KU595920 | EV71/FJLY055/CHN/2015 | C4a2 |
| 2015 | Fujian | KU595921 | EV71/FJFZ543/CHN/2015 | C4a2 |
| 2015 | Fujian | KU595922 | EV71/FJFZ536/CHN/2015 | C4a2 |
| 2015 | Fujian | KU595923 | EV71/FJFZ524/CHN/2015 | C4a2 |
| 2015 | Fujian | KU595924 | EV71/FJFZ523/CHN/2015 | C4a2 |
| 2015 | Fujian | KU595925 | EV71/FJFZ495/CHN/2015 | C4a2 |
| 2015 | Fujian | KU595926 | EV71/FJFZ494/CHN/2015 | C4a2 |
| 2015 | Fujian | KU595927 | EV71/FJFZ492/CHN/2015 | C4a2 |
| 2015 | Fujian | KU595928 | EV71/FJFZ489/CHN/2015 | C4a2 |
| 2015 | Fujian | KU595929 | EV71/FJFZ471/CHN/2015 | C4a2 |
| 2015 | Fujian | KU595930 | EV71/FJFZ466/CHN/2015 | C4a2 |
| 2015 | Fujian | KU595931 | EV71/FJFZ424/CHN/2015 | C4a2 |
| 2015 | Fujian | KU595932 | EV71/FJFZ422/CHN/2015 | C4a2 |
| 2015 | Fujian | KU595934 | EV71/FJFZ383/CHN/2015 | C4a2 |
| 2015 | Fujian | KU595935 | EV71/FJFZ334/CHN/2015 | C4a2 |
| 2015 | Fujian | KU595936 | EV71/FJFZ333/CHN/2015 | C4a2 |
| 2015 | Fujian | KU595937 | EV71/FJFZ332/CHN/2015 | C4a2 |
| 2015 | Fujian | KU595938 | EV71/FJFZ331/CHN/2015 | C4a2 |
| 2015 | Fujian | KU595939 | EV71/FJFZ308/CHN/2015 | C4a2 |
| 2015 | Fujian | KU595940 | EV71/FJFZ305/CHN/2015 | C4a2 |
| 2015 | Fujian | KU595941 | EV71/FJFZ303/CHN/2015 | C4a2 |
| 2015 | Fujian | KU595942 | EV71/FJFZ294/CHN/2015 | C4a2 |
| 2015 | Fujian | KU595943 | EV71/FJFZ259/CHN/2015 | C4a2 |
| 2015 | Fujian | KU595944 | EV71/FJFZ232/CHN/2015 | C4a2 |
| 2015 | Fujian | KU595945 | EV71/FJFZ228/CHN/2015 | C4a2 |
| 2015 | Fujian | KU595946 | EV71/FJFZ215/CHN/2015 | C4a2 |
| 2015 | Fujian | KU595947 | EV71/FJFZ197/CHN/2015 | C4a2 |
| 2015 | Fujian | KU595948 | EV71/FJFZ042/CHN/2015 | C4a2 |
| 2015 | Fujian | KU595949 | EV71/FJZZ124/CHN/2015 | C4a2 |
| 2015 | Fujian | KU595950 | EV71/FJZZ123/CHN/2015 | C4a2 |
| 2015 | Fujian | KU595951 | EV71/FJZZ078/CHN/2015 | C4a2 |
| 2015 | Fujian | KU595952 | EV71/FJZZ077/CHN/2015 | C4a2 |
| 2015 | Fujian | KU595953 | EV71/FJXM074/CHN/2015 | C4a2 |
| 2015 | Fujian | KU595954 | EV71/FJXM028/CHN/2015 | C4a2 |
| 2015 | Fujian | KU595955 | EV71/FJXM009/CHN/2015 | C4a2 |
| 2015 | Fujian | KU595956 | EV71/FJXM008/CHN/2015 | C4a2 |
| 2015 | Fujian | KU595957 | EV71/FJQZ246/CHN/2015 | C4a2 |
| 2015 | Henan | KU744442 | 1-2015-Henan | C4a2 |
| 2015 | Henan | KU744443 | 2-2015-Henan | C4a2 |
| 2015 | Henan | KU744444 | 13-2015-Henan | C4a2 |
| 2015 | Henan | KU744445 | 20-2015-Henan | C4a2 |
| 2015 | Henan | KU744446 | 25-2015-Henan | C4a2 |
| 2015 | Henan | KU744447 | 53-2015-Henan | C4a2 |
| 2015 | Henan | KU744448 | 54-2015-Henan | C4a2 |
| 2015 | Henan | KU744449 | 59-2015-Henan | C4a2 |
| 2015 | Henan | KU744450 | 67-2015-Henan | C4a2 |
| 2015 | Henan | KU744451 | 68-2015-Henan | C4a2 |
| 2015 | Henan | KU744452 | 69-2015-Henan | C4a2 |
| 2015 | Henan | KU744453 | 78-2015-Henan | C4a2 |
| 2015 | Henan | KU744454 | 79-2015-Henan | C4a2 |
| 2015 | Henan | KU744455 | 83-2015-Henan | C4a2 |
| 2015 | Shandong | KX752783 | Jinan/SD/CHN/2015 | C4a2 |
| 2015 | Shanghai | KX871613 | SHEV2015-015 | C4a2 |
| 2015 | Shanghai | KX871614 | SHEV2015-016 | C4a2 |
| 2015 | Shanghai | KX871615 | SHEV2015-017 | C4a2 |
| 2015 | Shanghai | KX871616 | SHEV2015-018 | C4a2 |
| 2015 | Shanghai | KX871617 | SHEV2015-019 | C4a2 |
| 2015 | Shanghai | KX871618 | SHEV2015-020 | C4a2 |
| 2015 | Shanghai | KX871619 | SHEV2015-023 | C4a2 |
| 2015 | Shanghai | KX871620 | SHEV2015-024 | C4a2 |
| 2015 | Shanghai | KX871621 | SHEV2015-026 | C4a2 |
| 2015 | Shanghai | KX871622 | SHEV2015-027 | C4a2 |
| 2015 | Shanghai | KX871623 | SHEV2015-028 | C4a2 |
| 2015 | Shanghai | KX871624 | SHEV2015-26 | C4a2 |
| 2015 | Shanghai | KX871625 | SHEV2015-933 | C4a2 |
| 2015 | Shanghai | KX871626 | SHEV2015-935 | C4a2 |
| 2015 | Shanghai | KX871627 | SHEV2015-939 | C4a2 |
| 2015 | Shanghai | KX871628 | SHEV2015-940 | C4a2 |
| 2015 | Shanghai | KX871629 | SHEV2015-941 | C4a2 |
| 2015 | Shanghai | KX871630 | SHEV2015-942 | C4a2 |
| 2015 | Shanghai | KX871631 | SHEV2015-943 | C4a2 |
| 2015 | Shanghai | KX871688 | SHEV2015-934 | C4a2 |
| 2015 | Shanghai | KX871689 | SHEV2015-936 | C4a2 |
| 2015 | Yunnan | KX893543 | 32-YN | C4a2 |
| 2015 | Hubei | KY100895 | EV71-HuB-E02-2015 | C4a2 |
| 2015 | Hubei | KY100896 | EV71-HuB-E20-2015 | C4a2 |
| 2015 | Hubei | KY100897 | EV71-HuB-E01-2015 | C4a2 |
| 2015 | Hubei | KY100898 | EV71-HuB-E13-2015 | C4a2 |
| 2015 | Hubei | KY100899 | EV71-HuB-E23-2015 | C4a2 |
| 2015 | Hubei | KY100900 | EV71-HuB-E15-2015 | C4a2 |
| 2015 | Hubei | KY100901 | EV71-HuB-E16-2015 | C4a2 |
| 2015 | Hubei | KY100902 | EV71-HuB-E26-2015 | C4a2 |
| 2015 | Hubei | KY100903 | EV71-HuB-E12-2015 | C4a2 |
| 2015 | Hubei | KY100904 | EV71-HuB-E22-2015 | C4a2 |
| 2015 | Shandong | KY315729 | SD004R/Shandong/China/2015 | C4a2 |
| 2015 | Jiangsu | KY406804 | ZJ/CHN/201501 | C4a2 |
| 2015 | Jiangsu | KY406805 | ZJ/CHN/201502 | C4a2 |
| 2015 | Jiangsu | KY612315 | 2015g03 | C4a2 |
| 2015 | Yunnan | LC147373 | 9Y-YN-CHN-2015 | C4a2 |
| 2015 | Yunnan | LC14737 | 26Y-YN-CHN-2015 | C4a2 |
| 2015 | Yunnan | LC14737 | 27Y-YN-CHN-2015 | C4a2 |
| 2015 | Yunnan | LC14737 | 34Y-YN-CHN-2015 | C4a2 |
| 2015 | Yunnan | LC14737 | 39Y-YN-CHN-2015 | C4a2 |
| 2015 | Yunnan | LC14737 | 48Y-YN-CHN-2015 | C4a2 |
| 2015 | Yunnan | LC412152 | A22-YN-CHN-2015 | C4a2 |
| 2015 | Yunnan | LC412153 | A23-YN-CHN-2015 | C4a2 |
| 2015 | Yunnan | LC412154 | A28-YN-CHN-2015 | C4a2 |
| 2015 | Yunnan | LC412155 | A41-YN-CHN-2015 | C4a2 |
| 2015 | Yunnan | LC412156 | A44-YN-CHN-2015 | C4a2 |
| 2015 | Yunnan | LC412157 | A70-YN-CHN-2015 | C4a2 |
| 2015 | Yunnan | LC412158 | A74-YN-CHN-2015 | C4a2 |
| 2015 | Yunnan | LC412159 | A99-YN-CHN-2015 | C4a2 |
| 2015 | Yunnan | LC412160 | A102-YN-CHN-2015 | C4a2 |
| 2015 | Yunnan | LC412161 | A127-YN-CHN-2015 | C4a2 |
| 2015 | Yunnan | LC412162 | A132-YN-CHN-2015 | C4a2 |
| 2015 | Yunnan | LC412163 | A133-YN-CHN-2015 | C4a2 |
| 2015 | Yunnan | LC412164 | A134-YN-CHN-2015 | C4a2 |
| 2015 | Yunnan | LC412165 | C14-YN-CHN-2015 | C4a2 |
| 2015 | Yunnan | LC412166 | C17-YN-CHN-2015 | C4a2 |
| 2015 | Yunnan | LC412167 | C18-YN-CHN-2015 | C4a2 |
| 2015 | Yunnan | LC412168 | C41-YN-CHN-2015 | C4a2 |
| 2015 | Yunnan | LC412170 | C56-YN-CHN-2015 | C4a2 |
| 2015 | Yunnan | LC412171 | C79-YN-CHN-2015 | C4a2 |
| 2015 | Yunnan | LC412172 | C88-YN-CHN-2015 | C4a2 |
| 2015 | Yunnan | LC412173 | C112-YN-CHN-2015 | C4a2 |
| 2015 | Yunnan | LC412174 | C113-YN-CHN-2015 | C4a2 |
| 2015 | Yunnan | LC412175 | C137-YN-CHN-2015 | C4a2 |
| 2015 | Yunnan | LC412176 | C153-YN-CHN-2015 | C4a2 |
| 2015 | Yunnan | LC412177 | C154-YN-CHN-2015 | C4a2 |
| 2015 | Yunnan | LC412178 | C156-YN-CHN-2015 | C4a2 |
| 2015 | Yunnan | LC412179 | D2-YN-CHN-2015 | C4a2 |
| 2015 | Yunnan | LC412180 | D6-YN-CHN-2015 | C4a2 |
| 2015 | Yunnan | LC412181 | D7-YN-CHN-2015 | C4a2 |
| 2015 | Yunnan | LC412182 | D8-YN-CHN-2015 | C4a2 |
| 2015 | Yunnan | LC412183 | D9-YN-CHN-2015 | C4a2 |
| 2015 | Yunnan | LC412184 | D12-YN-CHN-2015 | C4a2 |
| 2015 | Yunnan | LC412185 | D42-YN-CHN-2015 | C4a2 |
| 2015 | Yunnan | LC412186 | D44-YN-CHN-2015 | C4a2 |
| 2015 | Yunnan | LC412187 | D45-YN-CHN-2015 | C4a2 |
| 2015 | Yunnan | LC412188 | H1-YN-CHN-2015 | C4a2 |
| 2015 | Yunnan | LC412189 | J6-YN-CHN-2015 | C4a2 |
| 2015 | Yunnan | LC412190 | J7-YN-CHN-2015 | C4a2 |
| 2015 | Yunnan | LC412191 | J10-YN-CHN-2015 | C4a2 |
| 2015 | Yunnan | LC412192 | J23-YN-CHN-2015 | C4a2 |
| 2015 | Yunnan | LC412193 | J25-YN-CHN-2015 | C4a2 |
| 2015 | Yunnan | LC412194 | J29-YN-CHN-2015 | C4a2 |
| 2015 | Yunnan | LC412195 | J32-YN-CHN-2015 | C4a2 |
| 2015 | Yunnan | LC412196 | J33-YN-CHN-2015 | C4a2 |
| 2015 | Yunnan | LC412197 | J34-YN-CHN-2015 | C4a2 |
| 2015 | Yunnan | LC412198 | J39-YN-CHN-2015 | C4a2 |
| 2015 | Yunnan | LC412199 | J40-YN-CHN-2015 | C4a2 |
| 2015 | Yunnan | LC412200 | J44-YN-CHN-2015 | C4a2 |
| 2015 | Yunnan | LC412201 | J52-YN-CHN-2015 | C4a2 |
| 2015 | Yunnan | LC412202 | K20-YN-CHN-2015 | C4a2 |
| 2015 | Yunnan | LC412203 | K38-YN-CHN-2015 | C4a2 |
| 2015 | Yunnan | LC412204 | K44-YN-CHN-2015 | C4a2 |
| 2015 | Yunnan | LC412205 | K48-YN-CHN-2015 | C4a2 |
| 2015 | Yunnan | LC412206 | N1-YN-CHN-2015 | C4a2 |
| 2015 | Yunnan | LC412207 | N3-YN-CHN-2015 | C4a2 |
| 2015 | Yunnan | LC412208 | N7-YN-CHN-2015 | C4a2 |
| 2015 | Yunnan | LC412209 | N9-YN-CHN-2015 | C4a2 |
| 2015 | Yunnan | LC412210 | N10-YN-CHN-2015 | C4a2 |
| 2015 | Yunnan | LC412211 | N11-YN-CHN-2015 | C4a2 |
| 2015 | Yunnan | LC412212 | N12-YN-CHN-2015 | C4a2 |
| 2015 | Yunnan | LC412213 | N13-YN-CHN-2015 | C4a2 |
| 2015 | Yunnan | LC412214 | N34-YN-CHN-2015 | C4a2 |
| 2015 | Guangxi | MF185300 | 539_GX_2015 | C4a2 |
| 2015 | Guangxi | MF185301 | 541_GX_2015 | C4a2 |
| 2015 | Guangxi | MF185302 | 543_GX_2015 | C4a2 |
| 2015 | Guangxi | MF185303 | 545_GX_2015 | C4a2 |
| 2015 | Guangxi | MF185304 | 547_GX_2015 | C4a2 |
| 2015 | Beijing | MG214681 | 30-2/2015/BJ | C2 |
| 2015 | Shandong | MG588038 | Shouguang/21/SD/CHN/2015/EV71 | C4a2 |
| 2015 | Shandong | MG588039 | Qingzhou/23/SD/CHN/2015/EV71 | C4a2 |
| 2015 | Shandong | MG588040 | Kuiwen/38/SD/CHN/2015/EV71 | C4a2 |
| 2015 | Shandong | MG58804 | Hanting/53/SD/CHN/2015/EV71 | C4a2 |
| 2015 | Shandong | MG58804 | Hanting/52/SD/CHN/2015/EV71 | C4a2 |
| 2015 | Guangdong | MH716149 | EV71/sHFMD04/Shenzhen/2015 | C4a2 |
| 2015 | Guangdong | MH716150 | EV71/sHFMD12/Shenzhen/2015 | C4a2 |
| 2015 | Guangdong | MH716151 | EV71/sHFMD36/Shenzhen/2015 | C4a2 |
| 2016 | Shanghai | KX871447 | SHCA2016-097 | C4a2 |
| 2016 | Shanghai | KX871451 | SHCA2016-103 | C4a2 |
| 2016 | Shanghai | KX871632 | SHEV2016-023 | C4a2 |
| 2016 | Shanghai | KX871633 | SHEV2016-024 | C4a2 |
| 2016 | Shanghai | KX871634 | SHEV2016-025 | C4a2 |
| 2016 | Shanghai | KX871635 | SHEV2016-026 | C4a2 |
| 2016 | Shanghai | KX871637 | SHEV2016-028 | C4a2 |
| 2016 | Shanghai | KX871638 | SHEV2016-029 | C4a2 |
| 2016 | Shanghai | KX871639 | SHEV2016-030 | C4a2 |
| 2016 | Shanghai | KX871640 | SHEV2016-031 | C4a2 |
| 2016 | Shanghai | KX871641 | SHEV2016-032 | C4a2 |
| 2016 | Shanghai | KX871642 | SHEV2016-033 | C4a2 |
| 2016 | Shanghai | KX871643 | SHEV2016-034 | C4a2 |
| 2016 | Shanghai | KX871644 | SHEV2016-035 | C4a2 |
| 2016 | Shanghai | KX871645 | SHEV2016-036 | C4a2 |
| 2016 | Shanghai | KX871646 | SHEV2016-037 | C4a2 |
| 2016 | Shanghai | KX871647 | SHEV2016-038 | C4a2 |
| 2016 | Shanghai | KX871648 | SHEV2016-039 | C4a2 |
| 2016 | Shanghai | KX871649 | SHEV2016-042 | C4a2 |
| 2016 | Shanghai | KX871650 | SHEV2016-043 | C4a2 |
| 2016 | Shanghai | KX871651 | SHEV2016-044 | C4a2 |
| 2016 | Shanghai | KX871652 | SHEV2016-045 | C4a2 |
| 2016 | Shanghai | KX871653 | SHEV2016-046 | C4a2 |
| 2016 | Shanghai | KX871654 | SHEV2016-047 | C4a2 |
| 2016 | Shanghai | KX871655 | SHEV2016-048 | C4a2 |
| 2016 | Shanghai | KX871656 | SHEV2016-049 | C4a2 |
| 2016 | Shanghai | KX871657 | SHEV2016-050 | C4a2 |
| 2016 | Shanghai | KX871658 | SHEV2016-051 | C4a2 |
| 2016 | Shanghai | KX871659 | SHEV2016-052 | C4a2 |
| 2016 | Shanghai | KX871660 | SHEV2016-053 | C4a2 |
| 2016 | Shanghai | KX871661 | SHEV2016-055 | C4a2 |
| 2016 | Shanghai | KX871662 | SHEV2016-056 | C4a2 |
| 2016 | Shanghai | KX871663 | SHEV2016-057 | C4a2 |
| 2016 | Shanghai | KX871664 | SHEV2016-058 | C4a2 |
| 2016 | Shanghai | KX871665 | SHEV2016-059 | C4a2 |
| 2016 | Shanghai | KX871666 | SHEV2016-060 | C4a2 |
| 2016 | Shanghai | KX871667 | SHEV2016-061 | C4a2 |
| 2016 | Shanghai | KX871668 | SHEV2016-065 | C4a2 |
| 2016 | Shanghai | KX871669 | SHEV2016-066 | C4a2 |
| 2016 | Shanghai | KX871670 | SHEV2016-067 | C4a2 |
| 2016 | Shanghai | KX871671 | SHEV2016-072 | C4a2 |
| 2016 | Shanghai | KX871672 | SHEV2016-073 | C4a2 |
| 2016 | Yunnan | LC412772 | A12-YN-CHN-2016 | C4a2 |
| 2016 | Yunnan | LC412773 | A14-YN-CHN-2016 | C4a2 |
| 2016 | Yunnan | LC412774 | A16-YN-CHN-2016 | C4a2 |
| 2016 | Yunnan | LC412775 | A33-YN-CHN-2016 | C4a2 |
| 2016 | Yunnan | LC412776 | A35-YN-CHN-2016 | C4a2 |
| 2016 | Yunnan | LC412777 | A36-YN-CHN-2016 | C4a2 |
| 2016 | Yunnan | LC412778 | A38-YN-CHN-2016 | C4a2 |
| 2016 | Yunnan | LC412779 | A39-YN-CHN-2016 | C4a2 |
| 2016 | Yunnan | LC412780 | A52-YN-CHN-2016 | C4a2 |
| 2016 | Yunnan | LC412781 | A53-YN-CHN-2016 | C4a2 |
| 2016 | Yunnan | LC412782 | A62-YN-CHN-2016 | C4a2 |
| 2016 | Yunnan | LC412783 | A63-YN-CHN-2016 | C4a2 |
| 2016 | Yunnan | LC412784 | A65-YN-CHN-2016 | C4a2 |
| 2016 | Yunnan | LC412785 | A66-YN-CHN-2016 | C4a2 |
| 2016 | Yunnan | LC412786 | A70-YN-CHN-2016 | C4a2 |
| 2016 | Yunnan | LC412787 | A72-YN-CHN-2016 | C4a2 |
| 2016 | Yunnan | LC412788 | A83-YN-CHN-2016 | C4a2 |
| 2016 | Yunnan | LC412789 | A84-YN-CHN-2016 | C4a2 |
| 2016 | Yunnan | LC412790 | A96-YN-CHN-2016 | C4a2 |
| 2016 | Yunnan | LC412791 | A97-YN-CHN-2016 | C4a2 |
| 2016 | Yunnan | LC412792 | A110-YN-CHN-2016 | C4a2 |
| 2016 | Yunnan | LC412793 | A117-YN-CHN-2016 | C4a2 |
| 2016 | Yunnan | LC412794 | A123-YN-CHN-2016 | C4a2 |
| 2016 | Yunnan | LC412795 | A148-YN-CHN-2016 | C4a2 |
| 2016 | Yunnan | LC412796 | A152-YN-CHN-2016 | C4a2 |
| 2016 | Yunnan | LC412797 | A153-YN-CHN-2016 | C4a2 |
| 2016 | Yunnan | LC412798 | A156-YN-CHN-2016 | C4a2 |
| 2016 | Yunnan | LC412799 | A159-YN-CHN-2016 | C4a2 |
| 2016 | Yunnan | LC412800 | C2-YN-CHN-2016 | C4a2 |
| 2016 | Yunnan | LC412801 | C12-YN-CHN-2016 | C4a2 |
| 2016 | Yunnan | LC412802 | C16-YN-CHN-2016 | C4a2 |
| 2016 | Yunnan | LC412803 | C18-YN-CHN-2016 | C4a2 |
| 2016 | Yunnan | LC412804 | C28-YN-CHN-2016 | C4a2 |
| 2016 | Yunnan | LC412805 | C31-YN-CHN-2016 | C4a2 |
| 2016 | Yunnan | LC412806 | C33-YN-CHN-2016 | C4a2 |
| 2016 | Yunnan | LC412807 | C43-YN-CHN-2016 | C4a2 |
| 2016 | Yunnan | LC412808 | C50-YN-CHN-2016 | C4a2 |
| 2016 | Yunnan | LC412809 | C62-YN-CHN-2016 | C4a2 |
| 2016 | Yunnan | LC412810 | C63-YN-CHN-2016 | C4a2 |
| 2016 | Yunnan | LC412811 | C65-YN-CHN-2016 | C4a2 |
| 2016 | Yunnan | LC412812 | C67-YN-CHN-2016 | C4a2 |
| 2016 | Yunnan | LC412813 | C68-YN-CHN-2016 | C4a2 |
| 2016 | Yunnan | LC412814 | C70-YN-CHN-2016 | C4a2 |
| 2016 | Yunnan | LC412815 | C72-YN-CHN-2016 | C4a2 |
| 2016 | Yunnan | LC412816 | C79-YN-CHN-2016 | C4a2 |
| 2016 | Yunnan | LC412817 | C86-YN-CHN-2016 | C4a2 |
| 2016 | Yunnan | LC412818 | C100-YN-CHN-2016 | C4a2 |
| 2016 | Yunnan | LC412819 | C101-YN-CHN-2016 | C4a2 |
| 2016 | Yunnan | LC412820 | C109-YN-CHN-2016 | C4a2 |
| 2016 | Yunnan | LC412821 | C111-YN-CHN-2016 | C4a2 |
| 2016 | Yunnan | LC412822 | C125-YN-CHN-2016 | C4a2 |
| 2016 | Yunnan | LC412823 | C135-YN-CHN-2016 | C4a2 |
| 2016 | Yunnan | LC412824 | C139-YN-CHN-2016 | C4a2 |
| 2016 | Yunnan | LC412825 | D24-YN-CHN-2016 | C4a2 |
| 2016 | Yunnan | LC412826 | D58-YN-CHN-2016 | C4a2 |
| 2016 | Yunnan | LC412827 | D69-YN-CHN-2016 | C4a2 |
| 2016 | Yunnan | LC412828 | D70-YN-CHN-2016 | C4a2 |
| 2016 | Yunnan | LC412829 | D74-YN-CHN-2016 | C4a2 |
| 2016 | Yunnan | LC412830 | D75-YN-CHN-2016 | C4a2 |
| 2016 | Yunnan | LC412831 | D76-YN-CHN-2016 | C4a2 |
| 2016 | Yunnan | LC412832 | D78-YN-CHN-2016 | C4a2 |
| 2016 | Yunnan | LC412833 | D80-YN-CHN-2016 | C4a2 |
| 2016 | Yunnan | LC412834 | D94-YN-CHN-2016 | C4a2 |
| 2016 | Yunnan | LC412835 | D105-YN-CHN-2016 | C4a2 |
| 2016 | Yunnan | LC412836 | D108-YN-CHN-2016 | C4a2 |
| 2016 | Yunnan | LC412837 | D111-YN-CHN-2016 | C4a2 |
| 2016 | Yunnan | LC412838 | D114-YN-CHN-2016 | C4a2 |
| 2016 | Yunnan | LC412839 | D116-YN-CHN-2016 | C4a2 |
| 2016 | Yunnan | LC412840 | D120-YN-CHN-2016 | C4a2 |
| 2016 | Yunnan | LC412841 | D121-YN-CHN-2016 | C4a2 |
| 2016 | Yunnan | LC412842 | D122-YN-CHN-2016 | C4a2 |
| 2016 | Yunnan | LC412843 | D123-YN-CHN-2016 | C4a2 |
| 2016 | Yunnan | LC412844 | D125-YN-CHN-2016 | C4a2 |
| 2016 | Yunnan | LC412845 | D126-YN-CHN-2016 | C4a2 |
| 2016 | Yunnan | LC412846 | D127-YN-CHN-2016 | C4a2 |
| 2016 | Yunnan | LC412847 | D130-YN-CHN-2016 | C4a2 |
| 2016 | Yunnan | LC412848 | D133-YN-CHN-2016 | C4a2 |
| 2016 | Yunnan | LC412849 | D138-YN-CHN-2016 | C4a2 |
| 2016 | Yunnan | LC412850 | D144-YN-CHN-2016 | C4a2 |
| 2016 | Yunnan | LC412851 | D149-YN-CHN-2016 | C4a2 |
| 2016 | Yunnan | LC412852 | D155-YN-CHN-2016 | C4a2 |
| 2016 | Yunnan | LC412853 | D160-YN-CHN-2016 | C4a2 |
| 2016 | Yunnan | LC412854 | D190-YN-CHN-2016 | C4a2 |
| 2016 | Yunnan | LC412855 | D191-YN-CHN-2016 | C4a2 |
| 2016 | Yunnan | LC412856 | D196-YN-CHN-2016 | C4a2 |
| 2016 | Yunnan | LC412857 | D198-YN-CHN-2016 | C4a2 |
| 2016 | Yunnan | LC412858 | D201-YN-CHN-2016 | C4a2 |
| 2016 | Yunnan | LC412859 | D205-YN-CHN-2016 | C4a2 |
| 2016 | Yunnan | LC412860 | D206-YN-CHN-2016 | C4a2 |
| 2016 | Yunnan | LC412861 | D208-YN-CHN-2016 | C4a2 |
| 2016 | Yunnan | LC412862 | D212-YN-CHN-2016 | C4a2 |
| 2016 | Yunnan | LC412863 | G63-EV-A71-VP1-B5 | B5 |
| 2016 | Yunnan | LC412864 | I9-YN-CHN-2016 | C4a2 |
| 2016 | Yunnan | LC412865 | I10-YN-CHN-2016 | C4a2 |
| 2016 | Yunnan | LC412866 | I13-YN-CHN-2016 | C4a2 |
| 2016 | Yunnan | LC412867 | I22-YN-CHN-2016 | C4a2 |
| 2016 | Yunnan | LC412868 | I37-YN-CHN-2016 | C4a2 |
| 2016 | Yunnan | LC412869 | I48-YN-CHN-2016 | C4a2 |
| 2016 | Yunnan | LC412870 | I54-YN-CHN-2016 | C4a2 |
| 2016 | Yunnan | LC412871 | J9-YN-CHN-2016 | C4a2 |
| 2016 | Yunnan | LC412872 | J35-YN-CHN-2016 | C4a2 |
| 2016 | Yunnan | LC412873 | J37-YN-CHN-2016 | C4a2 |
| 2016 | Yunnan | LC412874 | J56-YN-CHN-2016 | C4a2 |
| 2016 | Yunnan | LC412875 | J64-YN-CHN-2016 | C4a2 |
| 2016 | Yunnan | LC412876 | J92-YN-CHN-2016 | C4a2 |
| 2016 | Yunnan | LC412877 | J107-YN-CHN-2016 | C4a2 |
| 2016 | Yunnan | LC412878 | J129-YN-CHN-2016 | C4a2 |
| 2016 | Yunnan | LC412879 | J138-YN-CHN-2016 | C4a2 |
| 2016 | Yunnan | LC412880 | J139-YN-CHN-2016 | C4a2 |
| 2016 | Yunnan | LC412881 | J147-YN-CHN-2016 | C4a2 |
| 2016 | Yunnan | LC412882 | J154-YN-CHN-2016 | C4a2 |
| 2016 | Yunnan | LC412883 | J155-YN-CHN-2016 | C4a2 |
| 2016 | Yunnan | LC412884 | J174-YN-CHN-2016 | C4a2 |
| 2016 | Yunnan | LC412885 | J176-YN-CHN-2016 | C4a2 |
| 2016 | Yunnan | LC412886 | J177-YN-CHN-2016 | C4a2 |
| 2016 | Yunnan | LC412887 | J178-YN-CHN-2016 | C4a2 |
| 2016 | Yunnan | LC412888 | J180-YN-CHN-2016 | C4a2 |
| 2016 | Yunnan | LC412889 | P31-YN-CHN-2016 | C4a2 |
| 2016 | Yunnan | LC412890 | P45-YN-CHN-2016 | C4a2 |
| 2016 | Yunnan | LC412891 | P71-YN-CHN-2016 | C4a2 |
| 2016 | Yunnan | LC412892 | R15-YN-CHN-2016 | C4a2 |
| 2016 | Yunnan | LC412893 | R36-YN-CHN-2016 | C4a2 |
| 2016 | Yunnan | LC412894 | R40-YN-CHN-2016 | C4a2 |
| 2016 | Yunnan | LC412895 | R45-YN-CHN-2016 | C4a2 |
| 2016 | Yunnan | LC412896 | R53-YN-CHN-2016 | C4a2 |
| 2016 | Yunnan | LC412897 | R56-YN-CHN-2016 | C4a2 |
| 2016 | Yunnan | LC412898 | R79-YN-CHN-2016 | C4a2 |
| 2016 | Yunnan | LC412899 | R97-YN-CHN-2016 | C4a2 |
| 2016 | Yunnan | LC412900 | R98-YN-CHN-2016 | C4a2 |
| 2016 | Yunnan | LC412901 | R101-YN-CHN-2016 | C4a2 |
| 2016 | Shanghai | MF872862 | 2016SHYP001-EV71 | C4a2 |
| 2016 | Shandong | MG588027 | Xiashan/467/SD/CHN/2016/EV71 | C4a2 |
| 2016 | Shandong | MG588028 | Weicheng/501/SD/CHN/2016/EV71 | C4a2 |
| 2016 | Shandong | MG588029 | Shouguang/153/SD/CHN/2016/EV71 | C4a2 |
| 2016 | Shandong | MG588030 | Shouguang/150/SD/CHN/2016/EV71 | C4a2 |
| 2016 | Shandong | MG588031 | Shouguang/144/SD/CHN/2016/EV71 | C4a2 |
| 2016 | Shandong | MG588032 | Hanting/219/SD/CHN/2016/EV71 | C4a2 |
| 2016 | Shandong | MG588033 | Gaomi/553/SD/CHN/2016/EV71 | C4a2 |
| 2016 | Shandong | MG588034 | Anqiu/347/SD/CHN/2016/EV71 | C4a2 |
| 2016 | Shandong | MG588035 | Anqiu/287/SD/CHN/2016/EV71 | C4a2 |
| 2016 | Shandong | MG588036 | Anqiu/286/SD/CHN/2016/EV71 | C4a2 |
| 2016 | Shandong | MG588037 | Anqiu/285/SD/CHN/2016/EV71 | C4a2 |
| 2016 | Zhejiang | MG875331 | 160-50 | C4a2 |
| 2016 | Guangdong | MH716152 | EV71/sHFMD01/Shenzhen/2016 | C4a2 |
| 2016 | Guangdong | MH716153 | EV71/sHFMD02/Shenzhen/2016 | C4a2 |
| 2016 | Guangdong | MH716154 | EV71/sHFMD04/Shenzhen/2016 | C4a2 |
| 2016 | Guangdong | MH716155 | EV71/sHFMD08/Shenzhen/2016 | C4a2 |
| 2017 | Yunnan | LC412988 | A11-YN-CHN-2017 | C4a2 |
| 2017 | Yunnan | LC412989 | A14-YN-CHN-2017 | C4a2 |
| 2017 | Yunnan | LC412990 | A24-YN-CHN-2017 | C4a2 |
| 2017 | Yunnan | LC412991 | A28-YN-CHN-2017 | C4a2 |
| 2017 | Yunnan | LC412992 | A34-YN-CHN-2017 | C4a2 |
| 2017 | Yunnan | LC412993 | A35-YN-CHN-2017 | C4a2 |
| 2017 | Yunnan | LC412994 | A47-YN-CHN-2017 | C4a2 |
| 2017 | Yunnan | LC412995 | A48-YN-CHN-2017 | C4a2 |
| 2017 | Yunnan | LC412996 | A50-YN-CHN-2017 | C4a2 |
| 2017 | Yunnan | LC412997 | A51-YN-CHN-2017 | C4a2 |
| 2017 | Yunnan | LC412998 | A61-YN-CHN-2017 | C4a2 |
| 2017 | Yunnan | LC412999 | A62-YN-CHN-2017 | C4a2 |
| 2017 | Yunnan | LC413000 | A69-YN-CHN-2017 | C4a2 |
| 2017 | Yunnan | LC413001 | A82-YN-CHN-2017 | C4a2 |
| 2017 | Yunnan | LC413002 | A91-YN-CHN-2017 | C4a2 |
| 2017 | Yunnan | LC413003 | A94-YN-CHN-2017 | C4a2 |
| 2017 | Yunnan | LC413004 | A104-YN-CHN-2017 | C4a2 |
| 2017 | Yunnan | LC413005 | A105-YN-CHN-2017 | C4a2 |
| 2017 | Yunnan | LC413006 | A147-YN-CHN-2017 | C4a2 |
| 2017 | Yunnan | LC413007 | A148-YN-CHN-2017 | C4a2 |
| 2017 | Yunnan | LC413008 | A154-YN-CHN-2017 | C4a2 |
| 2017 | Yunnan | LC413009 | B11-YN-CHN-2017 | C4a2 |
| 2017 | Yunnan | LC413010 | B45-YN-CHN-2017 | C4a2 |
| 2017 | Yunnan | LC413011 | B46-YN-CHN-2017 | C4a2 |
| 2017 | Yunnan | LC413012 | B47-YN-CHN-2017 | C4a2 |
| 2017 | Yunnan | LC413013 | B48-YN-CHN-2017 | C4a2 |
| 2017 | Yunnan | LC413014 | B49-YN-CHN-2017 | C4a2 |
| 2017 | Yunnan | LC413015 | B50-YN-CHN-2017 | C4a2 |
| 2017 | Yunnan | LC413016 | B58-YN-CHN-2017 | C4a2 |
| 2017 | Yunnan | LC413017 | B66-YN-CHN-2017 | C4a2 |
| 2017 | Yunnan | LC413018 | B67-YN-CHN-2017 | C4a2 |
| 2017 | Yunnan | LC413019 | B68-YN-CHN-2017 | C4a2 |
| 2017 | Yunnan | LC413020 | B69-YN-CHN-2017 | C4a2 |
| 2017 | Yunnan | LC413021 | B70-YN-CHN-2017 | C4a2 |
| 2017 | Yunnan | LC413022 | B71-YN-CHN-2017 | C4a2 |
| 2017 | Yunnan | LC413023 | B72-YN-CHN-2017 | C4a2 |
| 2017 | Yunnan | LC413024 | B73-YN-CHN-2017 | C4a2 |
| 2017 | Yunnan | LC413025 | B74-YN-CHN-2017 | C4a2 |
| 2017 | Yunnan | LC413026 | B75-YN-CHN-2017 | C4a2 |
| 2017 | Yunnan | LC413027 | B76-YN-CHN-2017 | C4a2 |
| 2017 | Yunnan | LC413028 | C23-YN-CHN-2017 | C4a2 |
| 2017 | Yunnan | LC413029 | C43-YN-CHN-2017 | C4a2 |
| 2017 | Yunnan | LC413030 | C45-YN-CHN-2017 | C4a2 |
| 2017 | Yunnan | LC413031 | C48-YN-CHN-2017 | C4a2 |
| 2017 | Yunnan | LC413032 | C59-YN-CHN-2017 | C4a2 |
| 2017 | Yunnan | LC413033 | C61-YN-CHN-2017 | C4a2 |
| 2017 | Yunnan | LC413034 | C70-YN-CHN-2017 | C4a2 |
| 2017 | Yunnan | LC413035 | C71-YN-CHN-2017 | C4a2 |
| 2017 | Yunnan | LC413036 | C73-YN-CHN-2017 | C4a2 |
| 2017 | Yunnan | LC413037 | D15-YN-CHN-2017 | C4a2 |
| 2017 | Yunnan | LC413038 | D16-YN-CHN-2017 | C4a2 |
| 2017 | Yunnan | LC413039 | D141-YN-CHN-2017 | C4a2 |
| 2017 | Yunnan | LC413040 | D142-YN-CHN-2017 | C4a2 |
| 2017 | Yunnan | LC413041 | H5-YN-CHN-2017 | C4a2 |
| 2017 | Yunnan | LC413042 | H15-YN-CHN-2017 | C4a2 |
| 2017 | Yunnan | LC413043 | H20-YN-CHN-2017 | C4a2 |
| 2017 | Yunnan | LC413044 | H28-YN-CHN-2017 | C4a2 |
| 2017 | Yunnan | LC413045 | H33-YN-CHN-2017 | C4a2 |
| 2017 | Yunnan | LC413046 | I3-YN-CHN-2017 | C4a2 |
| 2017 | Yunnan | LC413047 | I4-YN-CHN-2017 | C4a2 |
| 2017 | Yunnan | LC413048 | I6-YN-CHN-2017 | C4a2 |
| 2017 | Yunnan | LC413049 | I13-YN-CHN-2017 | C4a2 |
| 2017 | Yunnan | LC413050 | I14-YN-CHN-2017 | C4a2 |
| 2017 | Yunnan | LC413051 | I15-YN-CHN-2017 | C4a2 |
| 2017 | Yunnan | LC413052 | I16-YN-CHN-2017 | C4a2 |
| 2017 | Yunnan | LC413053 | I21-YN-CHN-2017 | C4a2 |
| 2017 | Yunnan | LC413054 | J3-YN-CHN-2017 | C4a2 |
| 2017 | Yunnan | LC413055 | J7-YN-CHN-2017 | C4a2 |
| 2017 | Yunnan | LC413056 | J13-YN-CHN-2017 | C4a2 |
| 2017 | Yunnan | LC413057 | J15-YN-CHN-2017 | C4a2 |
| 2017 | Yunnan | LC413058 | J16-YN-CHN-2017 | C4a2 |
| 2017 | Yunnan | LC413059 | J28-YN-CHN-2017 | C4a2 |
| 2017 | Yunnan | LC413060 | J30-YN-CHN-2017 | C4a2 |
| 2017 | Yunnan | LC413061 | J32-YN-CHN-2017 | C4a2 |
| 2017 | Yunnan | LC413062 | J38-YN-CHN-2017 | C4a2 |
| 2017 | Yunnan | LC413063 | J44-YN-CHN-2017 | C4a2 |
| 2017 | Yunnan | LC413064 | J48-YN-CHN-2017 | C4a2 |
| 2017 | Yunnan | LC413065 | J56-YN-CHN-2017 | C4a2 |
| 2017 | Yunnan | LC413066 | J57-YN-CHN-2017 | C4a2 |
| 2017 | Yunnan | LC413067 | J58-YN-CHN-2017 | C4a2 |
| 2017 | Yunnan | LC413068 | J59-YN-CHN-2017 | C4a2 |
| 2017 | Yunnan | LC413069 | J69-YN-CHN-2017 | C4a2 |
| 2017 | Yunnan | LC413070 | J71-YN-CHN-2017 | C4a2 |
| 2017 | Yunnan | LC413071 | J80-YN-CHN-2017 | C4a2 |
| 2017 | Yunnan | LC413072 | J81-YN-CHN-2017 | C4a2 |
| 2017 | Yunnan | LC413073 | J91-YN-CHN-2017 | C4a2 |
| 2017 | Yunnan | LC413074 | N7-YN-CHN-2017 | C4a2 |
| 2017 | Yunnan | LC413075 | P13-YN-CHN-2017 | C4a2 |
| 2017 | Yunnan | LC413076 | P57-YN-CHN-2017 | C4a2 |
| 2017 | Jiangsu | MG431943 | EV71/CZTN01/CHN/2017 | C4a2 |
| 2017 | Jiangsu | MG520666 | EV71-NJ2017iso2 | C4a2 |
| 2017 | Jiangsu | MG581490 | EV71-NJ2017iso1 | C4a2 |
| 2017 | Shandong | MG588018 | Shouguang/204/SD/CHN/2017/EV71 | C4a2 |
| 2017 | Shandong | MG588019 | Hanting/136/SD/CHN/2017/EV71 | C4a2 |
| 2017 | Shandong | MG588020 | Gaoxin/410/SD/CHN/2017/EV71 | C4a2 |
| 2017 | Shandong | MG588021 | Fangzi/434/SD/CHN/2017/EV71 | C4a2 |
| 2017 | Shandong | MG588022 | Fangzi/168/SD/CHN/2017/EV71 | C4a2 |
| 2017 | Shandong | MG588023 | Fangzi/167/SD/CHN/2017/EV71 | C4a2 |
| 2017 | Shandong | MG588024 | Changle/392/SD/CHN/2017/EV71 | C4a2 |
| 2017 | Shandong | MG588025 | Changle/391/SD/CHN/2017/EV71 | C4a2 |
| 2017 | Shandong | MG588026 | Changle/387/SD/CHN/2017/EV71 | C4a2 |
| 2017 | Shandong | MG595264 | Qingzhou/345/SD/CHN/2017/EV71 | C4a2 |
| 2017 | Shandong | MG934553 | Linqu/523/SD/CHN/2017/EV71 | C4a2 |
| 2017 | Shandong | MG934554 | Linqu/524/SD/CHN/2017/EV71 | C4a2 |
| 2017 | Shandong | MG934555 | Qingzhou/703/SD/CHN/2017/EV71 | C4a2 |
| 2017 | Shandong | MG934556 | Qingzhou/704/SD/CHN/2017/EV71 | C4a2 |
| 2017 | Zhejiang | MH167442 | LS201701 | C4a2 |
| 2017 | Zhejiang | MH167443 | LS201702 | C4a2 |
| 2017 | Zhejiang | MH167444 | LS201703 | C4a2 |
| 2017 | Zhejiang | MH167445 | LS201704 | C4a2 |
| 2017 | Zhejiang | MH167446 | LS201705 | C4a2 |
| 2017 | Shandong | MH605008 | VA71/SDJN278/CHN/2017 | C4a2 |
| 2017 | Shandong | MH605009 | EVA71/SDJN268/CHN/2017 | C4a2 |
| 2017 | Shandong | MH605010 | EVA71/SDJN241/CHN/2017 | C4a2 |
| 2017 | Shandong | MH605011 | EVA71/SDJN231/CHN/2017 | C4a2 |
| 2017 | Shandong | MH605012 | EVA71/SDJN230/CHN/2017 | C4a2 |
| 2017 | Shandong | MH605013 | EVA71/SDJN203/CHN/2017 | C4a2 |
| 2017 | Shandong | MH605014 | EVA71/SDJN187/CHN/2017 | C4a2 |
| 2017 | Shandong | MH605015 | EVA71/SDJN090/CHN/2017 | C4a2 |
| 2017 | Shandong | MH605016 | EVA71/SDJN047/CHN/2017 | C4a2 |
| 2017 | Shandong | MH605017 | EVA71/SDJN035/CHN/2017 | C4a2 |
| 2017 | Shandong | MH605018 | EVA71/SDJN409/CHN/2017 | C4a2 |
| 2017 | Shandong | MH605019 | EVA71/SDJN370/CHN/2017 | C4a2 |
| 2017 | Guangdong | MH716156 | EV71/sHFMD01/Shenzhen/2017 | C4a2 |
| 2017 | Guangdong | MH716157 | EV71/sHFMD04/Shenzhen/2017 | C4a2 |
| 2017 | Guangdong | MH716158 | EV71/sHFMD07/Shenzhen/2017 | C4a2 |
| 2017 | Guangdong | MH716159 | EV71/sHFMD08/Shenzhen/2017 | C4a2 |
| 2017 | Guangdong | MH716160 | EV71/sHFMD09/Shenzhen/2017 | C4a2 |
| 2017 | Guangdong | MH716161 | EV71/sHFMD10/Shenzhen/2017 | C4a2 |

Note: EV-A71, enterovirus A71; CVA16, coxasckievirus 16; USA, United States; NA, none available. The EV-A71 strains exhibited in table S1 were used for the phylogenetic analysis based on the nucleotide sequences of the VP1 gene (Fig. 1).

**Supplementary Table S2** The information of EV-A71 strains used in genome sequence comparison and phylogenetic analysis of DL71 strain.

| Accession Numbers | Strain | The length of nucleotide sequence (bp) | | | Genotype |
| --- | --- | --- | --- | --- | --- |
|  |  | Genome | 5’UTR | 3UTR |  |
| U22521 | BrCr-CA-70 | 7408 | 743 | 83 | A |
| AB575912 | 10857 | 7348 | 695 | 71 | B0 |
| AB575911 | 10076 | 7333 | 681 | 70 | B0 |
| AB747373 | Nagoya | 7412 | 747 | 83 | B1 |
| HQ189392 | HUN/1978 | 7412 | 747 | 83 | B1 |
| U22522 | MS/7423/87 | 7411 | 746 | 83 | B2 |
| AB575923 | 20233 | 7284 | 689 | 13 | B2 |
| AM396588 | EV71/SAR/SHA63 | 7402 | 737 | 83 | B3 |
| DQ341367 | MY821-3-SAR-97 | 7411 | 747 | 83 | B3 |
| AJ586873 | EV71/9/97/SHA89 | 7410 | 746 | 82 | B4 |
| AF316321 | 5865/sin/000009 | 7411 | 746 | 83 | B4 |
| DQ341363 | S19841-SAR-03 | 7412 | 747 | 83 | B5 |
| DQ341364 | 5511-SIN-00 | 7412 | 747 | 83 | B5 |
| DQ452074 | 804/NO/03 | 7410 | 745 | 83 | C1 |
| DQ341361 | 1M-AUS-12-00 | 7409 | 744 | 83 | C1 |
| AB575937 | 1416 | 7271 | 656 | 33 | C1 |
| JF738000 | TH-EV71-002 | 7329 | 656 | 91 | C1 |
| KU641501 | 37507/TH/DE 2015 | 7152 | 563 | 7 | C1-like |
| KU641502 | 45849/BE/DE 2015 | 7160 | 563 | 15 | C1-like |
| KU641503 | 45894/BE/DE 2015 | 7156 | 563 | 11 | C1-like |
| KU641504 | 44930/BE/DE 2015 | 7157 | 563 | 12 | C1-like |
| KU641505 | 43538/MV/DE 2015 | 7156 | 563 | 11 | C1-like |
| KU641506 | 44932/BE/DE 2015 | 7156 | 563 | 11 | C1-like |
| KU641507 | 44172/RP/DE 2015 | 7157 | 563 | 12 | C1-like |
| KU641508 | 46411/NW/DE 2015 | 7149 | 563 | 4 | C1-like |
| KY888026 | USA/2016/19522 | 7211 | 554 | 75 | C1-like |
| KX139462 | Jena 15-984 | 7242 | 577 | 83 | C1-like |
| AF304457 | Tainan/5746/98 | 7410 | 745 | 83 | C2 |
| KX372324 | SiICRC02/TH/2013 | 7409 | 744 | 83 | C2 |
| JN992283 | 0964/SYD/98 | 7409 | 744 | 83 | C2 |
| DQ341357 | 7F-AUS-6-99 | 7409 | 744 | 83 | C2 |
| HQ647176 | EV049_07 | 7418 | 741 | 95 | C2 |
| JN835312 | MRS/09/3663 | 7395 | 744 | 69 | C2 |
| KC436270 | V08-2236079 | 7409 | 744 | 83 | C2 |
| AF304458 | Tainan/4643/98 | 7410 | 745 | 83 | C2 |
| AF304459 | Tainan/6092/98 | 7410 | 745 | 83 | C2 |
| HM622391 | 2008-00643 | 7348 | 736 | 30 | C2-like |
| HM622392 | 2008-07776 | 7348 | 736 | 30 | C2-like |
| JQ280307 | 3149 | 7414 | 746 | 86 | C2-like |
| DQ341356 | 03-KOR-00 | 7409 | 744 | 83 | C3 |
| DQ341355 | 06-KOR-00 | 7409 | 744 | 83 | C3 |
| EF063152 | E2005125-TW | 7409 | 744 | 83 | C5 |
| EU527983 | 2007-07364 | 7342 | 725 | 33 | C5 |
| LT719068 | CAF-RS2-NMA-008-03 | 7413 | 748 | 83 | E |
| LT719063 | MAD-3126-11 | 7413 | 748 | 83 | F |
| LT719064 | MAD-7842-10 | 7412 | 747 | 83 | F |
| LT719065 | MAD-72341-04 | 7413 | 748 | 83 | F |
| LT719066 | CAE-CEN-BIY-146-08 | 7413 | 748 | 83 | F |
| AF302996 | SHZH98 | 7408 | 743 | 83 | C4b |
| AY465356 | SHZH03 | 7406 | 742 | 82 | C4a1 |
| EU703812 | EV71/Fuyang.Anhui.P.R.C/17.08/1 | 7405 | 742 | 81 | C4a2 |
| EU703813 | EV71/Fuyang.Anhui.P.R.C/17.08/2 | 7405 | 742 | 81 | C4a2 |
| EU703814 | EV71/Fuyang.Anhui.P.R.C/17.08/3 | 7405 | 742 | 81 | C4a2 |
| EU753365 | 518-03F/SD/CHN/07 | 7405 | 742 | 81 | C4a2 |
| EU753375 | 521-18S/SD/CHN/07 | 7406 | 743 | 81 | C4a2 |
| EU753384 | 522-04T/SD/CHN/07 | 7405 | 742 | 81 | C4a2 |
| EU753397 | 523-05T/SD/CHN/07 | 7406 | 743 | 81 | C4a2 |
| EU753398 | 523-07T/SD/CHN/07 | 7406 | 743 | 81 | C4a2 |
| EU753407 | TC03F/SD/CHN/07 | 7406 | 743 | 81 | C4a2 |
| EU812515 | FY23 | 7409 | 745 | 82 | C4a2 |
| EU864507 | EV71/Zhejiang08 | 7406 | 743 | 81 | C4a2 |
| FJ158600 | DTID/ZJU-62 | 7405 | 742 | 81 | C4a2 |
| FJ158601 | DTID/ZJU-74 | 7405 | 742 | 81 | C4a2 |
| FJ194964 | EV71/GDFS/3/2008 | 7405 | 742 | 81 | C4a2 |
| FJ194965 | EV71/GDSG/17/2008 | 7405 | 742 | 81 | C4a2 |
| FJ360544 | GZ-08-01 | 7404 | 741 | 81 | C4a2 |
| FJ360545 | GZ-08-02 | 7405 | 742 | 81 | C4a2 |
| FJ439769 | Fuyang-0805 | 7405 | 742 | 81 | C4a2 |
| FJ606447 | BJ08-Z004-3 | 7405 | 742 | 81 | C4a2 |
| FJ606448 | BJ08-Z011-4 | 7405 | 742 | 81 | C4a2 |
| FJ606449 | BJ08-Z020-1 | 7405 | 742 | 81 | C4a2 |
| FJ606450 | BJ08-Z025-5 | 7405 | 742 | 81 | C4a2 |
| FJ607334 | 1/SHENZHEN/08/China/HFMD/2008 | 7404 | 741 | 81 | C4a2 |
| FJ607335 | 4/SHENZHEN/08/China/HFMD/2008 | 7404 | 741 | 81 | C4a2 |
| FJ607336 | 28/SHENZHEN/08/China/HFMD/2008 | 7403 | 740 | 81 | C4a2 |
| FJ607337 | 121/SHENZHEN/08/China/HFMD Fatal/2008 | 7404 | 741 | 81 | C4a2 |
| FJ607338 | 605/SHENZHEN/08/China/HFMD Severe/2008 | 7404 | 741 | 81 | C4a2 |
| FJ713137 | Shanghai 036-2009 | 7407 | 744 | 81 | C4a2 |
| GQ892830 | GX/LZ 08-04/08/CHN | 7405 | 742 | 81 | C4a2 |
| GQ994989 | Chongqing1-09-China | 7409 | 743 | 84 | C4a2 |
| GQ994990 | Chongqing2-09-China | 7405 | 742 | 81 | C4a2 |
| GQ994991 | Chongqing3-09-China | 7404 | 742 | 80 | C4a2 |
| GU366191 | Henan10-08-China | 7405 | 742 | 81 | C4a2 |
| GU396280 | EV71/Lanzhou01 | 7405 | 742 | 81 | C4a2 |
| GU434678 | EV71-Hubei-09-China | 7408 | 743 | 83 | A |
| GU459070 | FY23-K12 | 7409 | 745 | 82 | C4a2 |
| GU459071 | FY23-K14 | 7406 | 742 | 82 | C4a2 |
| HM002484 | BJ67 | 7405 | 742 | 81 | C4a2 |
| HM002485 | BJ97 | 7405 | 742 | 81 | C4a2 |
| HM002486 | BJ110 | 7406 | 743 | 81 | C4a2 |
| HM002487 | BJ303 | 7406 | 743 | 81 | C4a2 |
| HM002488 | BJ366 | 7405 | 742 | 81 | C4a2 |
| HM002489 | BJ398 | 7406 | 743 | 81 | C4a2 |
| HM003207 | 87-2008 Xi'an Shaanxi | 7414 | 742 | 90 | C4a2 |
| HM053669 | BJ293 | 7405 | 742 | 81 | C4a2 |
| HM053670 | BJ393 | 7409 | 743 | 84 | C4a2 |
| HM053671 | BJ462 | 7405 | 742 | 81 | C4a2 |
| HM245927 | EV71/Henan/294/2010 | 7409 | 743 | 84 | C4a2 |
| HQ188292 | Fuyang-0805a | 7411 | 745 | 84 | C4b |
| HQ325852 | EV71/HENAN/DC/2010 | 7406 | 743 | 81 | C4a2 |
| HQ400942 | HZ08 | 7405 | 742 | 81 | C4a2 |
| HQ407557 | LN009 | 7405 | 742 | 81 | C4a2 |
| HQ423142 | KMM/09 | 7405 | 742 | 81 | C4a2 |
| HQ423143 | KM186/09 | 7405 | 742 | 81 | C4a2 |
| HQ426649 | 2010FJLY008 | 7403 | 740 | 81 | C4a2 |
| HQ456305 | EV71/Guangzhou/156/2010 | 7405 | 742 | 81 | C4a2 |
| HQ456306 | EV71/Guangzhou/134/2010 | 7405 | 742 | 81 | C4a2 |
| HQ456307 | EV71/Guangzhou/120/2010 | 7405 | 742 | 81 | C4a2 |
| HQ456308 | EV71/Guangzhou/118/2010 | 7405 | 742 | 81 | C4a2 |
| HQ456309 | GZ08-831 | 7405 | 742 | 81 | C4a2 |
| HQ456310 | EV71/Guangzhou/530/2008 | 7405 | 742 | 81 | C4a2 |
| HQ456311 | GZ08-522 | 7405 | 742 | 81 | C4a2 |
| HQ456312 | EV71/Guangzhou/520/2008 | 7405 | 742 | 81 | C4a2 |
| HQ456313 | EV71/Guangzhou/95/2010 | 7405 | 742 | 81 | C4a2 |
| HQ611148 | AH08/06 | 7405 | 742 | 81 | C4a2 |
| HQ825317 | EV71/JN200804 | 7404 | 741 | 81 | C4a2 |
| HQ828086 | NBChina01 | 7414 | 742 | 91 | C4a2 |
| HQ891923 | Shanghai 27-2009 | 7405 | 743 | 80 | C4a2 |
| HQ891924 | Shanghai 28-2009 | 7406 | 743 | 81 | C4a2 |
| HQ891925 | Shanghai 36-2009 | 7407 | 744 | 81 | C4a2 |
| HQ891926 | Shanghai 51-2009 | 7407 | 744 | 81 | C4a2 |
| HQ891927 | Shanghai 64-2009 | 7406 | 743 | 81 | C4a2 |
| HQ891928 | Shanghai 117-2009 | 7405 | 743 | 80 | C4a2 |
| HQ891929 | Shanghai 118-2009 | 7406 | 743 | 81 | C4a2 |
| HQ998852 | EV71/Henan/106/2009 | 7403 | 740 | 81 | C4a2 |
| JF830007 | EV71/Ningbo.CHN/065/2010 | 7406 | 743 | 81 | C4a2 |
| JF913464 | EV71/JN200803 | 7405 | 742 | 81 | C4a2 |
| JN001860 | NB/2010/01 | 7406 | 743 | 81 | C4a2 |
| JN020147 | Luoyang/2011 | 7405 | 742 | 81 | C4a2 |
| JN052925 | Nanyang/2011-China | 7404 | 742 | 80 | C4a2 |
| JN256059 | G288-927F/HeN/CHN/2009 | 7405 | 742 | 81 | C4a2 |
| JN256060 | G333-972F/HeN/CHN/2009 | 7405 | 742 | 81 | C4a2 |
| JN256061 | G398-1037F/HeN/CHN/2009 | 7404 | 741 | 81 | C4a2 |
| JN256062 | M183-1176F/HeN/CHN/2009 | 7406 | 743 | 81 | C4a2 |
| JN256063 | M186-1179F/HeN/CHN/2009 | 7406 | 743 | 81 | C4a2 |
| JN256064 | M188-1181F/HeN/CHN/2009 | 7406 | 743 | 81 | C4a2 |
| JQ086365 | EV71/Ningbo.CHN/107-2/2009 | 7406 | 743 | 81 | C4a2 |
| JQ086366 | EV71/Ningbo.CHN/061/2011 | 7406 | 743 | 81 | C4a2 |
| JQ316638 | HQ09231463 | 7408 | 744 | 81 | C4a2 |
| JQ639383 | HN1360/HN/CHN/2011 | 7414 | 746 | 86 | C4a2 |
| JQ639384 | HN318/HN/CHN/2011 | 7414 | 746 | 86 | C4a2 |
| JQ806378 | 35/Jingdezhen/China/HFMD_Severe/2011 | 7406 | 742 | 82 | C4a2 |
| JX017384 | 01011Y | 7412 | 744 | 86 | C4a2 |
| JX111888 | GD-DG2011-1 | 7405 | 742 | 81 | C4a2 |
| JX111889 | GD-DG2011-2 | 7406 | 743 | 81 | C4a2 |
| JX111890 | GD-DG2011-3 | 7404 | 741 | 81 | C4a2 |
| JX111891 | GD-DG2011-5 | 7406 | 743 | 81 | C4a2 |
| JX111892 | GD-DG2011-8 | 7406 | 743 | 81 | C4a2 |
| JX986737 | Wuhan1042/HuB/CHN/2011 | 7405 | 742 | 81 | C4a2 |
| JX986738 | Wuhan1117/HuB/CHN/2011 | 7405 | 742 | 81 | C4a2 |
| JX986739 | Wuhan1143/HuB/CHN/2011 | 7405 | 742 | 81 | C4a2 |
| KC109780 | 202/Jingdezhen/China/HFMD_Severe/2011 | 7405 | 742 | 81 | C4a2 |
| KC414134 | JiLin-11-China | 7408 | 745 | 81 | C4a2 |
| KC570452 | SH12-036 | 7406 | 743 | 81 | C4a2 |
| KC570453 | SH12-276 | 7405 | 742 | 81 | C4a2 |
| KF142411 | HNCZ/201203 | 7404 | 741 | 81 | C4a2 |
| KF142412 | HNCZ/201208 | 7404 | 741 | 81 | C4a2 |
| KF142413 | HNCZ/201211 | 7404 | 741 | 81 | C4a2 |
| KF501389 | EV71/wuhan/3018/2010 | 7408 | 743 | 83 | A |
| KF826491 | TZ06 | 7405 | 741 | 82 | C4a2 |
| KF982854 | DL71 | 7416 | 746 | 88 | C6 |
| KJ004552 | EV71/Hun11-32/2011 | 7406 | 743 | 81 | C4a2 |
| KJ004553 | EV71/Hun11-4/2011 | 7405 | 742 | 81 | C4a2 |
| KJ004554 | EV71/Hun12-14/2012 | 7406 | 742 | 82 | C4a2 |
| KJ004555 | EV71/Hun12-10/2012 | 7405 | 742 | 81 | C4a2 |
| KJ508182 | Hubei-HG/CHN/2012 | 7405 | 742 | 81 | C4a2 |
| KJ784495 | HuzhouE371/2011/CHN | 7415 | 742 | 91 | C4a2 |
| KJ784496 | HuzhouE696/2012/CHN | 7415 | 742 | 91 | C4a2 |
| KP198623 | Hubei-WH/CHN/2012 | 7407 | 744 | 81 | C4a2 |
| KP198624 | Henan-ZMD/CHN/2012 | 7405 | 742 | 81 | C4a2 |
| KP289417 | EV71/P1027/2013/China | 7412 | 744 | 86 | C4a2 |
| KP289418 | EV71/P1031/2013/China | 7405 | 742 | 81 | C4a2 |
| KP289420 | EV71/P123/2013/China | 7405 | 742 | 81 | C4a2 |
| KP289421 | EV71/P156/2013/China | 7405 | 742 | 81 | C4a2 |
| KP289422 | EV71/P16/2013/China | 7405 | 742 | 81 | C4a2 |
| KP289423 | EV71/P222/2013/China | 7405 | 742 | 81 | C4a2 |
| KP289424 | EV71/P267/2013/China | 7405 | 742 | 81 | C4a2 |
| KP289425 | EV71/P352/2013/China | 7405 | 742 | 81 | C4a2 |
| KP289426 | EV71/P40/2013/China | 7405 | 742 | 81 | C4a2 |
| KP289427 | EV71/P454/2013/China | 7414 | 746 | 86 | C4a2 |
| KP289428 | EV71/P63/2013/China | 7414 | 746 | 86 | C4a2 |
| KP289429 | EV71/P654/2013/China | 7405 | 742 | 81 | C4a2 |
| KP289430 | EV71/P868/2013/China | 7405 | 742 | 81 | C4a2 |
| KP289431 | EV71/P977/2013/China | 7412 | 744 | 86 | C4a2 |
| KP289432 | EV71/P990/2013/China | 7414 | 746 | 86 | C4a2 |
| KT008669 | 4/EV71/Wenzhou/CHN/2014 | 7405 | 742 | 81 | C4a2 |
| KT008670 | 11/EV71/Wenzhou/CHN/2014 | 7405 | 742 | 81 | C4a2 |
| KT008671 | 109/EV71/Wenzhou/CHN/2014 | 7405 | 742 | 81 | C4a2 |
| KT008672 | 116/EV71/Wenzhou/CHN/2014 | 7405 | 742 | 81 | C4a2 |
| KT345959 | 120/EV71/Wenzhou/CHN/2014 | 7405 | 742 | 81 | C4a2 |
| KT345960 | 15/EV71/Wenzhou/CHN/2014 | 7405 | 742 | 81 | C4a2 |
| KT428644 | EV71/SZ04/CHN/2014 | 7405 | 742 | 81 | C4a2 |
| KT428645 | EV71/SZ07/CHN/2014 | 7405 | 742 | 81 | C4a2 |
| KT428646 | EV71/SZ12/CHN/2014 | 7405 | 742 | 81 | C4a2 |
| KT428647 | EV71/SZ25/CHN/2014 | 7405 | 742 | 81 | C4a2 |
| KT428648 | EV71/SZ42/CHN/2014 | 7405 | 742 | 81 | C4a2 |
| KT428649 | EV71/SZ50/CHN/2014 | 7405 | 742 | 81 | C4a2 |
| KT428650 | EV71/SZ88/CHN/2014 | 7405 | 742 | 81 | C4a2 |
| KU254595 | BJ14-1 | 7406 | 742 | 82 | C4a2 |
| KU254596 | BJ14-2 | 7405 | 741 | 82 | C4a2 |
| KU936120 | SHAPHC5218/SH/CHN/14 | 7406 | 743 | 81 | C4a2 |
| KU936121 | SHAPHC5251/SH/CHN/14 | 7406 | 743 | 81 | C4a2 |
| KU936122 | SHAPHC5267/SH/CHN/14 | 7405 | 742 | 81 | C4a2 |
| KU936123 | SHAPHC5271/SH/CHN/14 | 7404 | 741 | 81 | C4a2 |
| KU936124 | SHAPHC5287/SH/CHN/14 | 7406 | 743 | 81 | C4a2 |
| KU936125 | SHAPHC5307/SH/CHN/14 | 7405 | 742 | 81 | C4a2 |
| KU936126 | SHAPHC5510/SH/CHN/14 | 7405 | 742 | 81 | C4a2 |
| KU936127 | SHAPHC5589/SH/CHN/14 | 7405 | 742 | 81 | C4a2 |
| KU936128 | SHAPHC5365/SH/CHN/14 | 7402 | 741 | 79 | C4a2 |
| KU936129 | SHAPHC5427/SH/CHN/14 | 7404 | 743 | 71 | C4a2 |
| KU936130 | SHAPHC5330/SH/CHN/14 | 7405 | 742 | 81 | C4a2 |
| KU936131 | SHAPHC5432/SH/CHN/14 | 7405 | 742 | 81 | C4a2 |
| KU936132 | SHAPHC5468/SH/CHN/14 | 7405 | 742 | 81 | C4a2 |
| KX752783 | Jinan/SD/CHN/2015 | 7406 | 743 | 81 | C4a2 |
| KY315729 | SD004R/Shandong/China/2015 | 7406 | 743 | 81 | C4a2 |
| KY425527 | CSF15/YN/CHN/2013 | 7404 | 741 | 81 | C4a2 |
| KY612315 | 2015g03 | 7404 | 741 | 81 | C4a2 |
| MF405075 | EV71/SZ08/CHN/2013 | 7405 | 742 | 81 | C4a2 |
| MF431793 | EV71/SZ17/CHN/2013 | 7406 | 742 | 82 | C4a2 |
| MG214681 | 30-2/2015/BJ | 7409 | 744 | 83 | C2 |
| MG773123 | Huainan201402 | 7412 | 742 | 88 | C4a2 |
| MG875331 | 160-50 | 7404 | 741 | 81 | C4a2 |

The EV-A71 strains exhibited in Supplementary Table S2 were used for the complete genome sequence comparison (Table 1 and Supplementary Table S3) and the phylogenetic analysis based on the nucleotide sequences of the complete genome and individual genes (Fig. 2).

**Supplementary Table S3** The average genetic distances of the complete genome and individual genes of EV-A71 strains within genotypes or subgenotypes.

| Genotypes | | Genome | 5’UTR | VP4 | VP2 | VP3 | VP1 | 2A | 2B | 2C | 3A | 3B | 3C | 3D | 3’UTR |
| --- | --- | --- | --- | --- | --- | --- | --- | --- | --- | --- | --- | --- | --- | --- | --- |
| A | nt | 0.0078 | 0.0063 | 0.0131 | 0.0026 | 0.0037 | 0.0106 | 0.0060 | 0.0068 | 0.0054 | 0.0376 | 0.0207 | 0.0098 | 0.0072 | 0.0 |
|  | aa | 0.0132 | - | 0.0296 | 0.0079 | 0.0083 | 0.0251 | 0.0134 | 0.0068 | 0.0082 | 0.0 | 0.0325 | 0.0147 | 0.0146 | - |
| B0 | nt | 0.0117 | 0.0074 | 0.0 | 0.0214 | 0.0297 | 0.0045 | 0.0090 | 0.0102 | 0.0051 | 0.0039 | 0.0 | 0.0092 | 0.0029 | 0.294 |
|  | aa | 0.0041 | - | 0.0 | 0.0 | 0.0041 | 0.0136 | 0.0 | 0.0102 | 0.0061 | 0.0 | 0.0 | 0.0 | 0.0022 | - |
| B1 | nt | 0.0277 | 0.0232 | 0.0460 | 0.0228 | 0.0343 | 0.0336 | 0.0369 | 0.0347 | 0.0196 | 0.0237 | 0.0313 | 0.0338 | 0.022 | 0.0372 |
|  | aa | 0.0051 | - | 0.0 | 0.0040 | 0.0 | 0.0034 | 0.0 | 0.0103 | 0.0031 | 0.0235 | 0.0 | 0.0165 | 0.0043 | - |
| B2 | nt | - | - | 0.0506 | 0.0338 | 0.0370 | 0.0252 | 0.0512 | 0.0382 | 0.0562 | 0.0443 | 0.0802 | 0.0397 | 0.038 | - |
|  | aa | 0.0110 | - | 0.0294 | 0.0040 | 0.0041 | 0.0 | 0.0202 | 0.0 | 0.0216 | 0.0235 | 0.1542 | 0.0 | 0.0109 | - |
| B3 | nt | 0.0061 | 0.0151 | 0.0048 | 0.0066 | 0.0041 | 0.0056 | 0.0067 | 0.0068 | 0.0041 | 0.0078 | 0.0154 | 0.0037 | 0.0043 | 0.0 |
|  | aa | 0.0032 | - | 0.0146 | 0.0040 | 0.0 | 0.0068 | 0.0 | 0.0 | 0.0031 | 0.0235 | 0.0 | 0.0 | 0.0 | - |
| B4 | nt | 0.0425 | 0.0317 | 0.0299 | 0.0452 | 0.0401 | 0.0382 | 0.0321 | 0.0420 | 0.0421 | 0.0529 | 0.0646 | 0.0720 | 0.0428 | 0.038 |
|  | aa | 0.0055 | - | 0.0 | 0.0 | 0.0083 | 0.0 | 0.0 | 0.0 | 0.0061 | 0.0355 | 0.0488 | 0.0165 | 0.0022 | - |
| B5 | nt | 0.0250 | 0.0246 | 0.0451 | 0.0242 | 0.0254 | 0.0206 | 0.0112 | 0.0206 | 0.0217 | 0.0238 | 0.0154 | 0.0261 | 0.035 | 0.0 |
|  | aa | 0.0027 | - | 0.0 | 0.0 | 0.0 | 0.0068 | 0.0 | 0.0 | 0.0 | 0.0 | 0.0 | 0.0110 | 0.0043 | - |
| C1 | nt | 0.0327 | 0.0136 | 0.0325 | 0.0362 | 0.0345 | 0.0353 | 0.0371 | 0.0357 | 0.0271 | 0.0402 | 0.0588 | 0.0285 | 0.0324 | 0.0331 |
|  | aa | 0.0058 | - | 0.0073 | 0.0040 | 0.0041 | 0.0034 | 0.0101 | 0.0102 | 0.0015 | 0.0058 | 0.0 | 0.0147 | 0.0065 | - |
| C1-like | nt | 0.0080 | 0.0050 | 0.0057 | 0.0062 | 0.0080 | 0.0077 | 0.0089 | 0.0075 | 0.0109 | 0.0093 | 0.0117 | 0.0078 | 0.0082 | 0.0135 |
|  | aa | 0.0028 | - | 0.0 | 0.0024 | 0.0008 | 0.0027 | 0.0054 | 0.0082 | 0.0012 | 0.0091 | 0.0173 | 0.0 | 0.0029 | - |
| C2 | nt | 0.0477 | 0.0334 | 0.0666 | 0.0462 | 0.0558 | 0.0474 | 0.0659 | 0.0481 | 0.0501 | 0.0617 | 0.0219 | 0.0487 | 0.0541 | 0.0341 |
|  | aa | 0.0086 | - | 0.0052 | 0.0032 | 0.0 | 0.0046 | 0.0072 | 0.0150 | 0.0119 | 0.0157 | 0.0 | 0.0120 | 0.0137 | - |
| C2-like | nt | 0.0030 | 0.0046 | 0.0 | 0.0018 | 0.0037 | 0.0030 | 0.0045 | 0.0000 | 0.0041 | 0.0026 | 0.0 | 0.0 | 0.0039 | 0.0 |
|  | aa | 0.0034 | - | 0.0 | 0.0 | 0.0028 | 0.0045 | 0.0134 | 0.0000 | 0.0041 | 0.0 | 0.0 | 0.0 | 0.0043 | - |
| C3 | nt | 0.0083 | 0.0027 | 0.0 | 0.0066 | 0.0083 | 0.0056 | 0.0112 | 0.0068 | 0.0144 | 0.0157 | 0.0 | 0.0092 | 0.0094 | 0.0 |
|  | aa | 0.0027 | - | 0.0 | 0.0 | 0.0 | 0.0 | 0.0202 | 0.0 | 0.0061 | 0.0 | 0.0 | 0.0055 | 0.0 | - |
| C4 | nt | 0.0392 | 0.0269 | 0.0340 | 0.0395 | 0.0421 | 0.0356 | 0.0442 | 0.0467 | 0.0369 | 0.0469 | 0.0516 | 0.0377 | 0.0456 | 0.0394 |
|  | aa | 0.0100 | - | 0.0058 | 0.0069 | 0.0040 | 0.0095 | 0.0147 | 0.0081 | 0.0069 | 0.0168 | 0.0266 | 0.0118 | 0.0141 | - |
| C5 | nt | - | - | 0.0197 | 0.0228 | 0.0225 | 0.0275 | 0.0203 | 0.0382 | 0.0324 | 0.0118 | 0.0154 | 0.0457 | 0.0243 | - |
|  | aa | 0.0050 | - | 0.0 | 0.0 | 0.0 | 0.0102 | 0.0134 | 0.0204 | 0.0 | 0.0117 | 0.0 | 0.0055 | 0.0043 | - |
| C6 | nt | n/c | n/c | n/c | n/c | n/c | n/c | n/c | n/c | n/c | n/c | n/c | n/c | n/c | n/c |
|  | aa | n/c | n/c | n/c | n/c | n/c | n/c | n/c | n/c | n/c | n/c | n/c | n/c | n/c | n/c |
| E | nt | n/c | n/c | n/c | n/c | n/c | n/c | n/c | n/c | n/c | n/c | n/c | n/c | n/c | n/c |
|  | aa | n/c | n/c | n/c | n/c | n/c | n/c | n/c | n/c | n/c | n/c | n/c | n/c | n/c | n/c |
| F | nt | 0.0849 | 0.0454 | 0.0959 | 0.0822 | 0.0572 | 0.0655 | 0.0905 | 0.0665 | 0.1078 | 0.1133 | 0.0891 | 0.1067 | 0.1094 | 0.0731 |
|  | aa | 0.0175 | - | 0.0246 | 0.0086 | 0.0048 | 0.0199 | 0.0237 | 0.0170 | 0.0175 | 0.0542 | 0.0244 | 0.0157 | 0.0182 | - |

Note: The information of EV-A71 strains were listed in Supplementary Table S2. The presence of “-” and “n/c” in the results denotes cases in which it was not possible to estimate evolutionary distances. Because that the genome, 5’UTR and 3’UTR sequences of two reference strains belonging to subgenotype B2 were significant different in length (Supplementary Table S2), the genetic distances of these sequences within B2 were not calculated. Because that the genome, 5’UTR and 3’UTR sequences of two reference strains belonging to subgenotype C5 were significant different in length (Supplementary Table S2), the genetic distances of these sequences within C5 were not calculated. Since genotype E and subgenotype C6 both contained only one EV-A71 strain with the complete genome, the genetic distances within these lineages could not be calculated.

**Supplementary Table S4** The complete genome sequences of DL71 and 42 reference strains used in recombination analysis.

| Strains | Isolates/Genotype | Strains | Isolates/Genotype |
| --- | --- | --- | --- |
| AB575911 | 10076/B0 | KF982854 | DL71/C6 |
| AB575912 | 10857/B0 | KX372324 | SiICRC02/TH/2013/C2 |
| AB747373 | Nagoya/B1 | LT719063 | MAD-3126-11/F |
| AF302996 | SHZH98/C4 | LT719064 | MAD-7842-10/F |
| AF304457 | Tainan/5746/98/C2 | LT719065 | MAD-72341-04/F |
| AF316321 | 5865/sin/000009/B4 | LT719066 | CAE-CEN-BIY-146-08/F |
| AJ586873 | EV71/9/97/SHA89/B4 | LT719068 | CAF-RS2-NMA-008-03/E |
| AM396588 | EV71/SAR/SHA63/B3 | MG214681 | 30-2/2015/BJ/C2 |
| AY465356 | SHZH03/C4 | U22521 | BrCr/A |
| DQ341355 | 06-KOR-00/C3 | U22522 | MS/7423/87/B2 |
| DQ341356 | 03-KOR-00/C3 | CVA2 | CVA2 Fleetwood |
| DQ341361 | 1M-AUS-12-00/C1 | CVA3 | CVA3 Olson |
| DQ341363 | S19841-SAR-03/B5 | CVA4 | CVA4 High Point |
| DQ341364 | 5511-SIN-00/B5 | CVA5 | CVA5 Swartz |
| DQ341367 | MY821-3-SAR-97/B3 | CVA6 | CVA6 Gdula |
| DQ452074 | 804/NO/03/C1 | CVA7 | CVA7 Parker |
| EF063152 | E2005125-TW/C5 | CVA8 | CVA8 Donovan |
| GU434678 | EV71-Hubei-09-China/A | CVA10 | CVA10 Kowalik |
| HQ188292 | Fuyang-0805a/C4 | CVA12 | CVA12 Texas-12 |
| HQ189392 | HUN/1978/B1 | CVA14 | CVA14 G-14 |
| JQ316638 | HQ09231463/C4 | CVA16 | CVA16 G-10 |
| KF501389 | EV71/wuhan/3018/2010/A |  |  |

**Supplementary Table S5** Fragments in genome of EV-A71 strains corresponding to the putative recombination events identified in DL71.

| Strains | Isolates/Genotype | event-1 | event-2 | event-3 | event-4 | event-5 | event-6 | event-7 | event-8 | event-9 |
| --- | --- | --- | --- | --- | --- | --- | --- | --- | --- | --- |
| AB575911 | 10076/B0 | 235-527 | 747-1049 | 2229-2596 | 2841-3188 | 3536-3888 | 4216-4517 | 5663-6035 | 6403-6763 | 7065-7307 |
| AB747373 | Nagoya/B1 | 300-593 | 813-1115 | 2295-2662 | 2907-3254 | 3602-3954 | 4282-4583 | 5729-6101 | 6469-6829 | 7131-7375 |
| AF302996 | SHZH98/C4 | 298-591 | 809-1111 | 2291-2658 | 2903-3250 | 3598-3950 | 4278-4579 | 5725-6097 | 6465-6825 | 7127-7371 |
| AF304457 | Tainan/5746/98/C2 | 298-591 | 811-1113 | 2293-2660 | 2905-3252 | 3600-3952 | 4280-4581 | 5727-6099 | 6467-6827 | 7129-7373 |
| AJ586873 | EV71/9/97/SHA89/B4 | 299-592 | 812-1114 | 2294-2661 | 2906-3253 | 3601-3953 | 4281-4582 | 5728-6100 | 6468-6828 | 7130-7374 |
| AM396588 | EV71/SAR/SHA63/B3 | 290-583 | 803-1105 | 2285-2652 | 2897-3244 | 3592-3944 | 4272-4573 | 5719-6091 | 6459-6819 | 7121-7365 |
| AY465356 | SHZH03/C4 | 295-588 | 808-1110 | 2290-2657 | 2902-3249 | 3597-3949 | 4277-4578 | 5724-6096 | 6464-6824 | 7126-7368 |
| DQ341355 | 06-KOR-00/C3 | 297-590 | 810-1112 | 2292-2659 | 2904-3251 | 3599-3951 | 4279-4580 | 5726-6098 | 6466-6826 | 7128-7372 |
| DQ341363 | S19841-SAR-03/B5 | 300-593 | 813-1115 | 2295-2662 | 2907-3254 | 3602-3954 | 4282-4583 | 5729-6101 | 6469-6829 | 7131-7375 |
| DQ452074 | 804/NO/03/C1 | 298-591 | 811-1113 | 2293-2660 | 2905-3252 | 3600-3952 | 4280-4581 | 5727-6099 | 6467-6827 | 7129-7373 |
| EF063152 | E2005125-TW/C5 | 297-590 | 810-1112 | 2292-2659 | 2904-3251 | 3599-3951 | 4279-4580 | 5726-6098 | 6466-6826 | 7128-7372 |
| HQ188292 | Fuyang-0805a/C4 | 298-591 | 811-1113 | 2293-2660 | 2905-3252 | 3600-3952 | 4280-4581 | 5727-6099 | 6467-6827 | 7129-7371 |
| JQ316638 | HQ09231463/C4 | 297-590 | 810-1112 | 2292-2659 | 2904-3251 | 3599-3951 | 4279-4580 | 5726-6098 | 6466-6826 | 7128-7370 |
| KF982854 | DL71/C6 | 299-592 | 812-1114 | 2294-2661 | 2906-3253 | 3601-3953 | 4281-4582 | 5728-6100 | 6468-6828 | 7130-7374 |
| KX372324 | SiICRC02/TH/2013/C2 | 297-590 | 810-1112 | 2292-2659 | 2904-3251 | 3599-3951 | 4279-4580 | 5726-6098 | 6466-6826 | 7128-7372 |
| LT719065 | MAD-72341-04/F | 301-594 | 814-1116 | 2296-2663 | 2908-3255 | 3603-3955 | 4283-4584 | 5730-6102 | 6470-6830 | 7132-7376 |
| LT719068 | CAF-RS2-NMA-008-03/E | 301-594 | 814-1116 | 2296-2663 | 2908-3255 | 3603-3955 | 4283-4584 | 5730-6102 | 6470-6830 | 7132-7376 |
| MG214681 | 30-2/2015/BJ/C2 | 297-590 | 810-1112 | 2292-2659 | 2904-3251 | 3599-3951 | 4279-4580 | 5726-6098 | 6466-6826 | 7128-7372 |
| U22521 | BrCr/A | 298-591 | 809-1111 | 2291-2658 | 2903-3250 | 3598-3950 | 4278-4579 | 5725-6097 | 6465-6825 | 7127-7371 |
| U22522 | MS/7423/87/B2 | 299-592 | 812-1114 | 2294-2661 | 2906-3253 | 3601-3953 | 4281-4582 | 5728-6100 | 6468-6828 | 7130-7374 |
| KX139462 | Jena 15-984/C1-like | 126-425 | 643-945 | 2125-2492 | 2737-3084 | 3432-3784 | 4112-4413 | 5559-5931 | 6299-6659 | 6961-7205 |
| JQ280307 | 3149/C2-like | 301-594 | 812-1114 | 2294-2661 | 2906-3253 | 3601-3953 | 4281-4582 | 5728-6100 | 6468-6828 | 7130-7374 |

Note: The regions of the putative recombination events event-1, event-2, event-3, event-4, event-5, event-6, event-7, event-8 and event-9 in the recombinant strain DL71 were respectively 310-607nt, 838-1140nt, 2330-2700nt, 2949-3296nt, 3686-4038nt, 4366-4667nt, 5814-6186nt, 6554-6914nt and 7216-7460nt in the alignment with gaps. According to the recombination events in DL71, the individual fragments in EV-A71 genomes without gaps were made certain. And the fragments in the genomes of DL71 and 19 reference strains within or between the recombinant regions were used in constructing the phylogenetic trees (Fig. 4, 5).

**Supplementary Table S6** Amino acid changes in the complete coding region between DL71 and other EV-A71 strains belonging to genotype C.

| Genes | Position | DL71 | C1 (4) | C1-like (10) | C2 (10) | C2-like (3) | C4 (170) | C3 (2) | C5 (2) |
| --- | --- | --- | --- | --- | --- | --- | --- | --- | --- |
| VP2 | 143 | N | D | D | D | D | D(169) N(1) | D | D |
|  | 144 | S | S | S | S(9), T(1) | S | T(143) S(27) | S | S |
|  | 146 | H | P | P | P | P | P(168) L(1) H(1) | P | P |
|  | 170 | I | I | V | I | I | I | I | I |
|  | 224 | Y | F | F | Y | Y | Y | F | F |
| VP3 | 39 | G | E | E | E | E | E(169) G(1) | E | E |
|  | 42 | H | N | N | N | N | N(169) H(1) | N | N |
|  | 67 | G | E | E | E | E | E(169) G(1) | E | E |
|  | 68 | G | R | R | R | R | R(169) G(1) | R | R |
|  | 81 | G | E | E | E | E | E(169) G(1) | E | E |
|  | 93 | H | N | D | S | S | N(150) S(10) D(8) Y(1) H(1) | S | S |
|  | 148 | A | T | T | T | T | T(169) A(1) | T | T |
|  | 159 | S | F | F | F | F | F(169) S(1) | F | F |
|  | 161 | C | L | L | L | L | L(169) C(1) | L | L |
|  | 232 | A | A | S | A | A | A | A | A |
| VP1 | 16 | V | V | M(9) V(1) | V | V | V(168) M(2) | V | V |
|  | 22 | R | Q | Q | R | Q | H(157) Q(12) R(1) | Q | Q |
|  | 31 | D | N | N | N(5) D(5) | N | N(163) H(2) D(5) | N | N |
|  | 249 | I | I | I | I | I | V(168) I(2) | I | I |
|  | 262 | I | I(3) V(1) | V | I | I | I(168) V(2) | V | V |
|  | 289 | T | T(3) A(1) | T(9) A(1) | A | A | A(161) V(1) D(1) T(7) | A | A |
| 2A | 25 | Y | H | H | H(9) Y(1) | H | H(165) Y(5) | H | H |
|  | 57 | N | N | N | N | N | D(150) H(1) A(1) N(18) | N | N |
|  | 66 | N | S | N | N | N | N(167) S(2) D(1) | N | N |
|  | 68 | R | M | M | M | M | M(133) R(37) | M | K |
|  | 82 | I | V(3) I(1) | I | I | V | I(164) V(6) | I | I |
|  | 83 | Y | Y | Y | F | F | Y(169) H(1) | F | F |
|  | 102 | V | V | V | V(9) A(1) | V | Q | V | V(1) L(1) |
|  | 131 | M | L | L | L(7) M(3) | L | L | L | L |
|  | 145 | D | D | E | D | D | E | D | D |
| 2B | 3 | S | S | S | S | T | S | S | S |
|  | 15 | M | M | T | M | M | T | M | M |
|  | 30 | N | H | N | N(7) S(3) | N | N(160) S(8) T(1) H(1) | S | N |
|  | 31 | H | H | H(9) Y(1) | H | H | Y(168) H(2) | H | H |
|  | 33 | V | I | I | I(9) V(1) | I | I(165) V(5) | I | I |
|  | 41 | K | R(3) K(1) | K | K | R | K | K | K |
|  | 47 | V | V | V | V | V | I(167) V(3) | V | V |
|  | 83 | I | I | I | V | I | I | I | I |
|  | 85 | A | S | S(9) L(1) | S | A | A(169) S(1) | S | S |
|  | 95 | I | M | M | M | I | I | M | M |
|  | 96 | A | A | A | A | V | A(168) V(2) | A | A |
| 2C | 11 | M | A | M | A | M | M | A | A |
|  | 13 | N | S | N | S(9) N(1) | N | N(168) S(2) | S | S |
|  | 21 | V | I | I | I | V | V(169) I(1) | I | I |
|  | 37 | V | I | I | V(5) I(5) | I | V(165) I(5) | I | I |
|  | 41 | K | K | K | K(5) R(5) | K | R(127) M(1) K(42) | K | R |
|  | 48 | N | N | S | N | N | N | N | N |
|  | 75 | A | A | A | A | A | V(163) I(6) A(1) | A | A |
|  | 76 | M | L | M | M | M | M | M | M |
|  | 108 | M | M | M | M | I | M(168) V(1) I(1) | M | M |
|  | 171 | V | V | V | I | V | V | V | I |
|  | 214 | T | A | T | T | T | T | T | T |
|  | 243 | Y | F | F | F | Y | Y | F | F |
|  | 257 | D | D | D | E | D | D(168) G(1) E(1) | D | D |
|  | 268 | K | R | K(9) R(1) | R | K | K(162) R(8) | R | R |
|  | 288 | E | K | K | K | K | K(169) E(1) | K | K |
|  | 297 | F | S | S | S | S | S(169) F(1) | S | S |
|  | 306 | V | V | V | V | I | V | V | V |
|  | 315 | S | N | N | N | N | S(168) N(1) G(1) | N | N |
|  | 316 | N | S | N | N | N | N(159) T(10) S(1) | N | N |
|  | 319 | A | A | A | V(8) A(2) | T | A | A | T |
| 3A | 3 | L | P | P | P | L | P(166) L(4) | P | P |
|  | 32 | K | E | E | E | E | E(169) K(1) | E | E |
|  | 33 | C | V | V | V | V | V(169) C(1) | V | V |
|  | 34 | A | R | R | R | R | R(169) A(1) | R | R |
|  | 35 | S | Q | Q | Q | Q | Q(169) S(1) | Q | Q |
|  | 36 | T | Y | Y | Y | Y | Y(169) T(1) | Y | Y |
|  | 37 | A | C | C | C | C | C(169) A(1) | C | C |
|  | 39 | D | E | D | D | D | D(168) E(1) G(1) | D | D |
|  | 44 | I | V | I | V | I | I | V | V |
|  | 46 | E | D | E | D | E | E(169) D(1) | D | D |
|  | 47 | A | T | T | S | T | A(115) T(46) V(9) | T | T |
|  | 56 | N | S | N | N(6) S(4) | N | N | N | G |
|  | 61 | V | I | V | I | V | V(166) I(4) | I | I |
|  | 65 | I | V | I | V | I | I(168) V(2) | V | I |
| 3B | 8 | R | K | K | K | K | K(163) R(7) | K | K |
|  | 10 | V | T | T | T | V | V(164) A(3) M(2) L(1) | T | T |
|  | 15 | A | V | V | V | V | A(146) V(16) T(8) | V | V |
| 3C | 2 | S | P | P | P | P | P(169) S(1) | P | P |
|  | 3 | D | S | S | S | S | S(167) N(2) D(1) | S | S |
|  | 33 | R | H | R | H(7) R(3) | R | R | H | H |
|  | 49 | V | V(3) I(1) | I | V | V | I(141) V(29) | V | V(1) I(1) |
|  | 55 | N | R(2) K(2) | N | K | N | N(169) S(1) | K | R |
|  | 56 | I | I | V | I(9) V(1) | V | V(156) I(14) | I | I |
|  | 57 | L | V | L | V | L | L(169) R(1) | V | V |
|  | 75 | I | V(3) I(1) | V | V | V | I(162) V(8) | V | V |
|  | 88 | K | K(3) R(1) | K | R(9) K(1) | K | K(168) R(2) | R | K |
|  | 93 | N | T | S | T | S | N(165) S(5) | T | T |
|  | 96 | T | P | G | P | A | T(166) A(4) | P | T |
|  | 111 | P | S | S | S(8) N(2) | S | S(169) P(1) | S | S |
|  | 153 | A | A | S | A | S | S | A | A |
|  | 157 | V | V | I | V | V | V(152) I(18) | V | V |
|  | 173 | A | A | S | A | G | G | A | A |
|  | 177 | G | G | G | G | S | S | G | G |
|  | 180 | C | C | A | C | A | A(168) V(2) | C | C |
|  | 181 | S | S | S | S | T | S | S | S |
| 3D | 6 | M | M | V | M | V | V | M | V |
|  | 16 | N | N | N | N | S | N(168) S(2) | V | V |
|  | 37 | T | T | N | T | N | N(130) S(38) D(2) | T | T |
|  | 44 | T | T | H | T | H | H | T | T |
|  | 68 | Y | H | H | H | H | H(165) Y(5) | H | H |
|  | 73 | Y | F | Y | F | Y | Y | F | F |
|  | 74 | I | V | I | V | I | I(164) V(6) | V | V |
|  | 75 | K | R | R | K | K | K(151) I(10) R(9) | R | R |
|  | 89 | E | D | E | D | D | E(161) D(4) G(4) S(1) | D | D |
|  | 91 | N | K | D | K | D | N(153) D(16) I(1) | D | K |
|  | 93 | S | T | S | T | S | S(168) T(2) | T | T |
|  | 94 | Q | K | Q | K | Q | Q(168) R(1) L(1) | K | K |
|  | 98 | E | E | E | E | D | E | E | E |
|  | 99 | E | D | E | D | E | E | D | D |
|  | 105 | E | E | D | E(9) A(1) | E | E(162) D(6) A(1) G(1) | E | E |
|  | 164 | S | A | S | A(8) T(2) | S | S(169) P(1) | A | A |
|  | 165 | I | I | M(9) I(1) | I | I | I(162) V(7) L(1) | I | L |
|  | 190 | T | T | A | T | T | A(159) V(5) T(6) | T | A |
|  | 197 | A | T(3) I(1) | T | A(9) V(1) | A | A(136) T(20) V(14) | T | T |
|  | 251 | I | I | L | I | L | L(168) M(1) V(1) | I | I |
|  | 261 | D | E | E | D | E | E(143) G(27) | D | D |
|  | 263 | V | V | V | V(9) L(1) | I | I(119) V(51) | V | V |
|  | 279 | T | K | K | K(5) T(5) | K | K(169) R(1) | K | K |
|  | 308 | A | T | A | T | A | A(169) T(1) | T | T |
|  | 368 | N | T | N | T | N | N(169) T(1) | T | T |
|  | 370 | E | E | E | E | G(2) D(1) | G(161) E(5) S(2) D(2) | E | E |
|  | 383 | E | H | E | H | D | E | Y | H |
|  | 392 | I | T | T | T | T | T(168) I(2) | T | T |
|  | 396 | K | K | K | R | K | R(132) S(30) G(1) K(7) | R | K(1) R(1) |
|  | 410 | S | N | N | S(9) N(1) | N | N(167) S(3) | N | N |
|  | 428 | E | E | Q | E | Q | Q | E | E |
|  | 442 | I | I | V | I | V | V(164) I(6) | V | V |
|  | 451 | F | F | Y | F(7) Y(3) | Y | Y | F | F |

Note: The amino acid sequences in the complete genome of DL71 were compared with 4 subgenotype C1 EV-A71 strains (DQ452074, DQ341361, AB575937, JF738000), 10 C1-like strains (KU641501-KU641508, KX139462, KY888026), 10 C2 strains (AF304457, KX372324, MG214681, AF304458, AF304459, DQ341357, HQ647176, JN835312, JN992283, KC436270), 3 C2-like strains (HM622391, HM622392, JQ280307), 2 C3 strains (DQ341356, DQ341355), 2 C5 strains (EF063152, EU527983), and 170 C4 strains. The detailed information of these EV-A71strains was listed in the supplementary Table S2. The numbers in the parentheses represented the number of EV-A71 strains. Except the strain SHZH98 (AF302996), 2 amino acid residues (143N and 146H) in VP2, 9 residues (39G, 42H, 67G, 68G, 81G, 93H, 148A, 159S, and 161C) in VP3, 2 residues (288E and 297F) in 2C, and 3 residues (2S, 3D, and 111P) in 3C were unique to DL71. And except the strain HQ09231463 (JQ316638), 6 amino acid residues (32K, 33C, 34A, 35S, 36T, and 37A) 3A were unique to DL71.
